# *PeptideManager*: a peptide selection tool for targeted proteomic studies involving mixed samples from different species

**DOI:** 10.3389/fgene.2014.00305

**Published:** 2014-09-02

**Authors:** Kevin Demeure, Elodie Duriez, Bruno Domon, Simone P. Niclou

**Affiliations:** ^1^NorLux Neuro-Oncology Laboratory, Department of Oncology, Centre de Recherche Public de la SantéLuxembourg, Luxembourg; ^2^LCP, Luxembourg Clinical Proteomics Center, Centre de Recherche Public de la SantéStrassen, Luxembourg

**Keywords:** mass spectrometry, targeted proteomics, rodent xenografts, human glioblastoma, mixed samples, automated tool, unique peptide selection

## Abstract

The search for clinically useful protein biomarkers using advanced mass spectrometry approaches represents a major focus in cancer research. However, the direct analysis of human samples may be challenging due to limited availability, the absence of appropriate control samples, or the large background variability observed in patient material. As an alternative approach, human tumors orthotopically implanted into a different species (xenografts) are clinically relevant models that have proven their utility in pre-clinical research. Patient derived xenografts for glioblastoma have been extensively characterized in our laboratory and have been shown to retain the characteristics of the parental tumor at the phenotypic and genetic level. Such models were also found to adequately mimic the behavior and treatment response of human tumors. The reproducibility of such xenograft models, the possibility to identify their host background and perform tumor-host interaction studies, are major advantages over the direct analysis of human samples. At the proteome level, the analysis of xenograft samples is challenged by the presence of proteins from two different species which, depending on tumor size, type or location, often appear at variable ratios. Any proteomics approach aimed at quantifying proteins within such samples must consider the identification of species specific peptides in order to avoid biases introduced by the host proteome. Here, we present an in-house methodology and tool developed to select peptides used as surrogates for protein candidates from a defined proteome (e.g., human) in a host proteome background (e.g., mouse, rat) suited for a mass spectrometry analysis. The tools presented here are applicable to any species specific proteome, provided a protein database is available. By linking the information from both proteomes, *PeptideManager* significantly facilitates and expedites the selection of peptides used as surrogates to analyze proteins of interest.

## Introduction

Mass spectrometry(MS)-based proteomics provides various approaches (i.e., shotgun, supervised and targeted approaches) (Domon and Aebersold, 2010) in the field of cancer research (Smith, 2012; Deracinois et al., 2013; Marx, 2013) and is nowadays widely used in pre-clinical and clinical investigations (Lee et al., 2011), and for biomarker studies (Li et al., 2011; Meng and Veenstra, 2011; Pan et al., 2012; Waldemarson et al., 2012).

Shotgun proteomics approach is the pipeline followed when biomarker discovery is considered; i.e., protein identification (Eng et al., 1994; Nesvizhskii, 2007) and label-free relative quantification (Bantscheff et al., 2007; Asara et al., 2008; Neilson et al., 2011). Regarding the evaluation (and validation) of biomarker candidates, targeted proteomics is considered for the precise and even the absolute quantification of these candidates (Gallien et al., 2011; Whiteaker et al., 2011; Gillette and Carr, 2013; Marx, 2013). Targeted proteomics is also increasingly used to perform supervised discovery of selected biomarker candidates (Gillette and Carr, 2013; Kim et al., 2013; Marx, 2013; Percy et al., 2014).

In cancer research, the direct analysis of human samples are often hampered because of strong inter-patient variability and limited sample availability. The latter is particularly true for samples requiring invasive sample collection procedures (e.g., biopsies) and is even worse for samples from healthy donors (control samples) (Pesch et al., 2014). Moreover, patient samples are normally limited to one time point and they do not offer the opportunity for a controlled interventional study. To circumvent those restrictions, animal models consisting in the orthotopical implantation of human tumors into animals (xenografts) have proven their utility as relevant models in many studies (Whiteaker et al., 2007; Huszthy et al., 2012; Tang et al., 2012; Klink et al., 2013). In the search for more biomarkers and more effective treatment options against brain tumors, our lab has developed patient derived xenografts for human glioblastoma in immunodeficient mice and rats (Wang et al., 2009). Those animal models have been extensively characterized and shown to retain the characteristics of the parental tumor at the phenotypic and genetic level (Niclou et al., 2008; Rajcevic et al., 2009; Golebiewska et al., 2013). Such models were also found to adequately mimic the behavior and treatment response of human tumors (Keunen et al., 2011). Another major advantage of these models over human samples is their reproducibility and their identifiable host background allowing a detailed analysis of tumor-host interactions. The use of xenograft samples also gives an easy and direct access to control samples exhibiting a more controlled experimental setup compared to human samples.

In bottom-up MS-based proteomics, peptides are generally used as the representative of the proteins either for identification or quantification purposes (Aebersold and Mann, 2003; Chait, 2006). Peptides are identified and monitored via their mass-to-charge ratio and their fragmentation pattern produced under collision induced dissociation (CID) (peptide ion-types, mass-to-charges and relative intensities) (Eng et al., 1994; Nesvizhskii, 2007). Therefore, it is desirable to target a few representative peptides for each protein of interest. The choice of these peptides used as protein surrogates is therefore crucial to unequivocally identify and quantify the protein of interest. A peptide is considered as proteotypic (Kuster et al., 2005; Mallick et al., 2007) when it fulfills two selection criteria: it is a unique representative of a single protein within the proteome of interest and it possesses a good MS detectability (Brownridge and Beynon, 2011). Such peptides allow umambiguous analysis of the targeted protein. However, the selection of proteotypic peptides is often tedious and time-consuming. It has to carefully take into account several essential criteria (Gallien et al., 2011) including uniqueness of peptide sequence, protein digestion efficiency, enzymatical and chemical modifications at the protein or peptide level, physicochemical behavior of the peptide.

**Figure 1:**
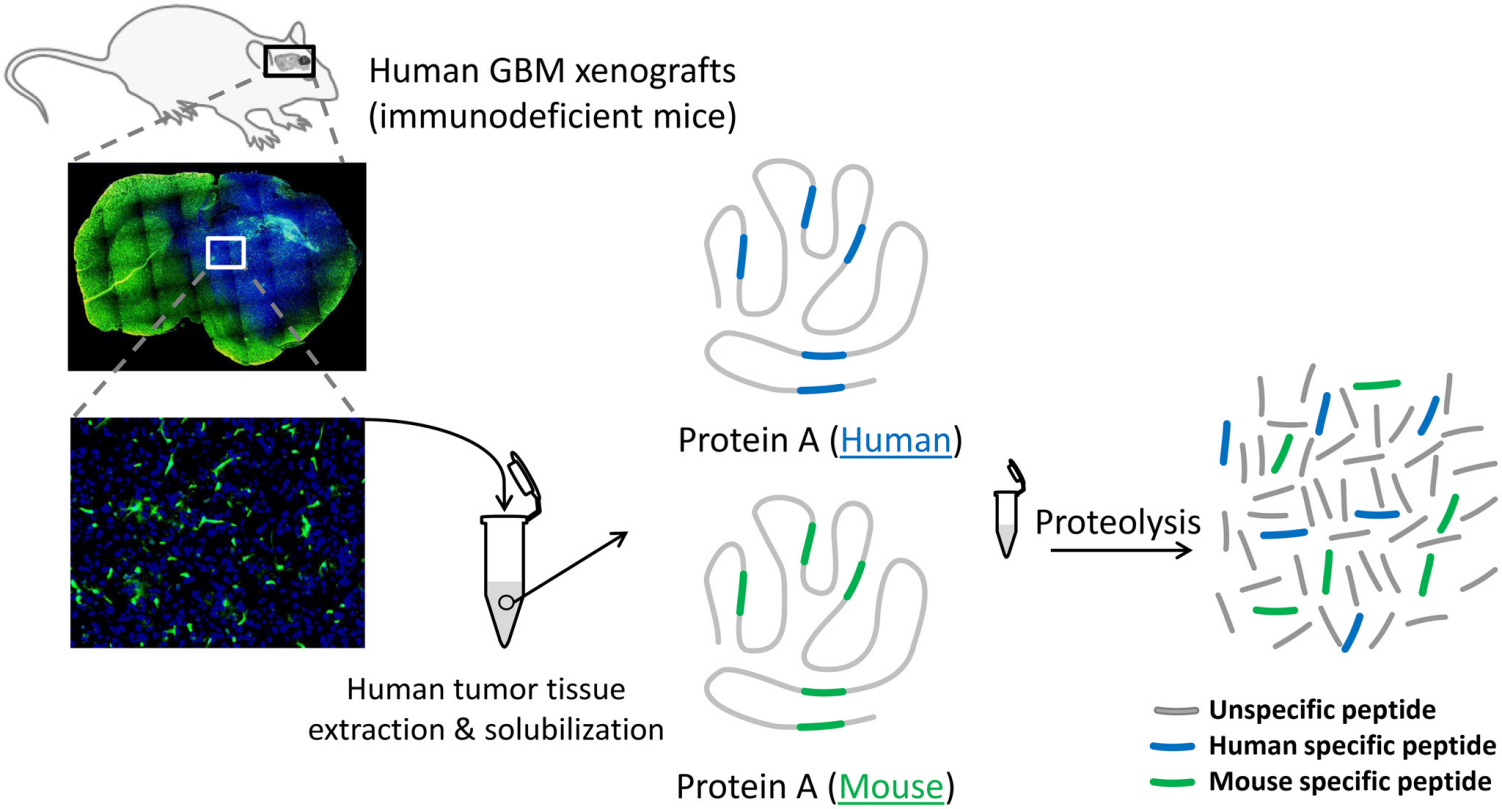
**Schematic of the protein extraction procedure from a human GBM xenografted in a GFP-expressing immunodeficient mouse**. Since the excision of the human tumor tissue (in blue) includes a variable proportion of mouse cells (in green), the surrogate peptide selection process of a given protein A must exclusively consider the human specific peptide (blue peptides). Monitoring protein A via human specific peptides in different tumor pieces will eliminate the bias induced by the presence of mouse proteins within the samples.

In the context of xenograft samples, the use of MS-based targeted (or supervised) proteomics approaches for protein biomarker studies needs to consider the presence of various proteomes from different species. This leads to more complex samples and a more constrained and restricted choice of the surrogate peptides (Figure 1). The tool *PeptideManager* was built with the purpose of collecting and combining information from different public protein databases [UniProt (SwissProt or TrEMBL or both (UniProt)), RefSeq or IPI] and/or species to facilitate and speed up the surrogate peptide selection compared to the manual selection process. The proposed software is specially designed for the selection of surrogate peptides in the cases involving various species proteomes such as in xenografts. Obviously, it can also be used in the general case when a single proteome is implicated.

## General consideration on peptide selection

The separation, detection and characterization by liquid chromatography (LC)-MS/MS of a proteotypic peptide of a given protein allows for confident identification given that the peptide identification is reliable (via the m/z of its precursor and of its fragments). However, for quantitative purposes, the choice of a surrogate peptide for a given protein is not trivial since the sequence uniqueness of the peptide is not the only pre-requisite (Gallien et al., 2011). Indeed, several parameters need to be considered to warrant the selection of peptides that reliably represent the targeted protein at the quantitative level (Lange et al., 2008; Gallien et al., 2011). Such peptides are termed proteotypic (Kuster et al., 2005; Mallick et al., 2007) and are characterized by two major conditions; uniqueness of its sequence within the proteome of interest and a good detectability in LC-MS(/MS). The good detectability of peptides in MS is influenced by its physicochemical properties and other experimental-related parameters, which are described hereafter.

Ideally, the enzymatic digestion of the proteins should not introduce any bias. However, since the yield of an enzymatic reaction is rarely complete, the enzymatic digestion itself introduces some variability across samples (Brownridge and Beynon, 2011; Loziuk et al., 2013). This can be limited in two ways. Firstly, a precise control of the experimental conditions will limit the variability across sample replicates. Secondly, since the digestion efficiency is not identical for all the peptides derived from the same protein, a rational choice of the peptides exhibiting the best cleavage propensities should be performed. The yield of the proteolytic cleavage depends on the protease and on the amino acid composition around the cleavage sites. The cleavage efficiencies can be estimated theoretically for some proteases and various web-tools are freely available to predict which proteolytic peptide bonds are likely to be missed by the standard enzymes [e.g., PeptideCutter web-tool on ExPASy (http://web.expasy.org/peptide_cutter/) (Eyers et al., 2011; Artimo et al., 2012; Lawless and Hubbard, 2012)].

The reduction and the alkylation steps of the cysteines in proteins are intended to make cleavage sites accessible that would otherwise be hindered. Nevertheless, as any chemical reaction, this sample preparation procedure can introduce unpredictable biases because of an incomplete reaction. Therefore, peptides containing amino acids that are prone to chemical modification [e.g., cysteines (incomplete reduction/alkylation) and methionine (partially oxidized)] should be avoided (Bischoff and Schluter, 2012). Correspondingly, amino acids with the potential for enzymatic post-translational modifications (PTMs) should be avoided if the analysis of those modifications is not in the scope of the study. Peptides containing sequence uncertainties (e.g., sequence conflicts between public protein databases) or amino acids showing variability due to single-nucleotide polymorphisms (SNPs) should be discarded as well.

Depending on whether all isoforms or a particular isoform of a given protein are of interest, either the selected peptides should be representative of all the isoforms of the protein or the selected peptides should be representative of only one specific protein isoform.

The mass-to-charge ratio of the selected peptide should comply with the mass range restriction of the mass spectrometer(s) that will be used for the subsequent analyses (e.g., m/z between 400 and 1600 on a triple quadrupole platform). Moreover, very short peptides are less likely to have a unique sequence and they are more susceptible to interferences. On the other hand, lengthy peptides are not desirable due to their hydrophobicity and to issues regarding their synthesis and purification. Typically, peptide ranging from 5 to around 22 amino acids are best suited for quantification purpose (Gallien et al., 2011).

Various tools are currently available for the theoretical or semi-empirical estimation of the proteotypic properties of a peptide sequence including the digestion efficacy according to the cleavage site (Kuster et al., 2005; Mallick et al., 2007; Fusaro et al., 2009; Eyers et al., 2011; Lawless and Hubbard, 2012; Mohammed et al., 2014; Qeli et al., 2014). However, these tools do not handle samples containing proteins from multiple species that therefore require a very time-consuming manual selection of surrogate peptide candidates. The proposed tool was therefore intended to automate this selection step of unique peptide candidates by combining all relevant information present in public protein databases to help the filtering of inappropriate peptides (e.g., peptide sequence present in both proteomes).

**Figure 2:**
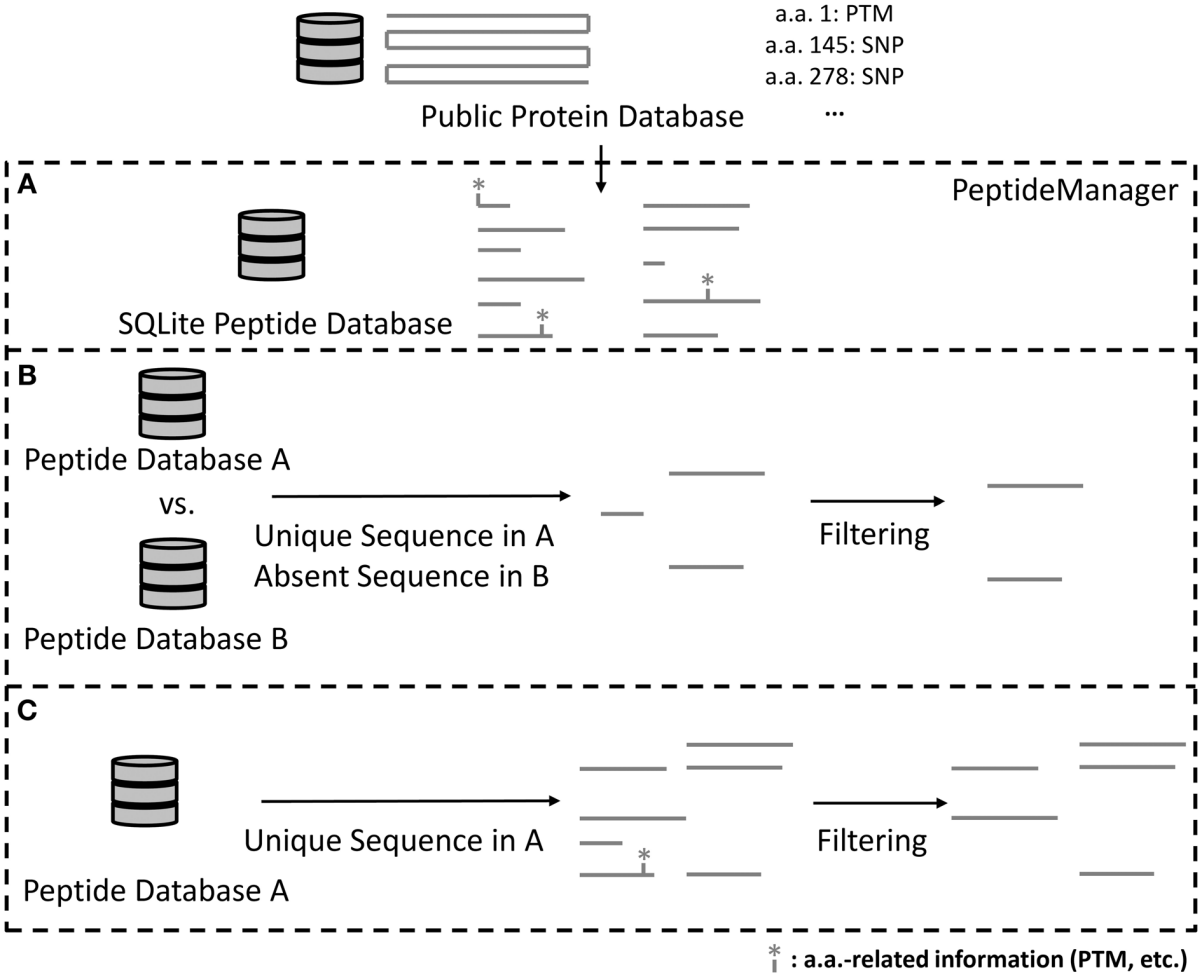
**Scheme illustrating how *PeptideManager* is operating**. Required information from the public database is extracted to produce the peptide database (SQLite format) **(A)**. Created peptide databases are stored and can be used for peptide selection queries **(B,C)**. The peptide selection can be done with **(B)** or without **(C)** the presence of a background proteome. It is noteworthy that the presence of a background proteome (as in the case of xenografts) **(B)** will generally reduce the number of surrogate peptide candidates compared to the list of peptides obtained in **(C)**.

The tool *PeptideManager* was designed to: (1) build and store peptide databases from public repositories, (2) link available information (e.g., PTM sites, SNPs, signal peptide) from public databases at the peptide level, (3) allow queries and peptide pre-selection within those databases, and most importantly (4) perform peptide pre-selection within a given proteome of interest while taking into account the presence of another species proteome within the sample (Figure 2).

## Materials and methods

Glioblastoma samples were collected at the Neurosurgery Department of the Center Hospitalier in Luxembourg (CHL) from patients having given their informed consent. Collection and use of patient tumor material has been approved by the National Ethics Committee for Research (CNER) of Luxembourg. All animal procedures were approved by the national authorities responsible for animal experiments in Luxembourg.

## Peptide database creation

The peptide selection tool *PeptideManager* was written in C# language and the GUI (graphical user interface) was designed with Visual Studio Express 2010. The databases were built in the SQLite format (http://www.sqlite.org/about.html). *PeptideManager* can be run on any computer on which the Microsoft.NET framework is installed. *PeptideManager* and its user guide are freely available at http://peptidemanager-lrno.sourceforge.net.

The peptide databases (in SQLite format) derived from different public protein databases can be built by *PeptideManager*, namely UniProt (Uniprot, 2009, 2014; Magrane and Consortium, 2011), RefSeq (Pruitt et al., 2007, 2012, 2014) and IPI (Kersey et al., 2004). The different file formats requested to build a peptide database are indicated in Table 1. Protein sequences are digested *in silico* by the proteolytic enzyme selected (trypsin, Lys-C, Arg-C or Lys-N). The required information present in the public database is extracted and inserted into the *PeptideManager* database. If present in the public protein data repository, information about PTMs, sequence conflicts or other sequence related modifications are extracted and inserted at the peptide level in the newly generated database. In order to limit the size of the created peptide database, a minimal peptide length (3–5 amino acids) as well as the number of allowed miscleavages (0–2) can be selected. Once created, the database is indexed in *PeptideManager* and is immediately available for search requests.

**Table 1:** **List of the different file formats supported by *PeptideManager* for the different public protein database repositories**.

	**File format(s) supported**
SwissProt/TrEMBL/Uniprot	*.txt, *.dat
RefSeq	*.faa^a^, *.gpff^b^
IPI	*.dat

a *FASTA-like file format that only contains protein sequences and essential protein information*.

b *More complete file format than.faa files. They include additional information such as post-translational modifications (PTMs), single nucleotide polymorphisms (SNPs), etc*.

Contrary to similar tools like PeptidePicker (Mohammed et al., 2014), CONSeQuence (Eyers et al., 2011) or even Skyline (Maclean et al., 2010), *PeptideManager* is solely intended to build a peptide database, to allow search queries within these databases and to help the user to perform unique peptide selection for targeted experiments. The advantages of *PeptideManager* are to allow the user to define a host/background proteome, to import databases from UniProt, RefSeq and IPI, to extract all the available information (protein features) and to suggest default filters for the selection of unique peptide sequences (with or without the presence of a host/background proteome).

## Surrogate peptide selection

**Figure 3:**
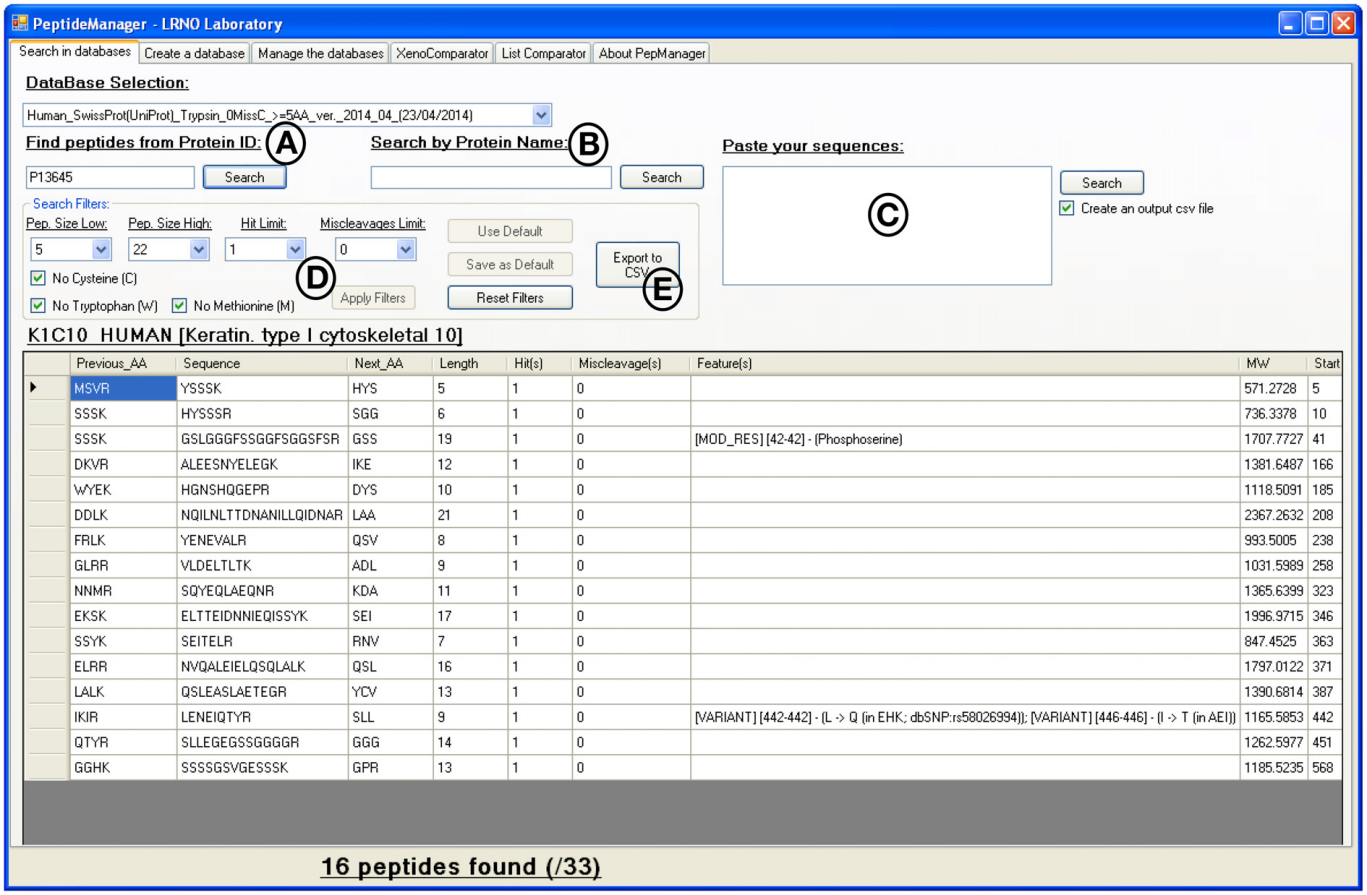
**Print screen of the results displayed by *PeptideManager* for the P13645 human protein**. Search queries can be performed by protein ID (A), by protein name (B) or by peptide sequence(s) (C). The list of peptides obtained can be filtered out according to the length (e.g., 5 a.a. ≤ peptide length ≤ 22 a.a.), the presence of unwanted amino acid (methionine-containing peptide for example) or the frequency of the peptide sequence within the database (D). The results can be exported in *csv* file format (E).

As shown in Figure 3, search queries can be performed with protein accession numbers (individually or in batch mode), protein name or peptide sequences (individually or in batch mode). The displayed results can be filtered against unsuitable amino acids or lengths for example. The results can be directly saved in *csv* file format. When only one species proteome is studied, this type of search request provides all the information needed to select surrogate peptide candidates (unique peptide sequences) for the given protein. Moreover, this type of request can be used to define the uniqueness of a list of detected peptides from experimental evidences and confirm the identification of the related proteins.

**Figure 4:**
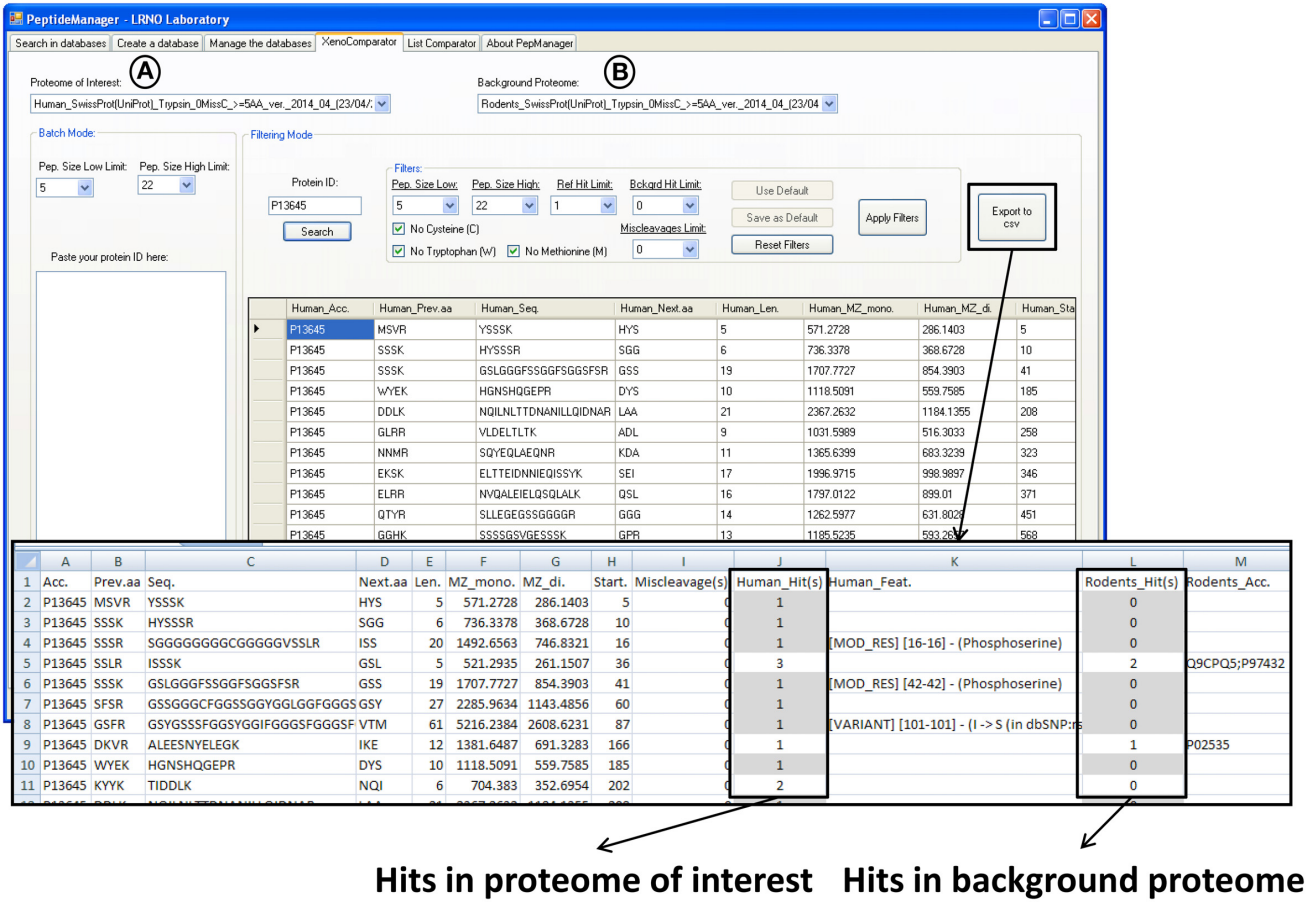
**Print screen showing the results obtained with *PeptideManager* for the human P13645 protein within the mouse proteome (host proteome)**. The information concerning both proteomes [selected proteomes in (A,B)] is brought together and the number of observation (hits) of the peptide sequence is indicated in each proteome. Human specific peptide candidates are those with one hit in the human proteome [selected in (A)] and no hit in the mouse proteome [selected in (B)] (e.g., HGNSHQGEPR). The results (filtered or not) can be saved in a *csv* file.

The major advantage of *PeptideManager* is to possess a search function specifically dedicated to peptide selection when various species proteomes are involved (Figure 1). In addition to the previous search request, the proteome of interest and the proteome of the host species (background proteome) can be selected (Figure 4). In this case, the information will be gathered by *PeptideManager* within the peptide databases of both proteomes. The protein accession number is used to extract all peptide information related to the targeted protein in the database of the proteome of interest. Subsequently, the peptide sequences are searched against the peptide database of the host species proteome to determine the number of occurrences (hits) within the background proteome. In order to monitor/quantify a protein of interest without any bias coming from the host/background proteome, only peptides that are unique sequences (i.e., unique representative of the protein of interest) within the proteome of interest and absent within the host/background proteome should be selected. An example of a result file (*csv* format) is partly displayed in Figure 4.

As mentioned, the software is not only intended for the selection of unique sequences of a given protein but also to perform any kind of search queries within the databases. In order to keep all the information available for the user, filters are available and can be used/or not according to the purpose of the query. However, in the case of the selection of unique peptide sequences (with or without the presence of a host proteome), it is possible to apply “advised default values” of the filters. A comprehensive user guide is provided as Supplementary Material. This user guide explains the different steps to use correctly the software and includes several case studies in order to exemplify different situations that users could meet, e.g., protein isoform differentiation, post-translational modification monitoring, unique peptide selection with or without the presence of a host/background proteome.

*PeptideManager* was used for the selection of surrogate peptides to develop SRM (selected reaction monitoring) assays on a triple-quadrupole platform for the targeted analysis of protein biomarker candidates. This was done in the context of human GBM xenografts (mice and rats) and *PeptideManager* greatly facilitated and expedited the selection of unique peptide from mixed samples involving different species proteomes. Compared to the few proteins per day for which unique peptide selection can be done manually, *PeptideManager* succeeded in performing the selection of peptide candidates for hundreds of proteins a day.

In order to further validate the selected peptides and/or decrease further the number of peptide candidates, additional information, not present in the public protein databases, such as the enzymatic digestion efficiencies and the LC-MS(/MS) behaviors of the peptide candidates can be used. From the selected peptides, predictive computational tools (Artimo et al., 2012; Lawless and Hubbard, 2012) can be used to filter out peptides arising from poorly efficient cleavage sites that would lead to an erroneous values of peptide/protein amount as well as a decrease in sensitivity. MS-based data from the proteomic community are freely available in public data repositories such as PeptideAtlas (Desiere et al., 2006), GPM Proteomics Database (Craig et al., 2004, 2005) or PRIDE (Vizcaino et al., 2013) that permit to estimate the MS-behaviors of peptide sequence candidates.

In these public databases, proteomics data can be retrieved by protein accession number, peptide sequence, species or even sample type (e.g., brain, kidney, liver). For a given protein, all the previously detected peptides identifying this protein are displayed; however they may not be unique and may contain miscleavages. Using the list of pre-selected unique peptide candidates from *PeptideManager* will allow to rapidly access the pertinent information from the data provided by those public repositories. Additional empirical and theoretical information (available in PeptideAtlas) may help to select the best candidates. For example, how many times a peptide sequence was detected within a given set of experiments can help to estimate the detectability of this peptide by LC-MS (due to digestion efficacy, good ionization, LC or MS behavior, etc.). Theoretical scores based on various parameters (e.g., amino acid composition and hydrophobicity) and algorithms can give theoretical estimations of the LC-MS behavior and detectability of the peptides (Mallick et al., 2007; Fusaro et al., 2009; Eyers et al., 2011; Qeli et al., 2014). All this information can be used to further rationalize the peptide selection and keep those peptides predicted to demonstrate the best LC-MS behavior and leading to the MS measurements with the highest possible sensitivity. If MS/MS spectra are available, they can be used to select the most appropriate charge state of the peptide to be monitored. In the context of the development of an SRM assay, the fragmentation spectra can be used to select the most intense fragments for a given peptide which should lead to an optimal sensitivity for that peptide when monitoring those transitions by SRM. Reference transitions for a large number of peptides are publicly available in the SRMAtlas repository (Picotti et al., 2008, 2013; Huttenhain et al., 2013). However, those transitions need to be validated (e.g., absence of interferences) within the biological samples of interest.

## Conclusions and perspectives

*PeptideManager* was used for the selection of unique peptide candidates as protein surrogates in the context of a supervised evaluation (SRM assays on a triple-quadrupole platform) of protein biomarker candidates in human GBM xenografts (mice and rats). This tool greatly facilitated and expedited the peptide selection for these mixed samples involving different species proteomes. Moreover, it is applicable to complex samples (with or without the presence of a host (background) proteome) of all species for which a protein database is available.

*PeptideManager* could be extended to include additional information helping to the peptide selection by directly inserting the information from MS-based proteomics data public repositories (e.g., PeptideAtlas) and the theoretical information about the enzymatic cleavage efficiency (e.g., PeptideCutter) within the peptide databases.

### Conflict of interest statement

The authors declare that the research was conducted in the absence of any commercial or financial relationships that could be construed as a potential conflict of interest.
