# Supplementary material for: PeptideManager: a peptide selection tool for targeted proteomic studies involving mixed samples from different species
Source: Front Genet. 2014 Sep 2;5:305. doi: 10.3389/fgene.2014.00305 (PMC4151198; doi:10.3389/fgene.2014.00305)
Supplement: Supplementary file 1 [file Presentation1.PDF]

<u>PeptideManager – User Guide</u>

CRP-Santé (Luxembourg)

LRNO – NorLux Neuro-Oncology Laboratory

Demeure Kevin
kevin.demeure@crp-sante.lu
kevindemeure@gmail.com

Table of Contents

Install PeptideManager 3

First Launch 3

Build a peptide database 5

Manage the PeptideManager databases 9

Launch a search request 13

Search by Protein ID 13

Search by Protein Name 17

Search by Peptide Sequence(s) 19

Case Studies: Select unique peptide sequences for a targeted proteomics experiment 20

Unique peptide sequences selection – P12352 20

Peptide selection to differentiate protein isoforms 24

Monitoring post-translational modifications of interest 25

Select unique peptide sequences with the presence of a host/background proteome 26

Filtering Mode 27

Batch Mode 33

Case study: Selection of unique peptide sequences for a targeted proteomics experiment with the presence of a background/host proteome 36

Download the public protein databases 38

SwissProt/TrEMBL/UniProt 38

Predefined taxonomic divisions protein databases 38

Other taxonomic divisions 40

RefSeq 42

IPI 44

Compare peptide/protein lists 46

Import a user-customized protein database 47

Install PeptideManager

Unzip the PeptideManager archive, double-click on the setup file and click on "**Install**" (Figure 1). A PeptideManager shortcut will be added to the Start Menu of Windows (in the Microsoft section on some computers).

It is important that the parameters of windows "*list separator*" and "*decimal symbol*" are set to "**,**" and "**.**", respectively ("Control Panel/Clock, Language and Region/Region and Language" and click on "**Additional Settings**").

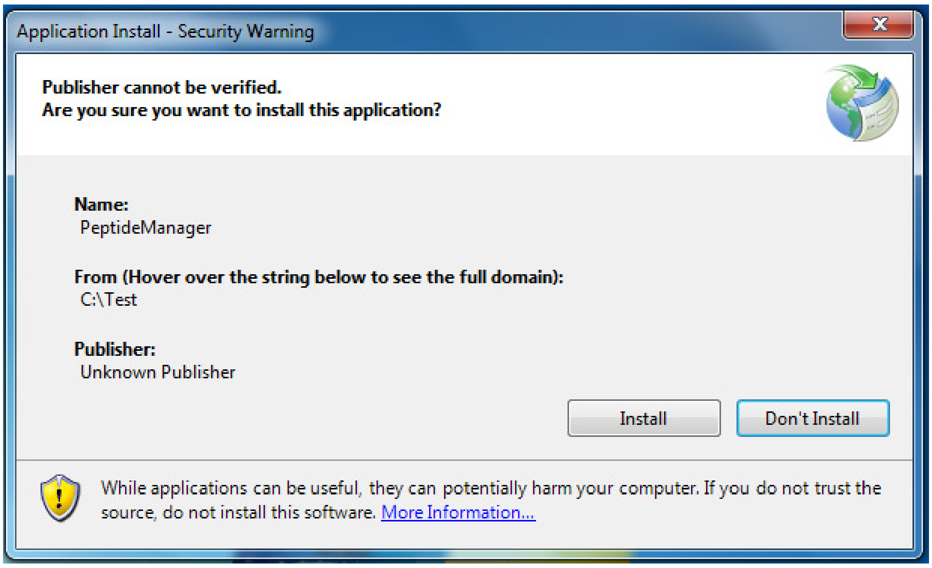

Figure 1.

First Launch

When PeptideManager is launched for the first time, the message "*No database found! Please create a database or add an existing database.*" is automatically displayed as shown in Figure 2. There is no database provided with PeptideManager. You have to import manually the databases.

There are two ways to import a database in PeptideManager: to build a new database from public protein data repositories (see Section "*Build a peptide database*" on Page 5 to learn how to create a PeptideManager database from protein databases found in the public data repositories) or to select an existing PeptideManager database (See Section "*Manage the PeptideManager databases*" on Page 9).

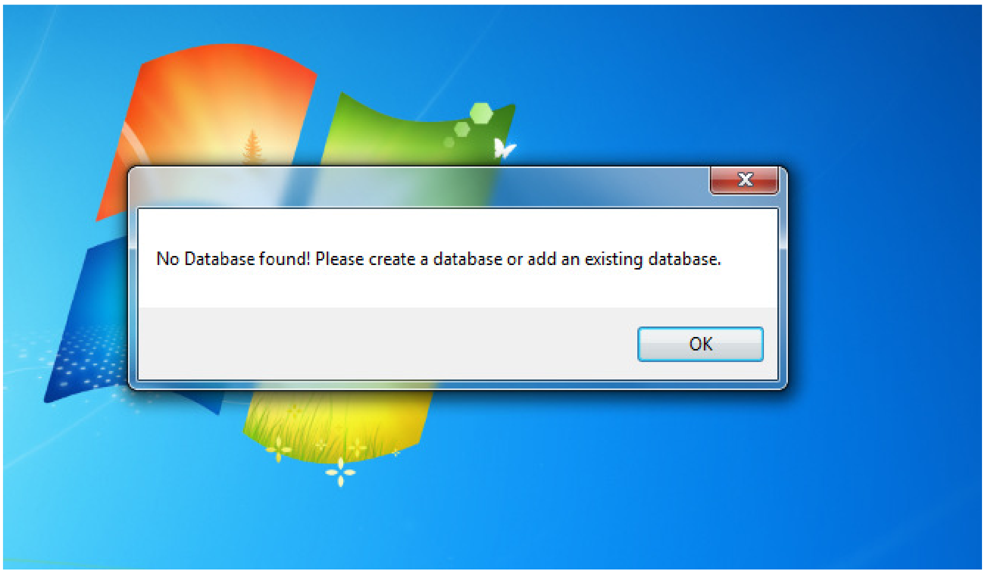

Figure 2.

Click on "**OK**" to reach the "*Manage the databases*" tab (Figure 3) where you can manage your PeptideManager databases and import existing ones (see Section "*Manage the PeptideManager databases*" on Page 9).

Choose the "*Create a database*" tab if you want to build a new PeptideManager database from public data repositories (see Section "*Build a peptide database*" on Page 5).

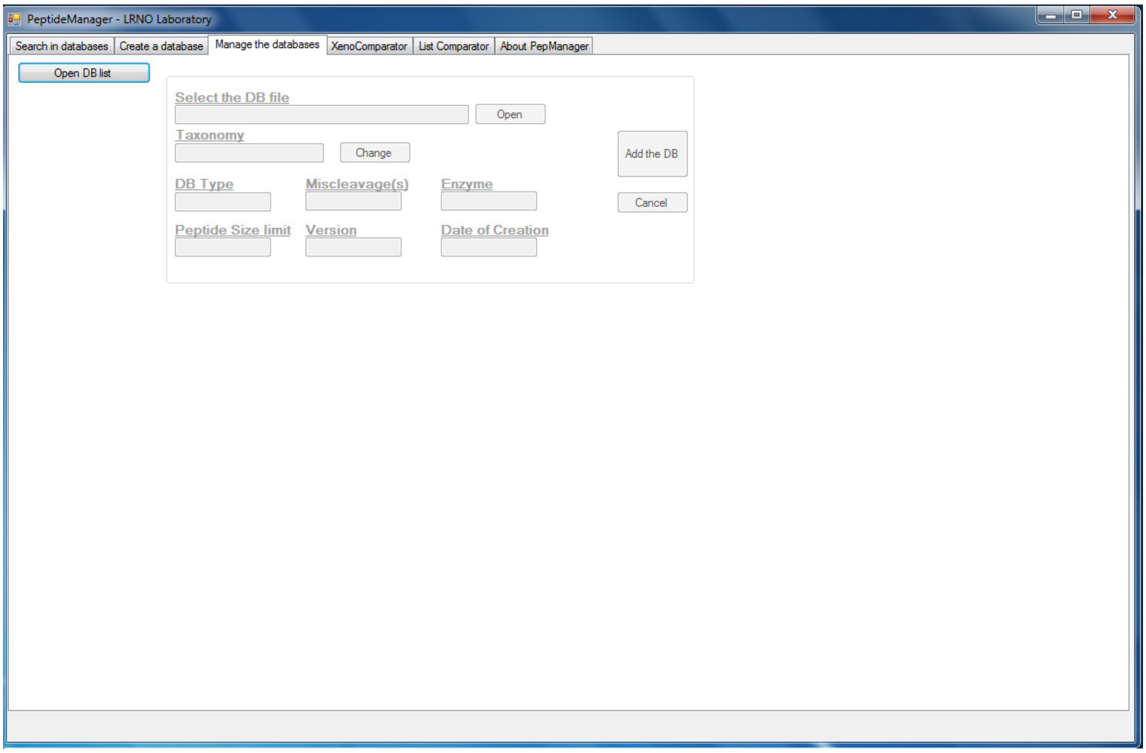

Figure 3.

Build a peptide database

In order to build a peptide database in PeptideManager, you need a data file (database) from a public protein data repository (SwissProt/TrEMBL/Uniprot, IPI or RefSeq) (see Section "*Download the public protein databases*" on Page 38 to learn how to download those data files) or to furnish your own data file in fasta-like format (see Section "*Import a user-customized protein database*" on Page 47 to learn how to create your own user-customized PeptideManager database).

In the "*Create a database*" tab (Figure 4), indicate the type of public database you want to import in PeptideManager, the enzyme to use for the *in silico* digestion of the protein sequences, the number of miscleavages allowed (especially in the case of Trypsin as enzyme, note that the number of miscleavages allowed increases significantly the size of the database), the low limit of the peptide length (the removal of peptides with length under 5 amino acids significantly decreases the size of the database). You can give an indication of the version of the database you are importing as well as the taxonomy/ies contained in the database.

Check the "*Create Output CSV file*" checkbox if you want to create a csv file containing the digest of the database and another one the protein list (accession and sequence). Check the "*Add directly the DB to the PeptideManager DB list*" checkbox if you want the database to be directly inserted in the database list of PeptideManager (see Section "*Manage the PeptideManager databases*" on Page 9 to learn how to manage your PeptideManager databases). In the case of Trypsin, the checkbox "*KP and RP are cleaved*" allows you to indicate if the KP and the RP cleavage sites are cleaved (unchecked) or not (checked) in the peptide database.

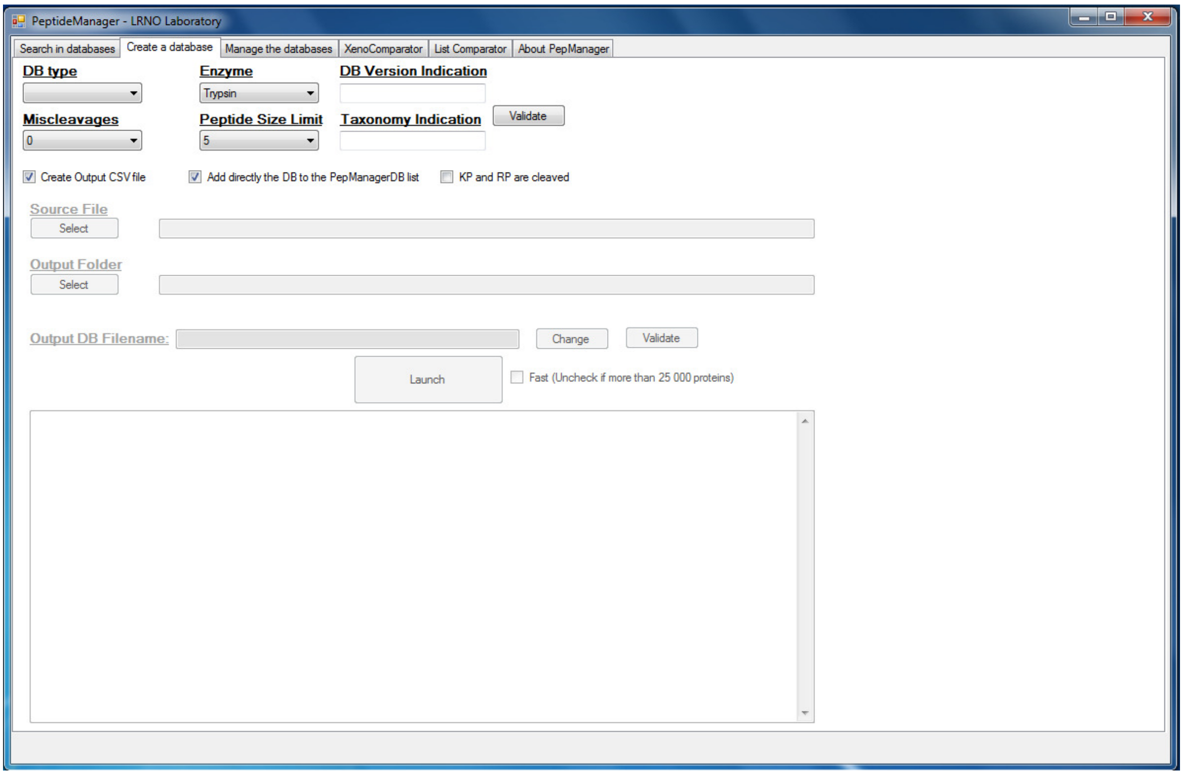

Figure 4.

Click on "**Validate**", select the source data file as well as the output folder of the database that will be built (Figure 5). See Sections "*Download the public protein databases*" on Page 38 and "*Import a user-customized protein database*" on Page 47 to learn how to download protein databases from the public data repositories or to build a user-customized database. Figure 6 indicates the file formats compatible with PeptideManager for the different public data repositories (A: SwissProt/TrEMBL/UniProt, B: RefSeq and C: IPI).

A suggested database filename (containing the pertinent information about the database) is displayed in the "*Output DB Filename*" textbox. You can modify it by clicking on "**Change**". Click on "**Validate**" to go to the next step (Figure 7).

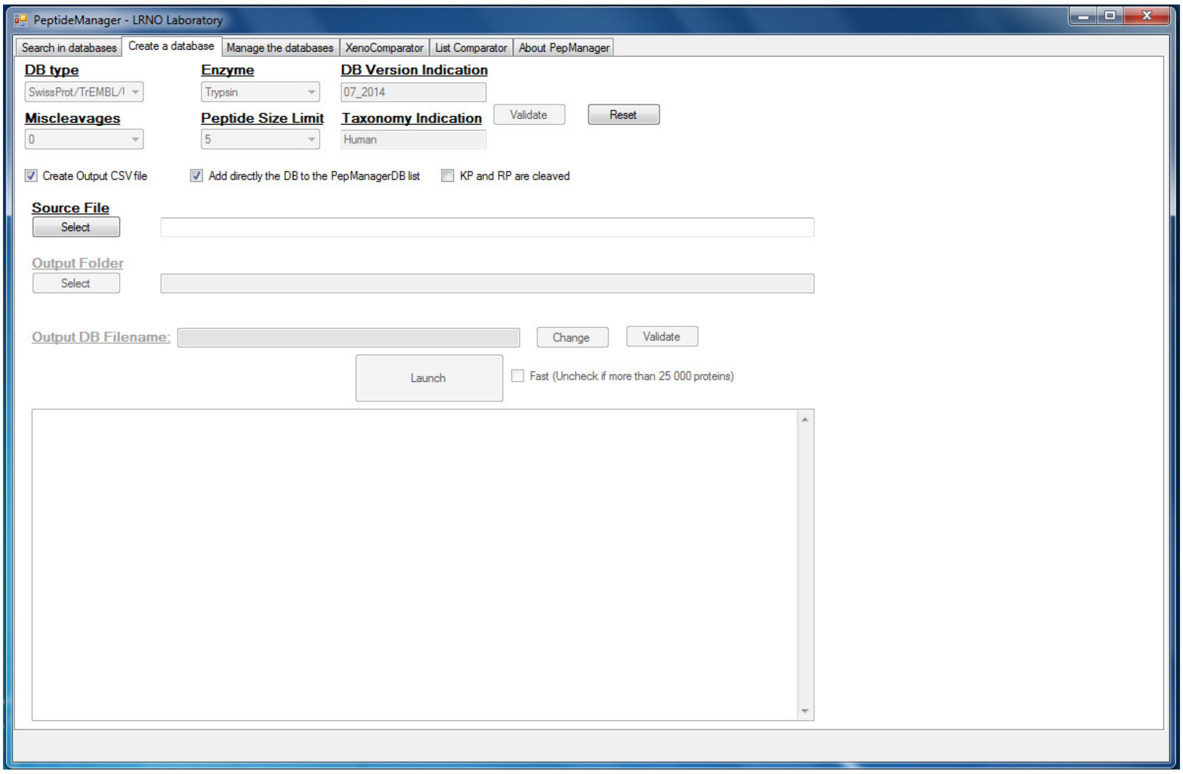

Figure 5.

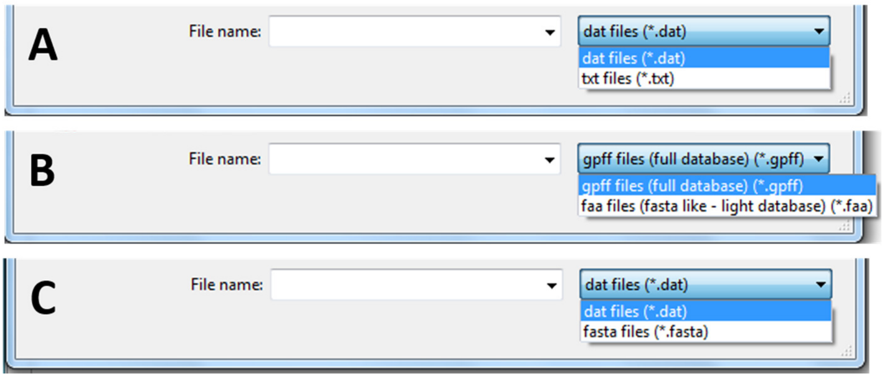

Figure 6. A: SwissProt/TrEMBL/UniProt, B: RefSeq and C: IPI.

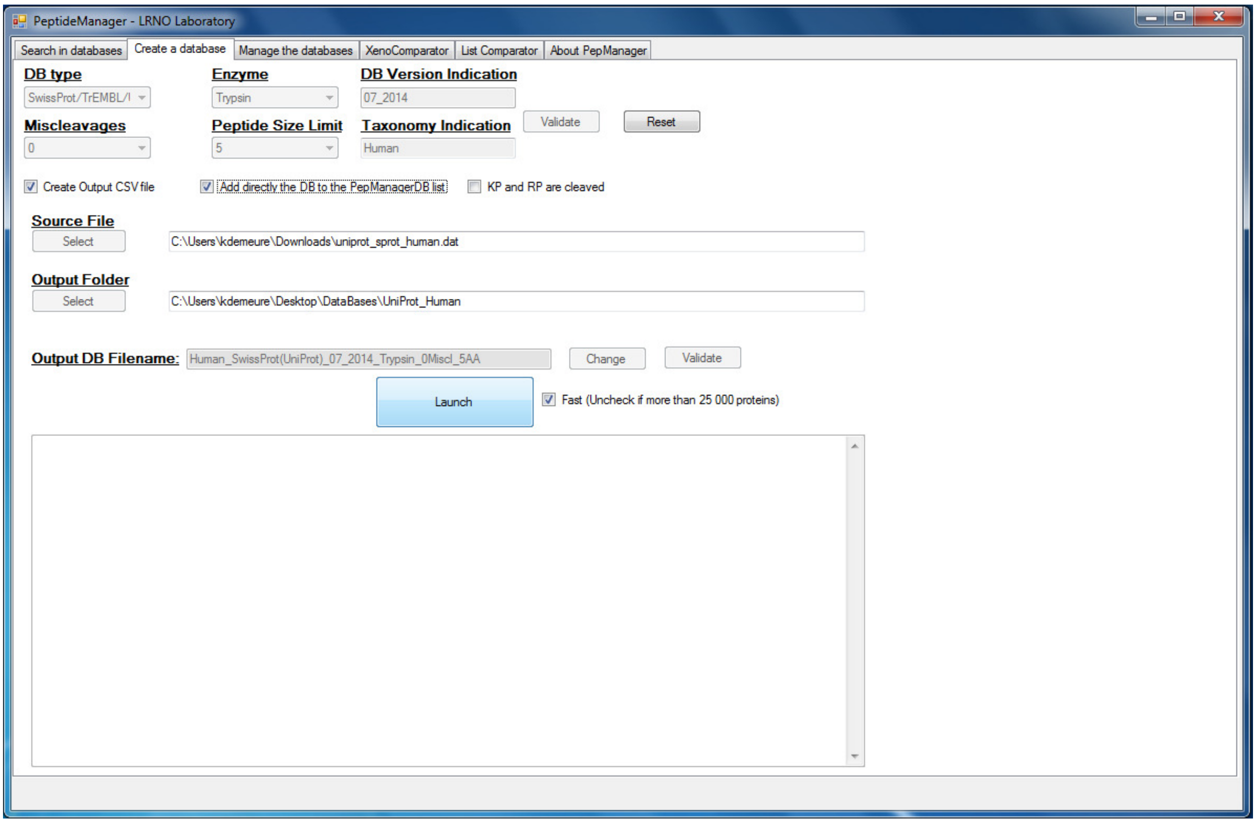

Figure 7.

PeptideManager has now all the required information to build the database, you can launch the creation of the database by clicking on “**Launch**” (Figure 8).

The creation of the database can be a highly memory-demanding process when huge databases are built. By unchecking the “*Fast (Uncheck if more than 25 000 proteins)*” checkbox, you will decrease the memory-demand of PeptideManager during this step (slower process) to allow you to continue a parallel task with your computer.

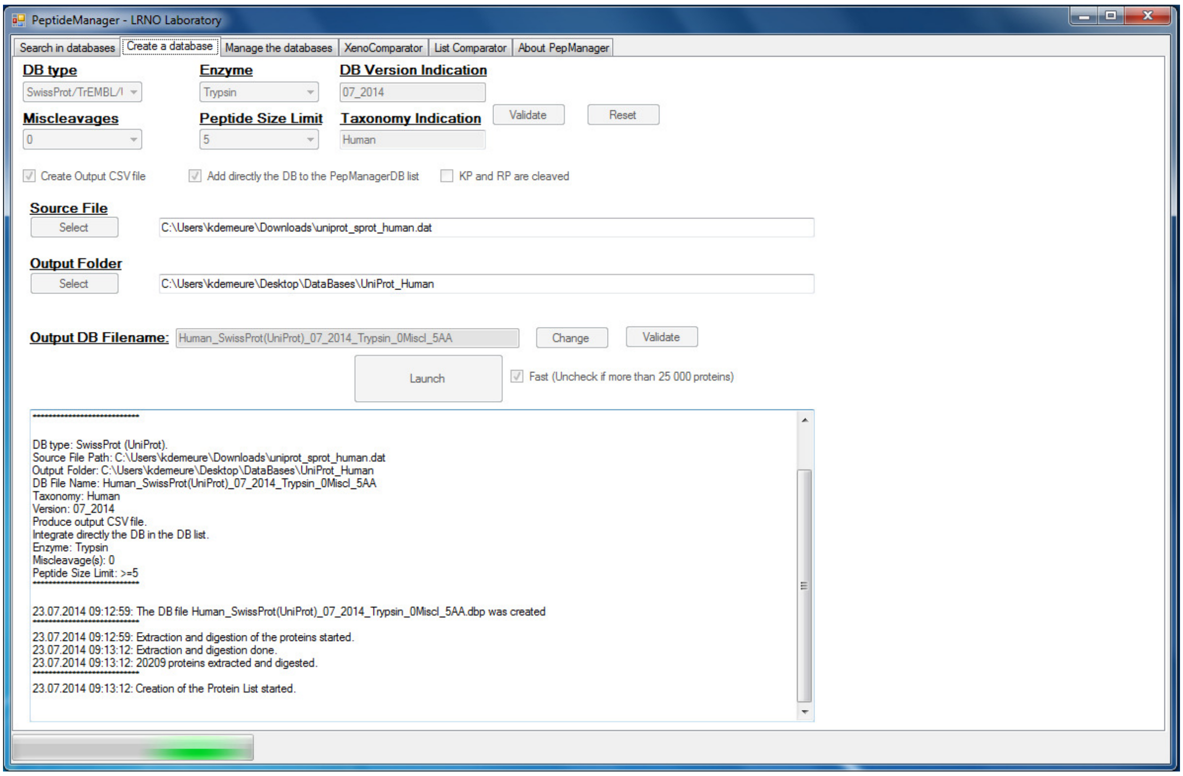

Figure 8.

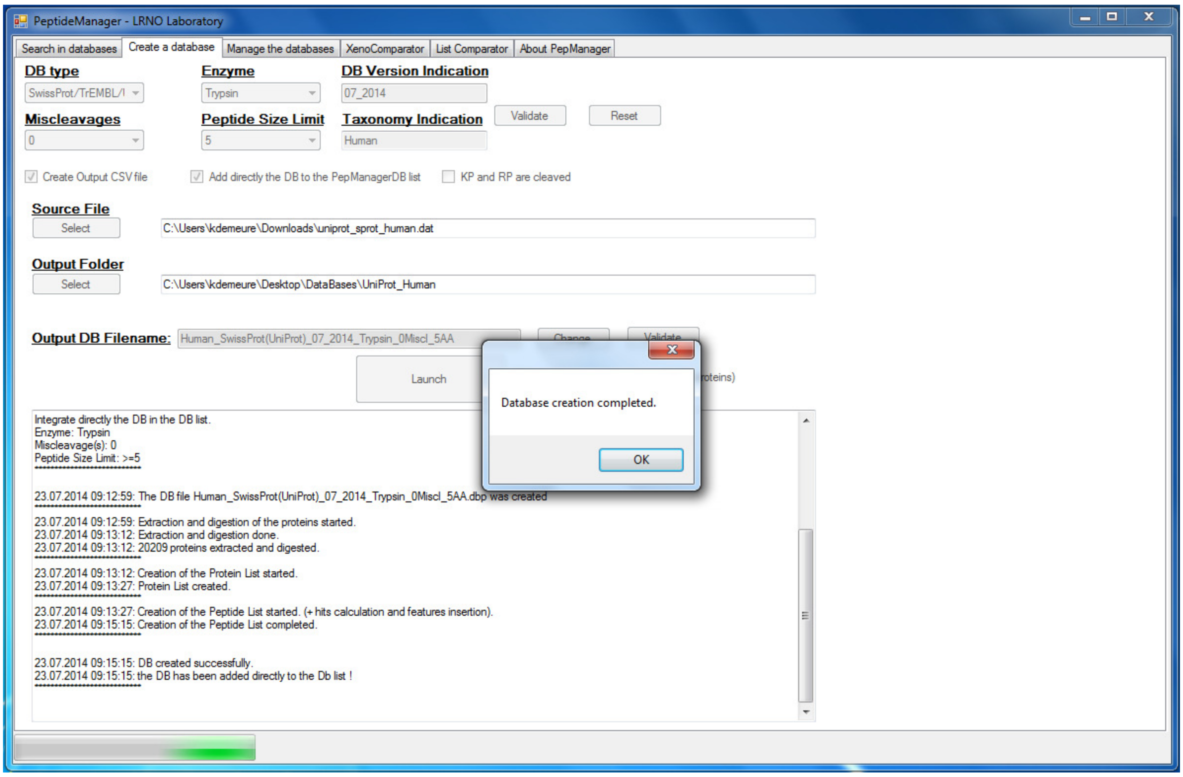

Figure 9.

When the creation of the database is done, a window as shown on Figure 9 should appear. If the "*Add directly the DB to the PeptideManager DB list*" checkbox was checked, the database is immediately available (Figure 10) for search requests (see Section "*Launch a search request*" and "*Select unique peptide sequences with the presence of a host/background proteome*" on Pages 13 and 26, respectively). If not, the database can be inserted later on via the "*Manage the databases*" tab (see Section "*Manage the PeptideManager databases*" on Page 9).

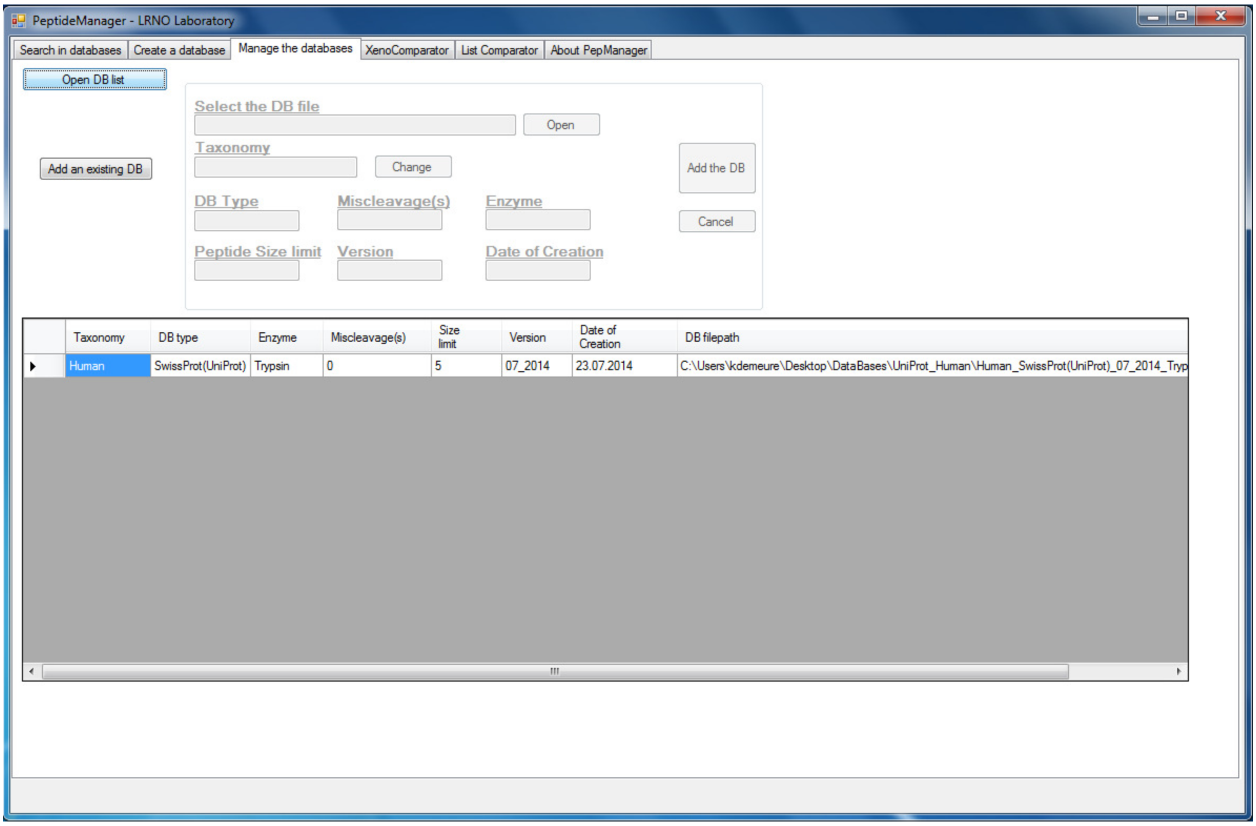

Figure 10.

Manage the PeptideManager databases

The "*Manage the databases*" tab (Figure 11) can be used to import already built PeptideManager databases and to access/modify some information about them. By clicking on "**Open DB list**", you access the list of peptide databases available in PeptideManager. At the first launch of PeptideManager, the list is empty (Figure 11).

To import an existing PeptideManager database, click on the "**Add an existing DB**" button (Figure 12). Indicate the path of the .dbp file of the PeptideManager database you want to import and click on "**Open**". Some information about the selected database is displayed. If wanted, you can modify the version and the taxonomy fields by clicking on "**Change**" and on "**Validate**" to confirm any changes.

Finally, click on "**Add the DB**" to finish the import of the database (Figure 13).

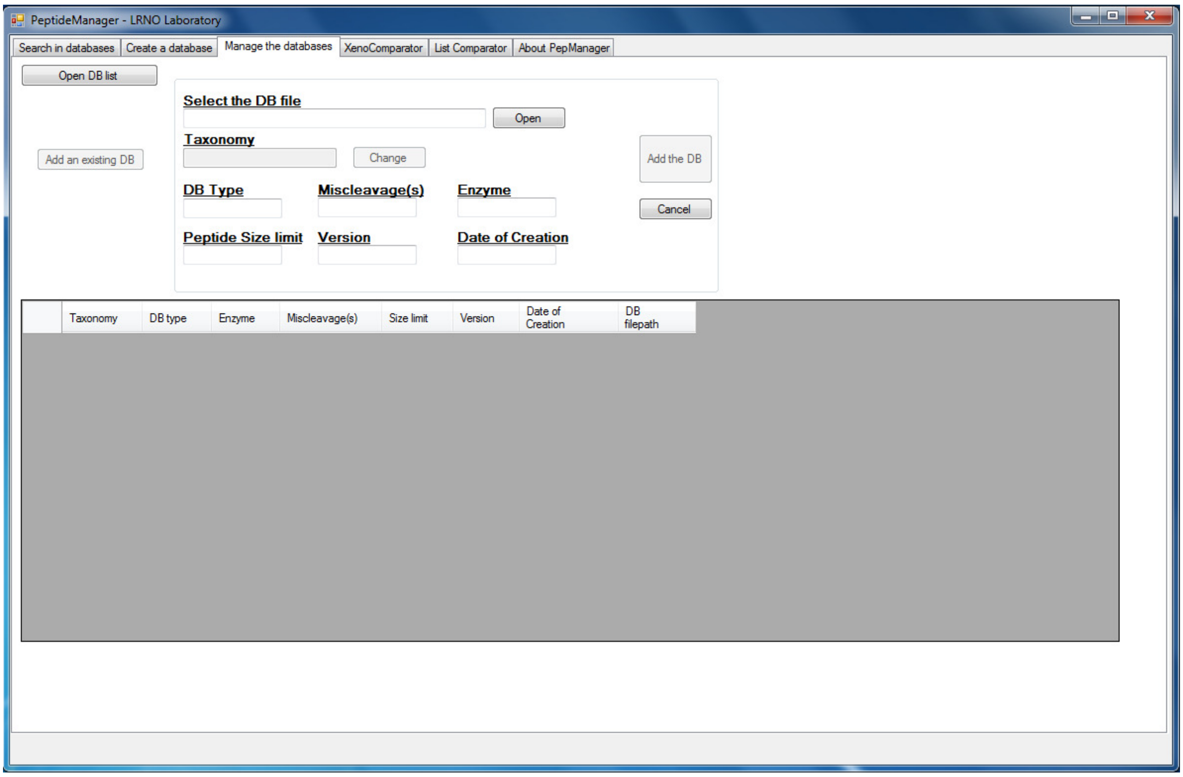

Figure 11.

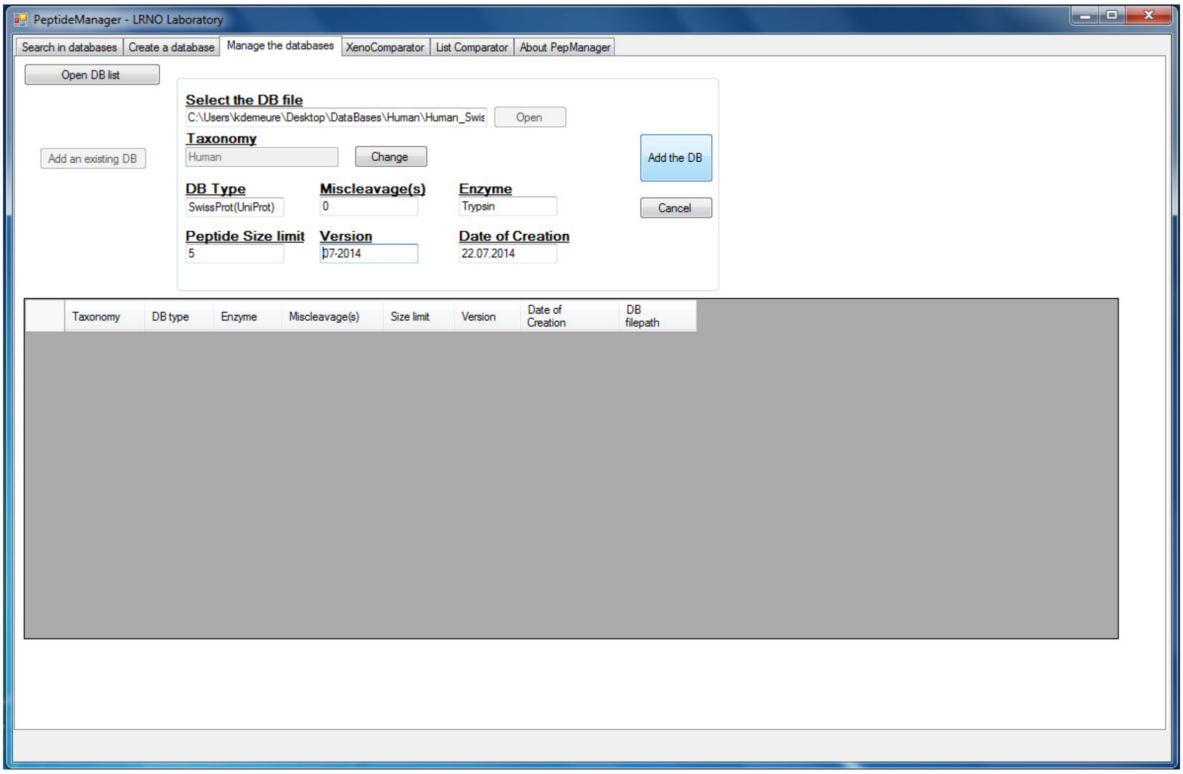

Figure 12.

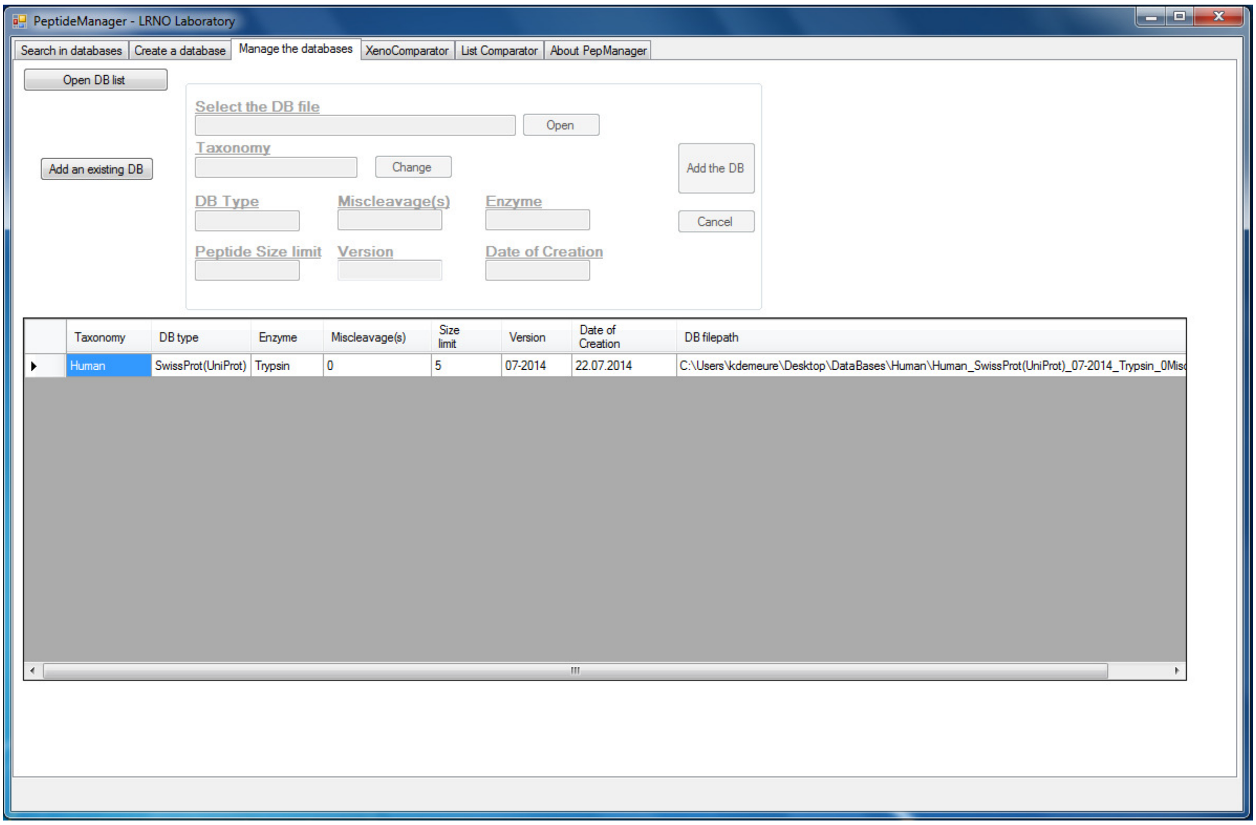

Figure 13.

To modify or delete a database from your PeptideManager databases list, click on the left side of the row containing the database to modify/delete to highlight it as shown on Figure 14 and to access the "**Delete the DB from the list**" and "**Modify the DB**" buttons. By clicking on the "**Delete the DB from the list**", you will remove the database from the list of available databases in PeptideManager. The files of the database are not erased, you can re-import the database later on if needed. As shown on Figure 15, by clicking the "**Modify the DB**" button, you can access the information about the database and modify the "*Taxonomy*" and "*Version*" fields.

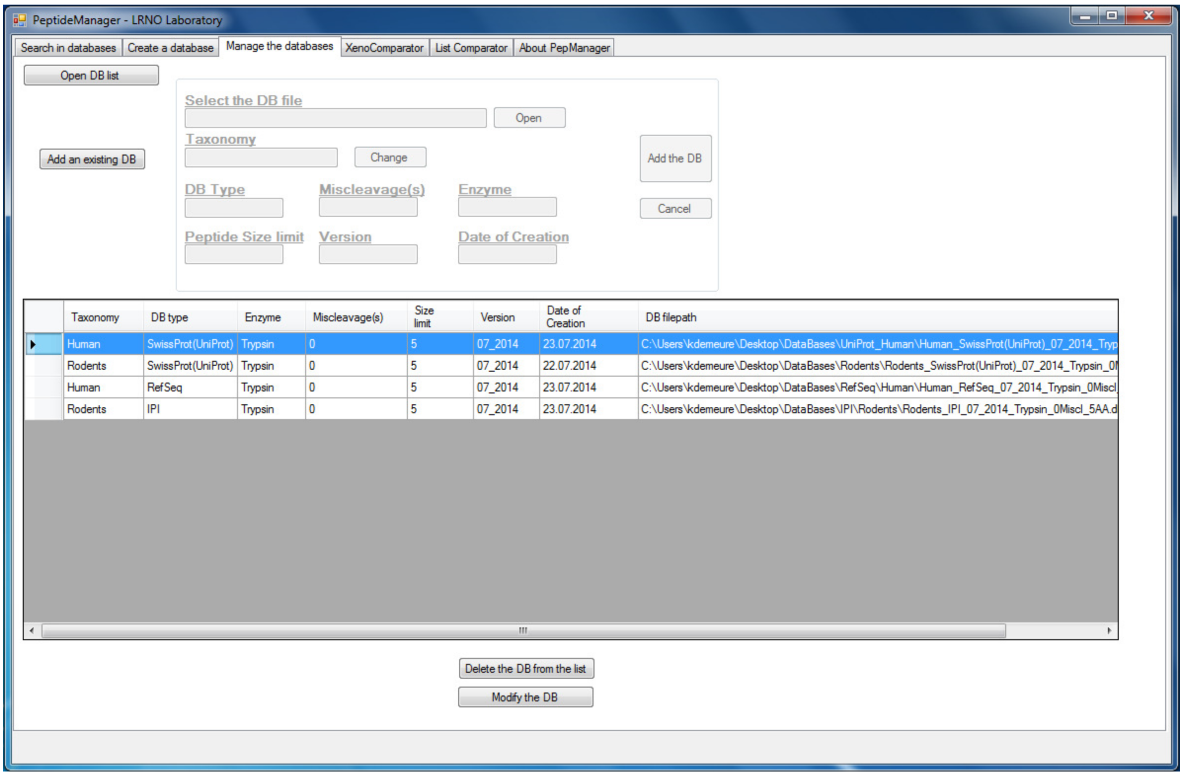

Figure 14.

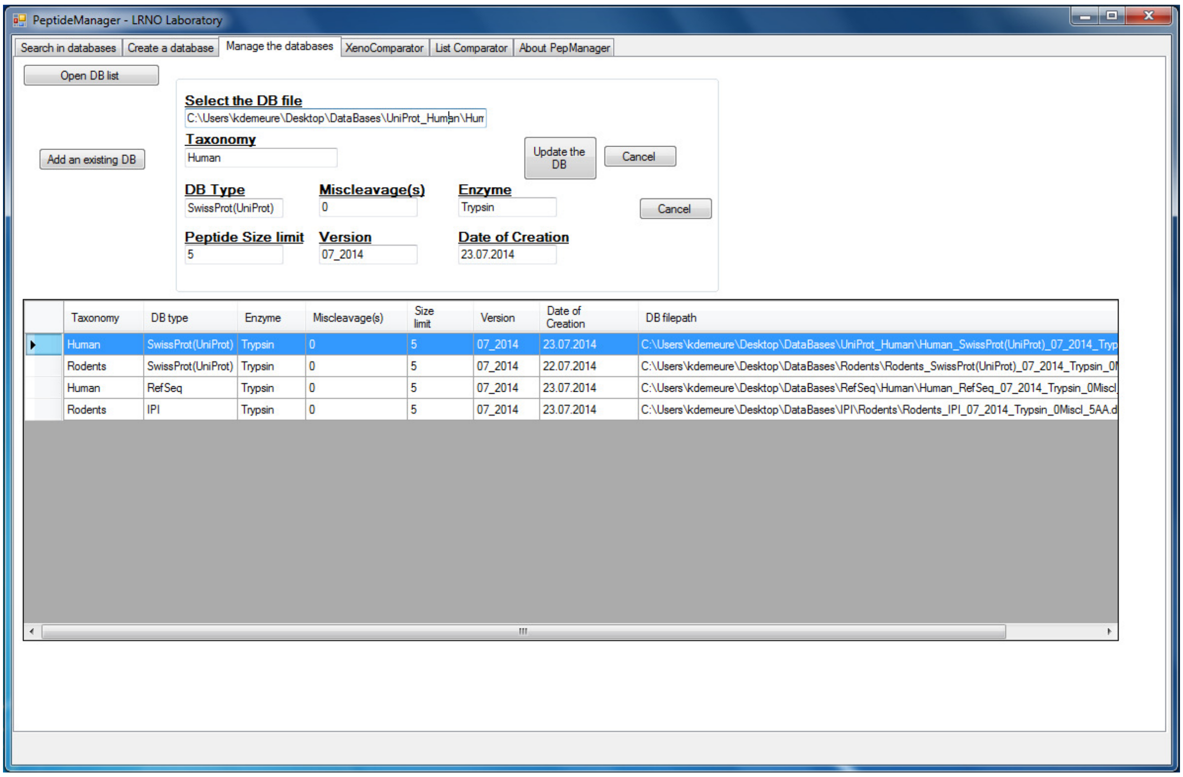

Figure 15.

Launch a search request

To perform a search request within your PeptideManager databases, select the "*Search in databases*" tab (Figure 16). If you want to perform a selection of unique peptides with the presence of a background/host proteome, please refer to Section "*Select unique peptide sequences with the presence of a host/background proteome*" on Page 26.

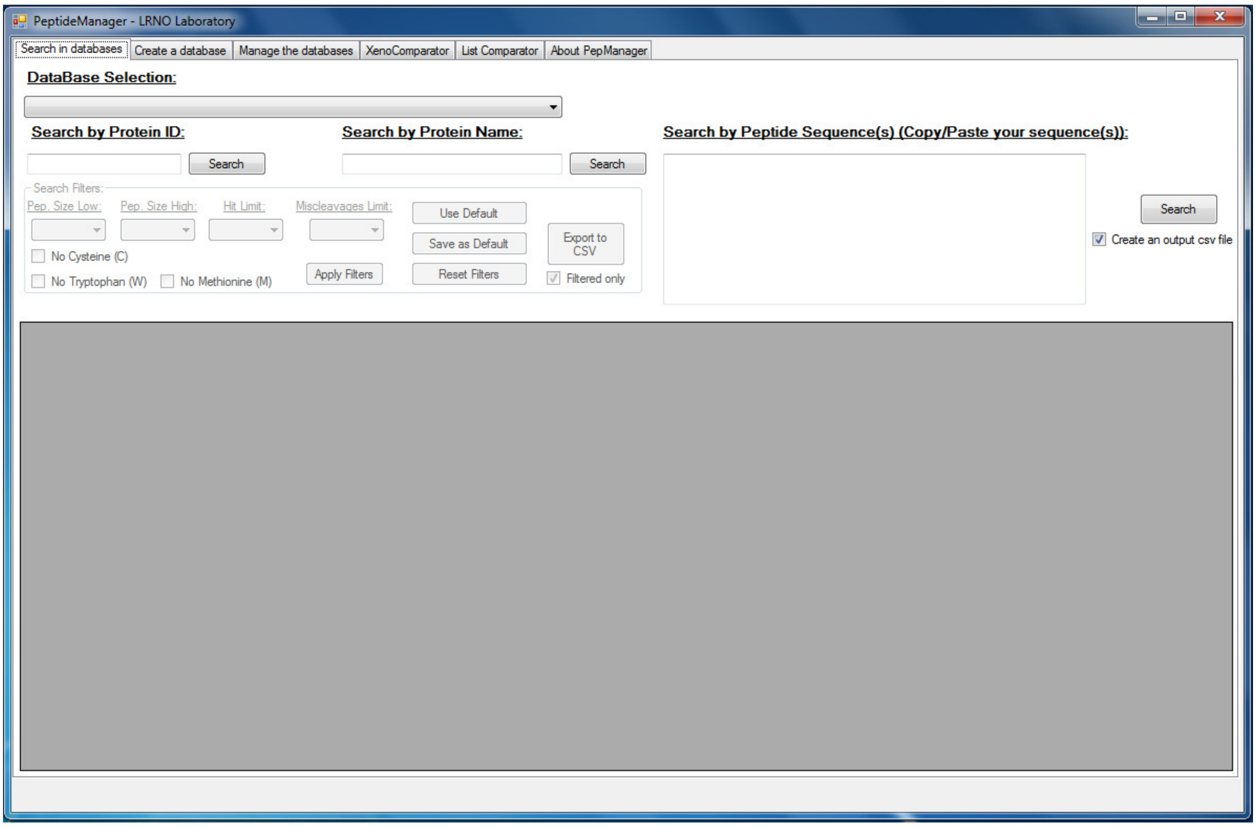

Figure 16.

You can perform a search request within a database by the protein ID, by the protein name or by peptide sequence(s).

Search by Protein ID

First, select the database in which you want to do your search request. Enter the protein ID and click on the corresponding "**Search**" button (Figure 16).

A window prompting you to choose the use or not of default filters should appear (Figure 17). Default filters are as follows: peptide size low limit of 5 amino acids, peptide size high limit of 22 amino acids, hit limit of 1, no miscleavages, no cysteine residues, no methionine residues, no tryptophan residues. Use default filters if you want to select unique peptide for targeted proteomics experiments. Click on "**Yes**" if

you want to use the default filters (Figure 18). Click on “**No**” if you want the unfiltered list of peptide sequences related to the protein (Figure 19).

The numbers of peptide sequences displayed (depending on the filters used) as well as the total number of peptide sequences for the given protein are indicated at the bottom of the window (Figure 18). The name of the protein is indicated above the data grid (Figure 18).

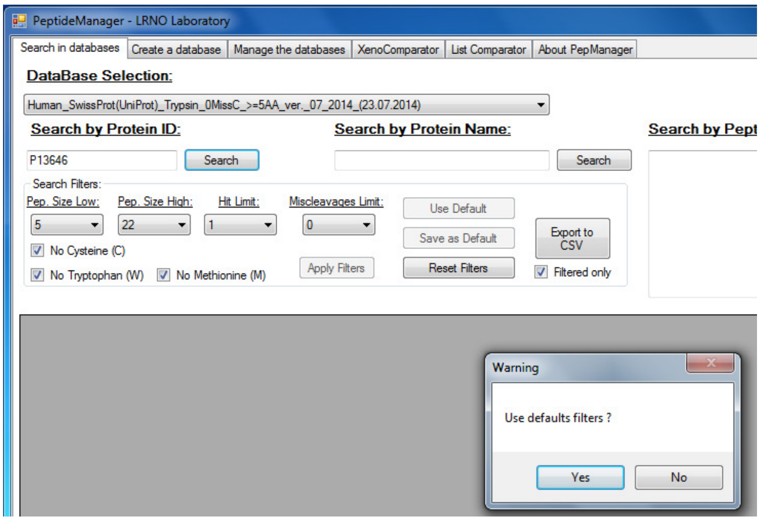

Figure 17.

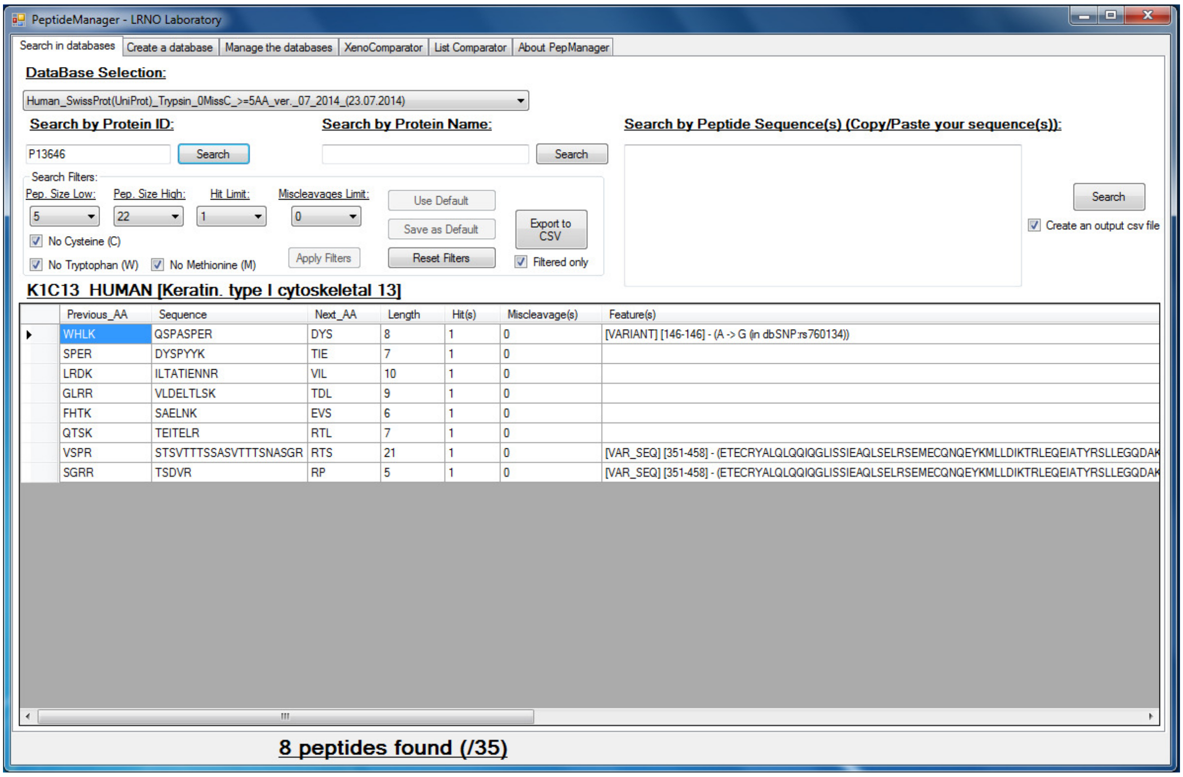

Figure 18.

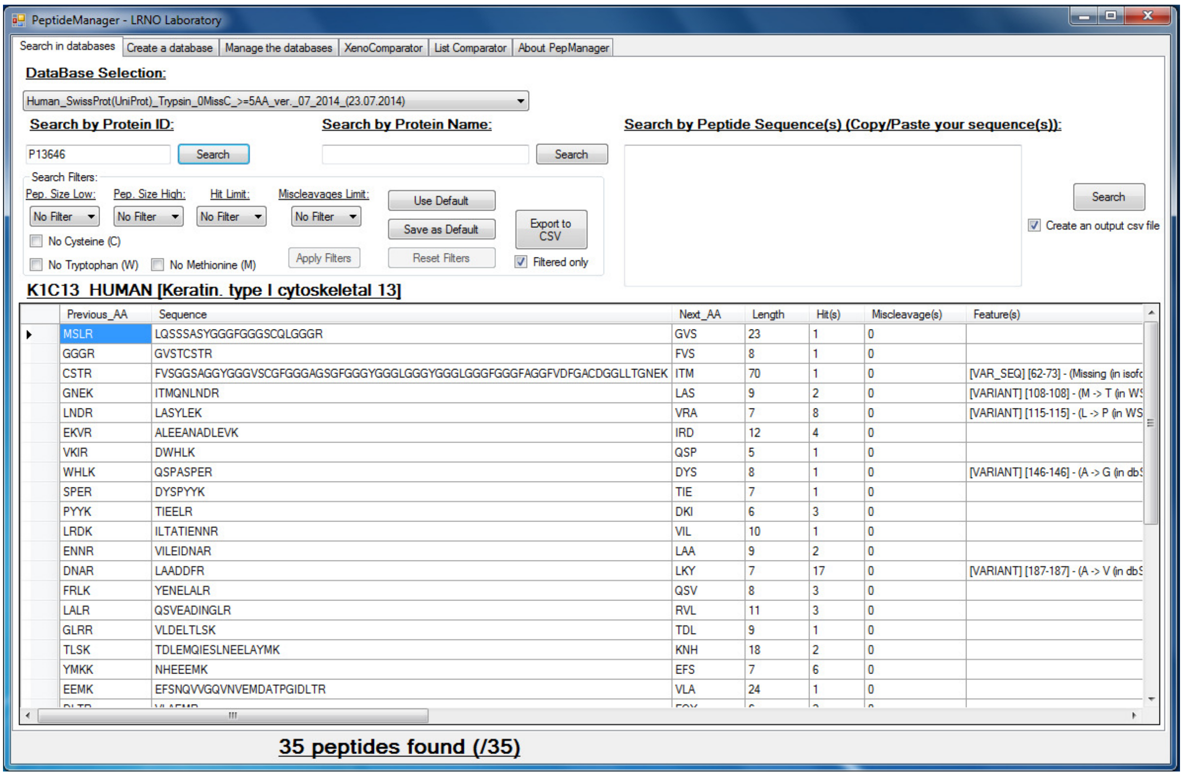

Figure 19.

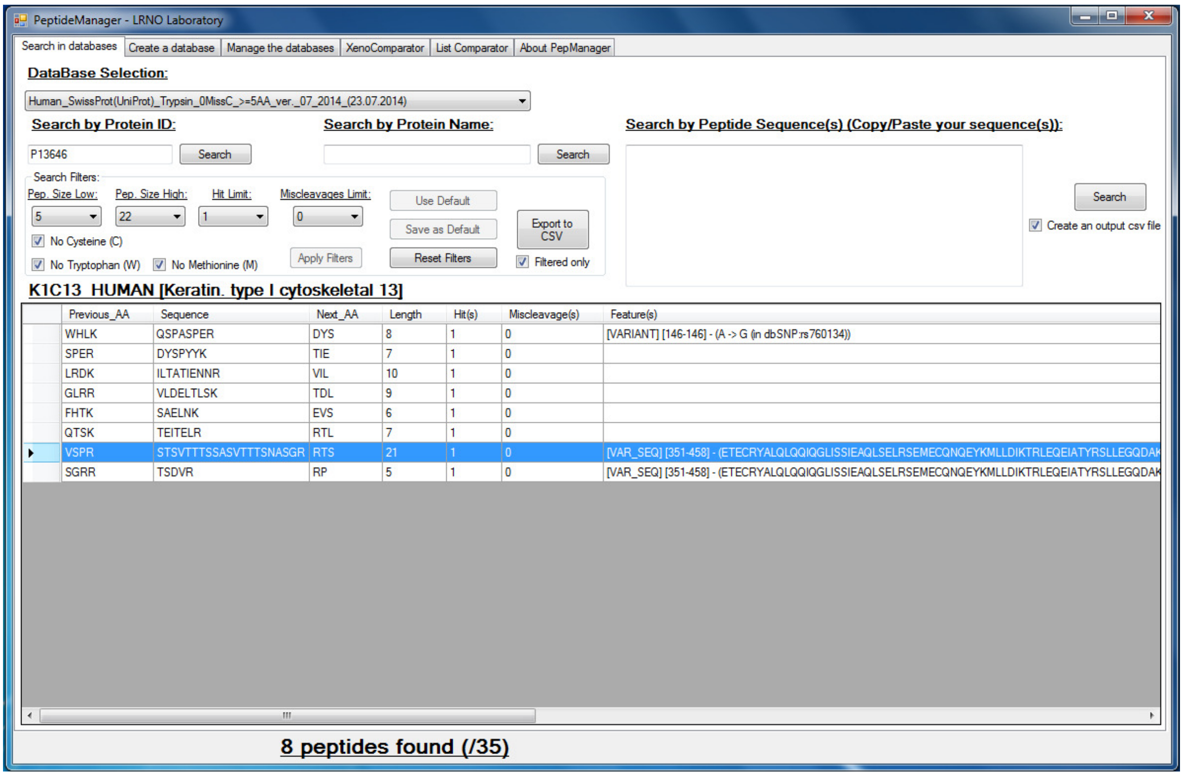

Figure 20.

You can modify any of the filters. Click on “**Apply Filters**” to apply the changes made to the filters and display the results in the data grid.

You can modify the values of the default filters. Modify the filters as whished and click on “**Save as Default**”.

Click on “**Reset Filters**” to reset to zero the filters (not the default values). Click on “**Apply Filters**” to obtain the unfiltered list of peptide sequences related to the protein.

Click on “**Use Default**” to set the default values of the different filters. Click on “**Apply Filters**” to obtain the filtered list of peptide sequences related to the protein with the default filters.

You have the possibility to delete any row of the data grid. Just click on the left side of the row (Figure 20) to highlight the row to delete and then press the delete touch of your keyboard to remove the row (corresponding to a peptide sequence) from the list (Figure 21).

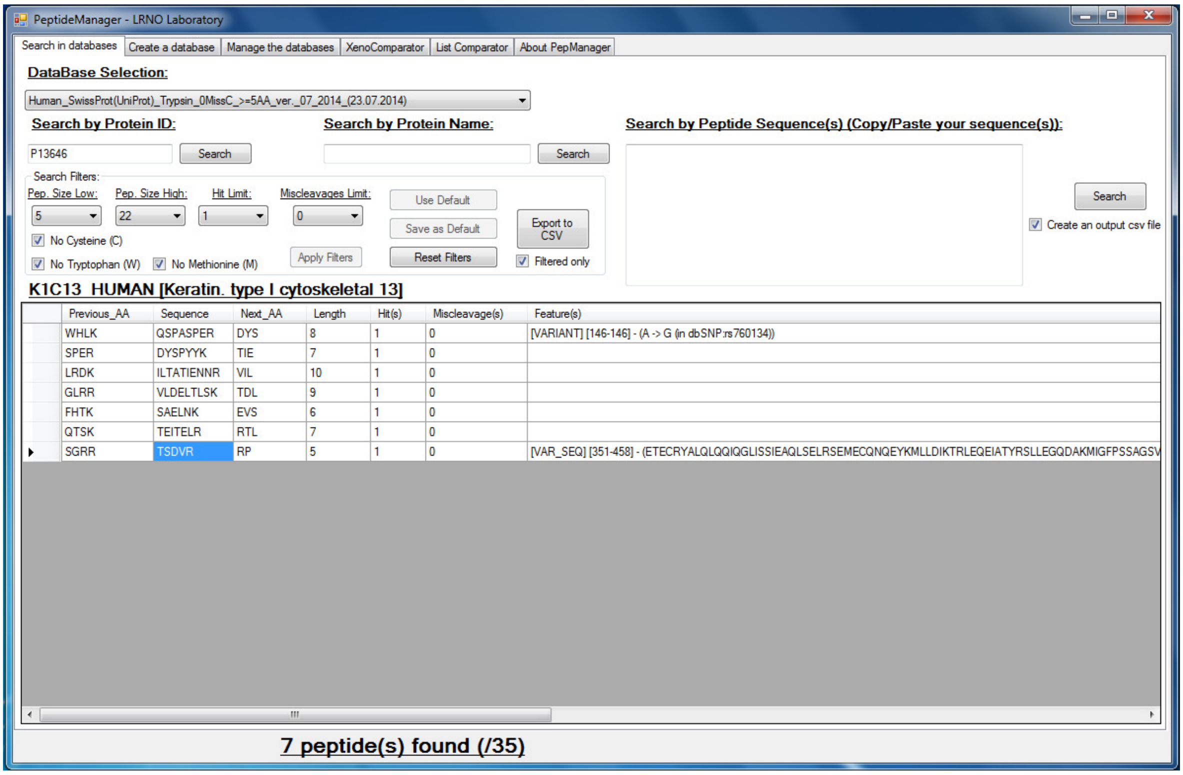

Figure 21.

To export the list of peptide sequences related to the given protein in CSV format, click on “**Export to CSV**”. If you want to only export the filtered results as they appear in the data grid (Figure 22), let the checkbox “*Filtered only*” in the checked status (Figure 21). By unchecking this checkbox, you will export the unfiltered results; *i.e.*, all the peptide sequences related to the given protein (Figure 23).

Previous_AA	Sequence	Next_AA	Length	Hit(s)	Miscleavage(s)	Feature(s)	MW	Start	Prot_Length	Accession
WHLK	QSPASPER	DYS	8	1	0	[VARIANT] [146-146] - (A -> G (in dbSNP:rs760134))	871.4274	143	458	P13646
SPER	DYSPYYK	TIE	7	1	0		935.4151	151	458	P13646
LRDK	ILTATIENNR	VIL	10	1	0		1144.6326	166	458	P13646
GLRR	VLDELTLSK	TDL	9	1	0		1017.5832	214	458	P13646
FHTK	SAELNK	EVS	6	1	0		661.3521	300	458	P13646
QTSK	TEITELR	RTL	7	1	0		861.4682	319	458	P13646
SGRR	TSDVR	RP	5	1	0	[VAR_SEQ] [351-458] - (ETECRYALQLQQIQGLISSIEAQLSELRSE	577.2946	452	458	P13646

P13646_human

Figure 22.

Previous_AA	Sequence	Next_AA	Length	Hit(s)	Miscleavage(s)	Feature(s)	MW	Start	Prot_Length	Accession
MSLR	LQSSSASYGGGFGGGSCQLGGGR	GVS	23	1	0		2089.9361	5	458	P13646
GGGR	GVSTCSTR	FVS	8	1	0		810.378	28	458	P13646
CSTR	FVSGGSAGGYGGGVSCGFGGGAGSGFGGGYGGGLGG	ITM	70	1	0	[VAR_SEQ] [62-73] - (Missing (in	5937.5999	36	458	P13646
GNEK	ITMQNLNDR	LAS	9	2	0	[VARIANT] [108-108] - (M -> T (in	1104.5472	106	458	P13646
LNDR	LASYLEK	VRA	7	8	0	[VARIANT] [115-115] - (L -> P (in	823.4565	115	458	P13646
EKVR	ALEEANADLEVK	IRD	12	4	0		1301.6589	124	458	P13646
VKIR	DWHLK	QSP	5	1	0		698.3626	138	458	P13646
WHLK	QSPASPER	DYS	8	1	0	[VARIANT] [146-146] - (A -> G (in	871.4274	143	458	P13646
SPER	DYSPYYK	TIE	7	1	0		935.4151	151	458	P13646
PYYK	TIEELR	DKI	6	3	0		760.4205	158	458	P13646
LRDK	ILTATIENNR	VIL	10	1	0		1144.6326	166	458	P13646
ENNR	VILEIDNAR	LAA	9	2	0		1042.5897	176	458	P13646
DNAR	LAADDFR	LKY	7	17	0	[VARIANT] [187-187] - (A -> V (in	807.4001	185	458	P13646
FRLK	YENELALR	QSV	8	3	0		1007.5162	194	458	P13646
LALR	QSVEADINGLR	RVL	11	3	0		1201.6177	202	458	P13646
GLRR	VLDELTLSK	TDL	9	1	0		1017.5832	214	458	P13646
TLSK	TDLEMQIESLNEELAYMK	KNH	18	2	0		2157.0095	223	458	P13646
YMKK	NHEEEMK	EFS	7	6	0		916.3835	242	458	P13646
EEMK	EFSNQVVGQVNVEMDATPGIDLTR	VLA	24	1	0		2619.2725	249	458	P13646
DLTR	VLAEMR	EQY	6	2	0		718.3922	273	458	P13646
AEMR	EQYEAMAER	NRR	9	1	0		1126.4839	279	458	P13646
RNRR	DAEEWFHTK	SAE	9	1	0	[VARIANT] [298-298] - (T -> A (in	1162.5169	291	458	P13646
FHTK	SAELNK	EVS	6	1	0		661.3521	300	458	P13646
ELNK	EVSTNTAMIQTSK	TEI	13	1	0		1409.6946	306	458	P13646
QTSK	TEITELR	RTL	7	1	0		861.4682	319	458	P13646
ELRR	TLQGLEIELQSQLSMK	AGL	16	2	0		1817.9683	327	458	P13646

P13646_human_notFiltered

Figure 23.

Search by Protein Name

After having selected the database to use, type the protein name or a part of it in the "*Search by Protein Name*" textbox (Figure 24) and click on the corresponding "**Search**" button.

The results are displayed in the data grid and the number of proteins found is indicated at the bottom of the data grid (Figure 25).

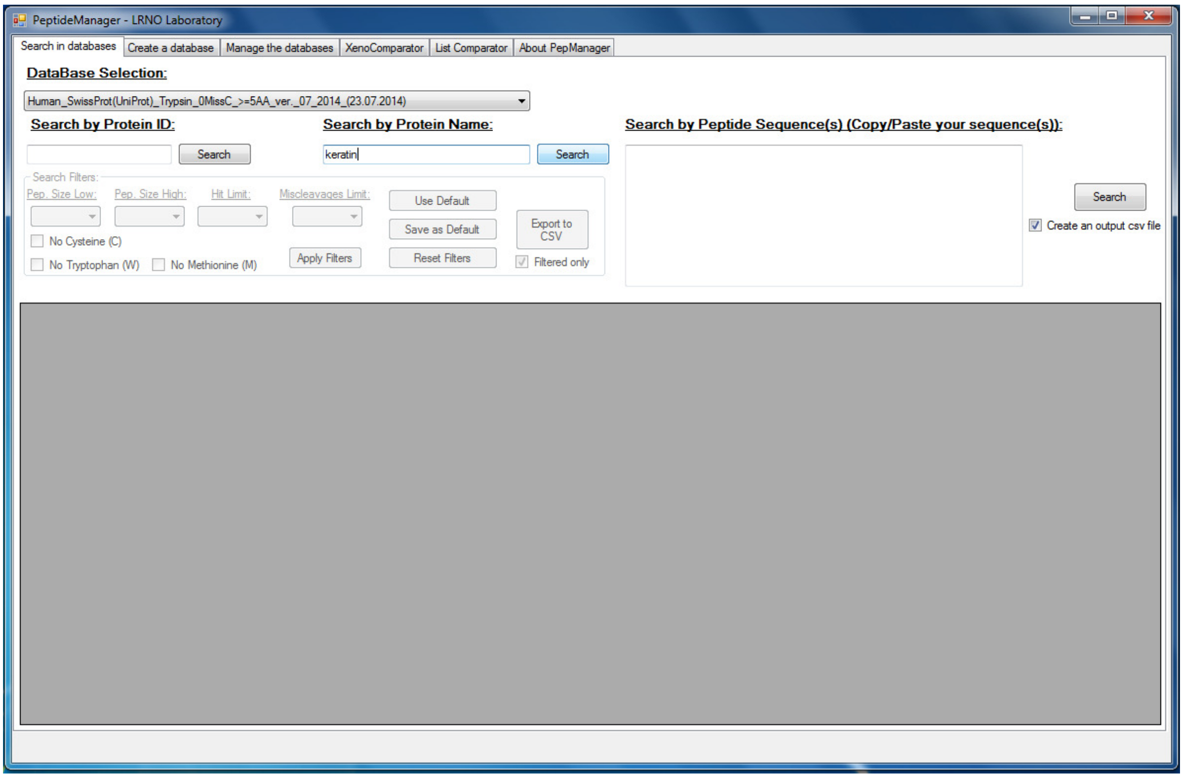

Figure 24.

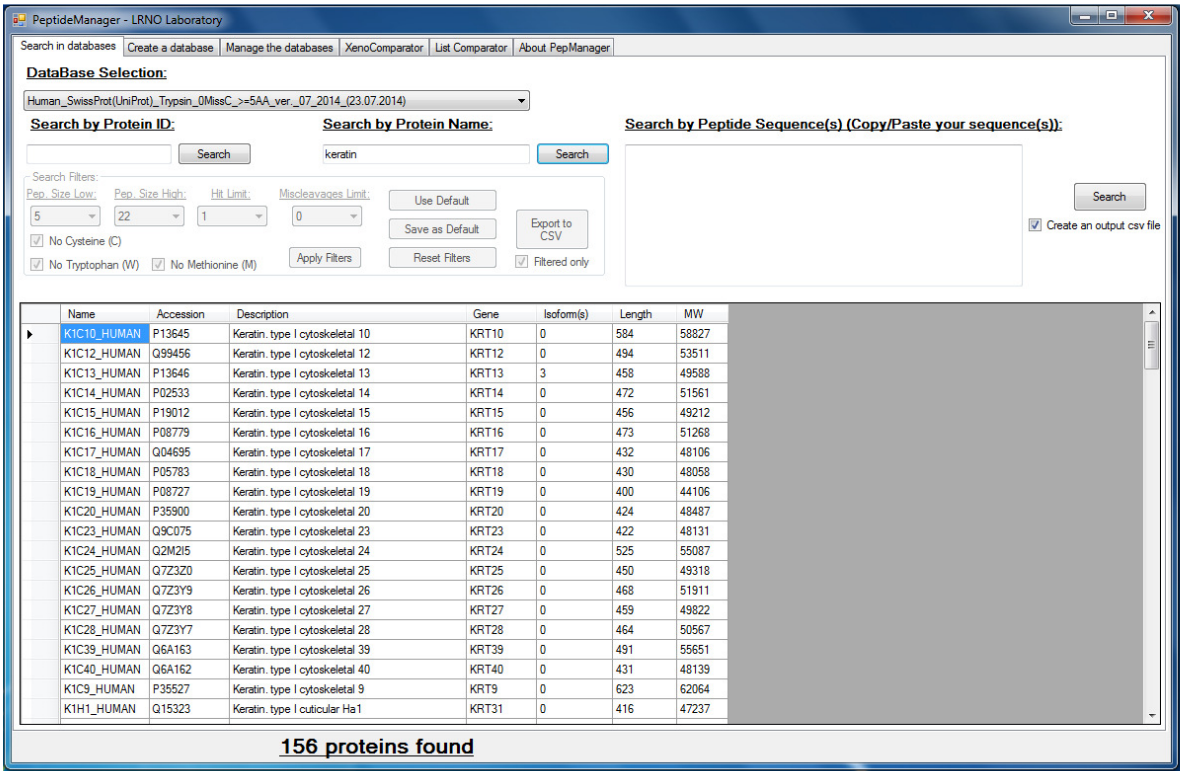

Figure 25.

Search by Peptide Sequence(s)

In order to get the number of hits/occurrence of a peptide sequence or a list of peptide sequences within a database, use the “*Search by Peptide Sequence(s)*” part of the “*Search in databases*” tab (Figure 26).

Select the database of interest and type or copy/paste your peptide sequence or your list of peptide sequences in the corresponding textbox (Figure 26). If you want to export the results as csv file let the checkbox “*Create an output csv file*” in the checked status and you will be asked to choose the output file name and directory. To get the results, click on the corresponding “**Search**” button and the results will be displayed in the data grid (Figure 26).

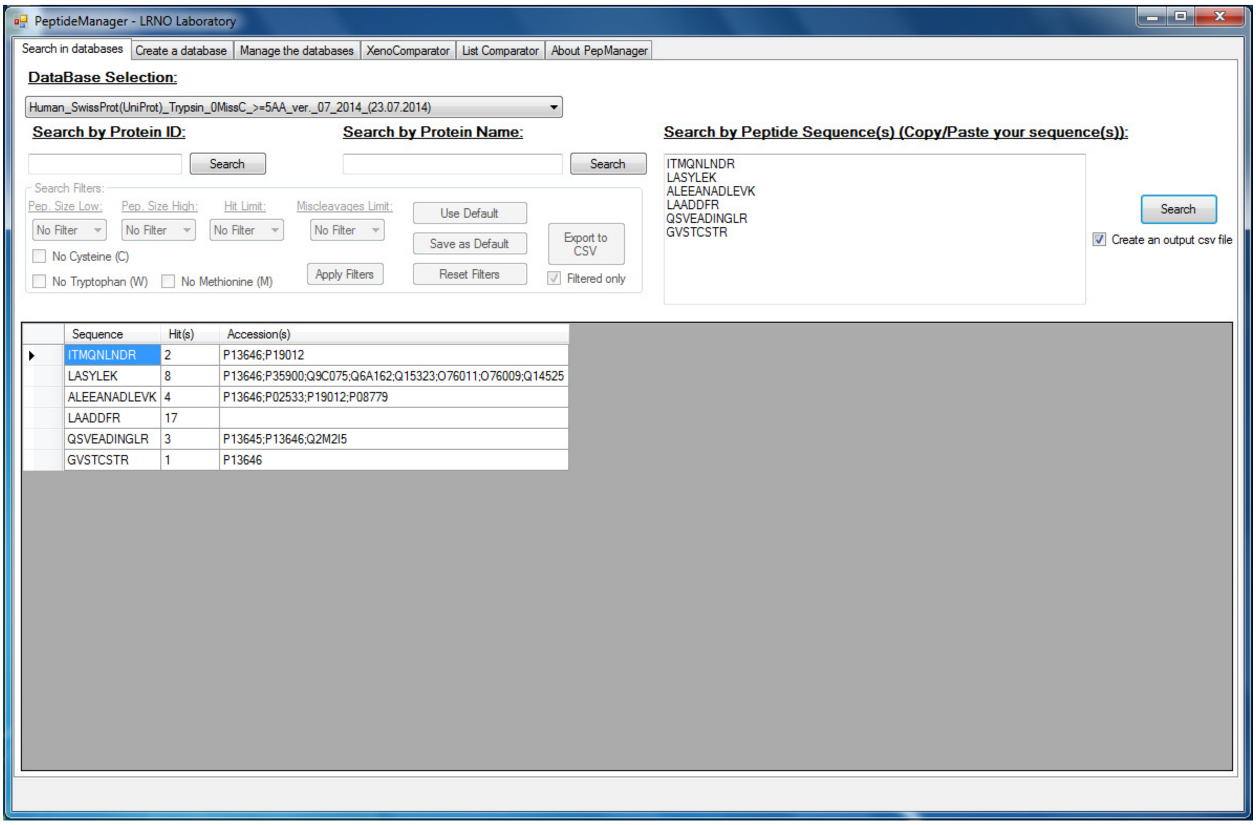

Figure 26.

Case Studies: Select unique peptide sequences for a targeted proteomics experiment

Unique peptide sequences selection – P12352

In this case study, the P12352 protein (Creatine Kinase U-Type, mitochondrial) is taken as an example. The database used is the human database of SwissProt with only the reviewed proteins (07_2014) (see Section "*Download the public protein databases*" on Page 38 for further information on how to download databases from public data repository and see Section "*Build a peptide database*" on Page 5 to learn how to create a PeptideManager database from protein databases found in the public data repositories). In the "*Search in databases*" tab, type "P12352" in the "*Search by Protein ID*" textbox and click on the "**Search**" button. Accept the use of the default filters and you should obtain similar results as those shown on Figure 27; 10 filtered peptides on a total of 31.

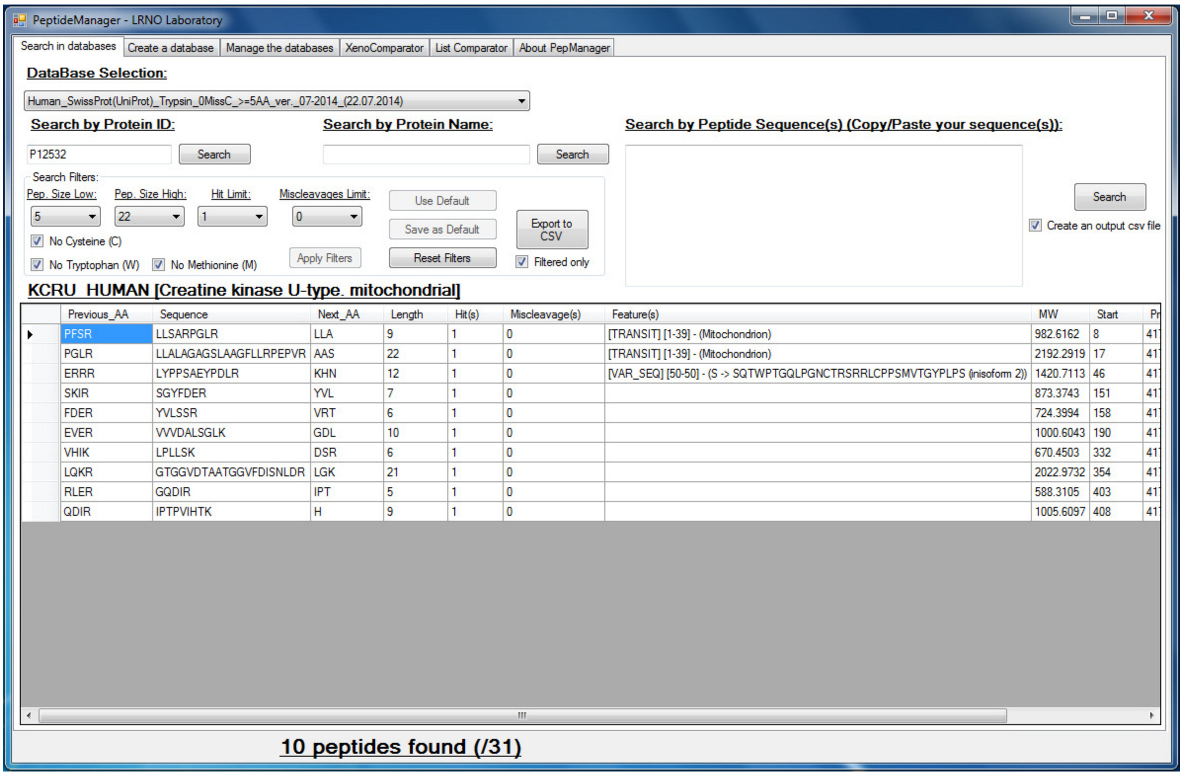

Figure 27.

As indicated in the "*Features column*", the first two peptide sequences of the list (Figure 28) are part of the "Transit peptide" part of the protein and are therefore discarded (Figure 29) since transit peptides are generally cleaved from the mature protein.

If all the isoforms of the protein (there are two isoforms of the protein) should be monitored/quantified, the first peptide sequence of the list (Figure 29) should also be discarded as its sequence differs from one isoform to the other (Figure 30).

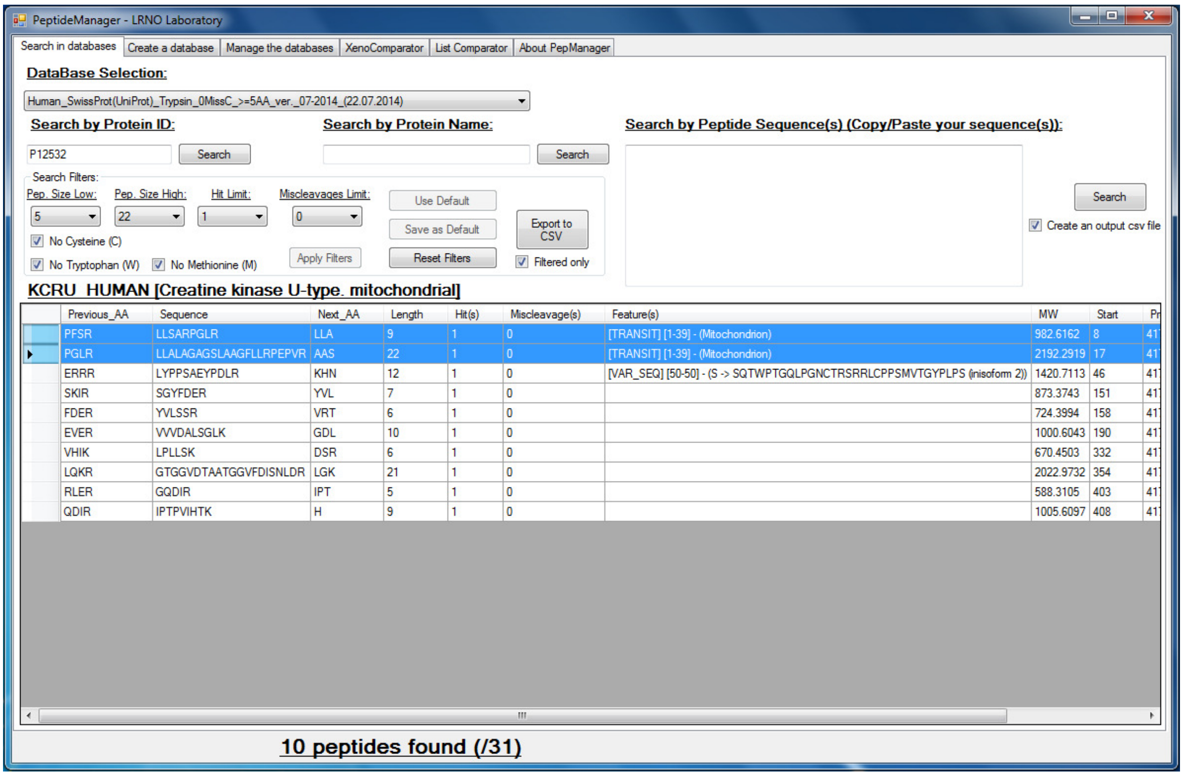

Figure 28.

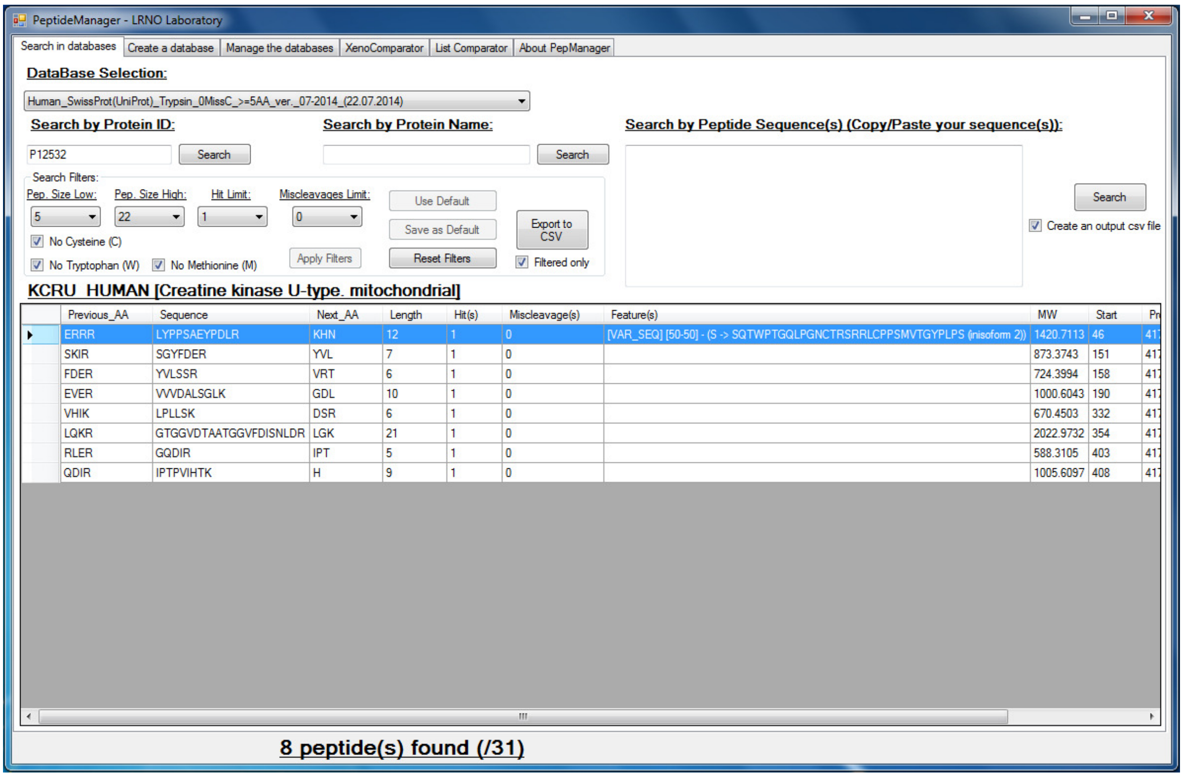

Figure 29.

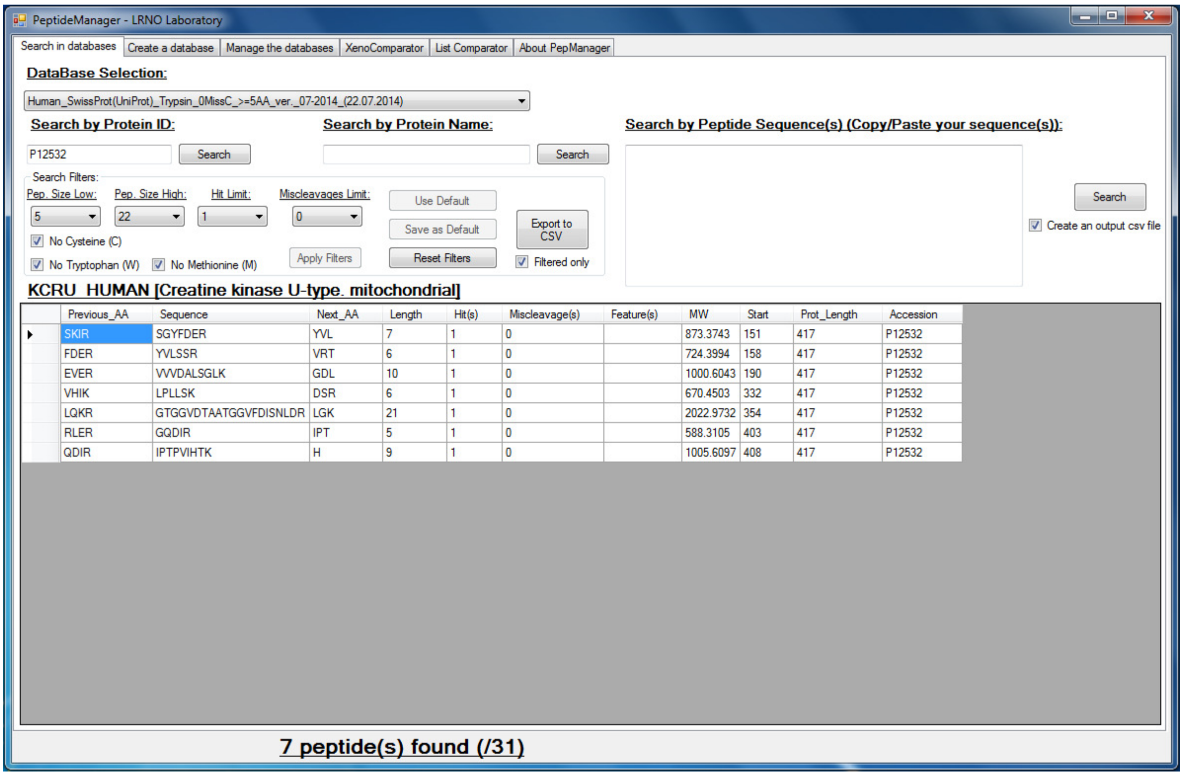

Figure 30.

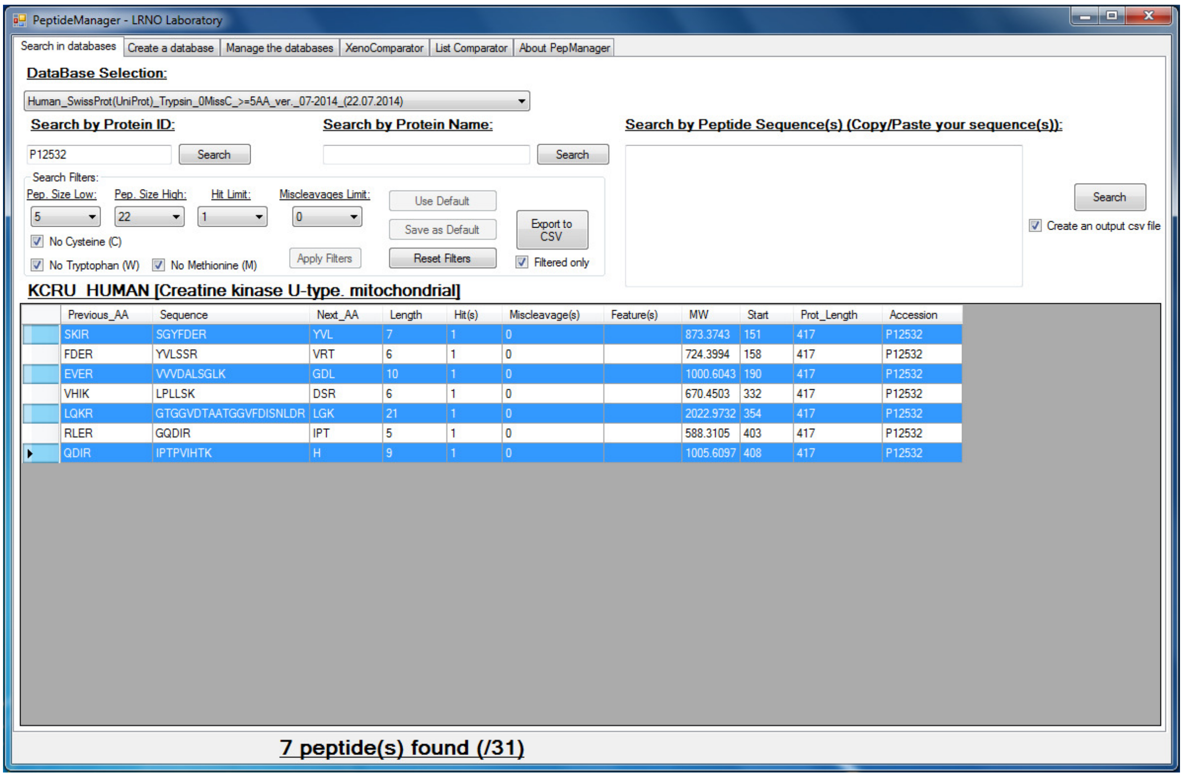

Figure 31.

In the end, a total of 7 unique peptide sequences were selected in order to target the P12532 protein. If we had to limit the number of peptide candidates, the four peptide sequences highlighted in Figure 31 are the best candidates since they are the larger ones (decrease the risk of interferences) and are present at different position (start, middle, end) within the protein sequence (*i.e.*; 151/417, 190/417, 354/417 and 408/417 as indicated in the "*Start*" (this column indicates the position of the first amino acid position of the peptide sequence within the protein sequence) and "*Prot_Length*" columns) (Figure 32).

The selected peptide sequences for the P12352 protein can be saved in a csv file (Figure 33) by clicking on the "**Export to CSV**" button with the "*Filtered only*" checkbox in the checked status.

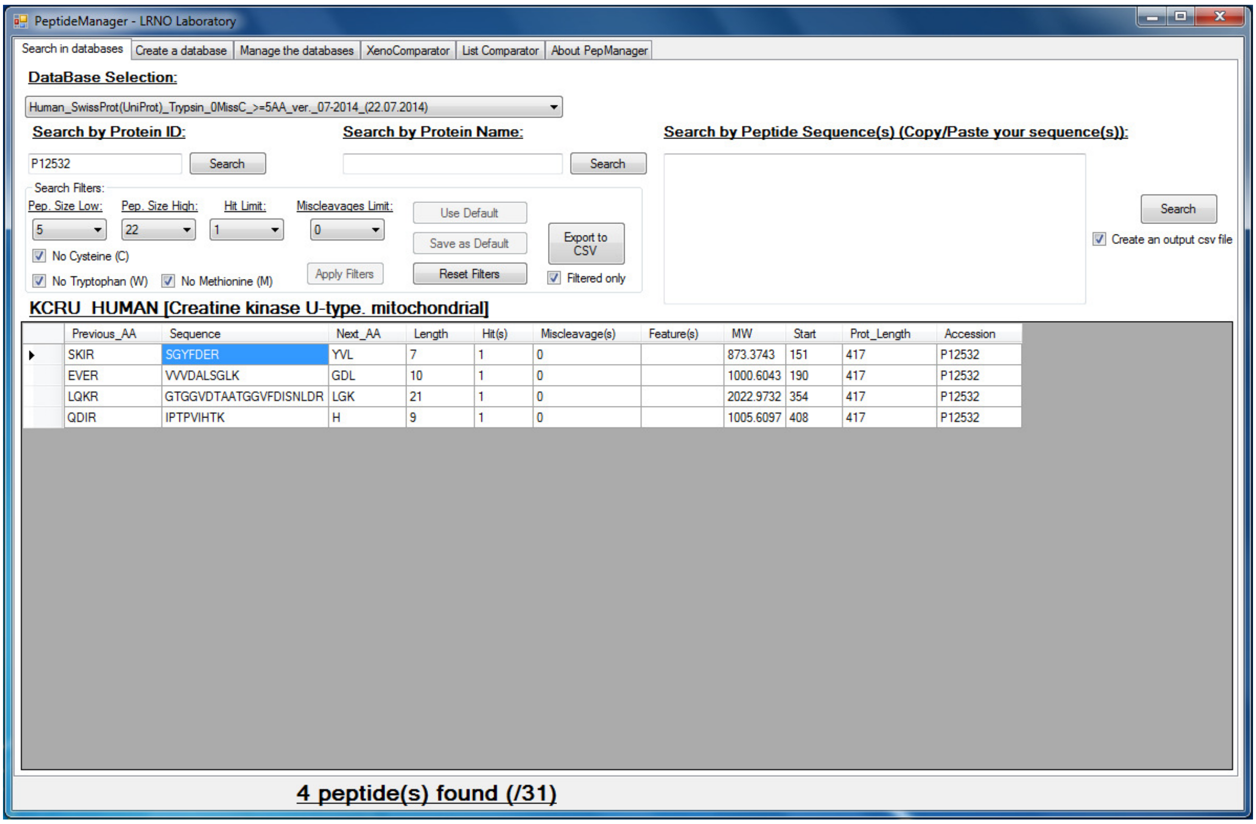

Figure 32.

	A	B	C	D	E	F	G	H	I	J	K
1	Previous_AA	Sequence	Next_AA	Length	Hit(s)	Miscleavage(s)	Feature(s)	MW	Start	Prot_Length	Accession
2	SKIR	SGYFDER	YVL	7	1	0		873.3743	151	417	P12532
3	EVER	VVVDALSGLK	GDL	10	1	0		1000.6043	190	417	P12532
4	LQKR	GTGGVDTAATGGVFDISNLDR	LGK	21	1	0		2022.9732	354	417	P12532
5	QDIR	IPTPVIHTK	H	9	1	0		1005.6097	408	417	P12532
6											

Figure 33.

Peptide selection to differentiate protein isoforms

From the previous example of the P12352 protein, if the aim was also to differentiate the two isoforms, only the peptide **LYPPSAEYPDLR** fulfill the requirements (Figures 34 and 35). This peptide is unique to the P12352 proteins and is only observed in the first isoforms since its sequence is **LYPPSQTWPTGQLPGNCTR/SRRLCPSMVTGYPLPSAEYPDLR** in the second isoform. To only monitor the second isoform, the **LYPPSQTWPTGQLPGNCTR** peptide is a good candidate since it is only representative of the second isoform (Figure 36) but it contains a tryptophan and a cysteine residues. Therefore, the four peptides from the previous example and the **LYPPSAEYPDLR** peptide are the peptide candidates appropriate to target/quantify both isoforms and only the first isoform, respectively. The proportion of the second isoforms will be deducible from these quantification data.

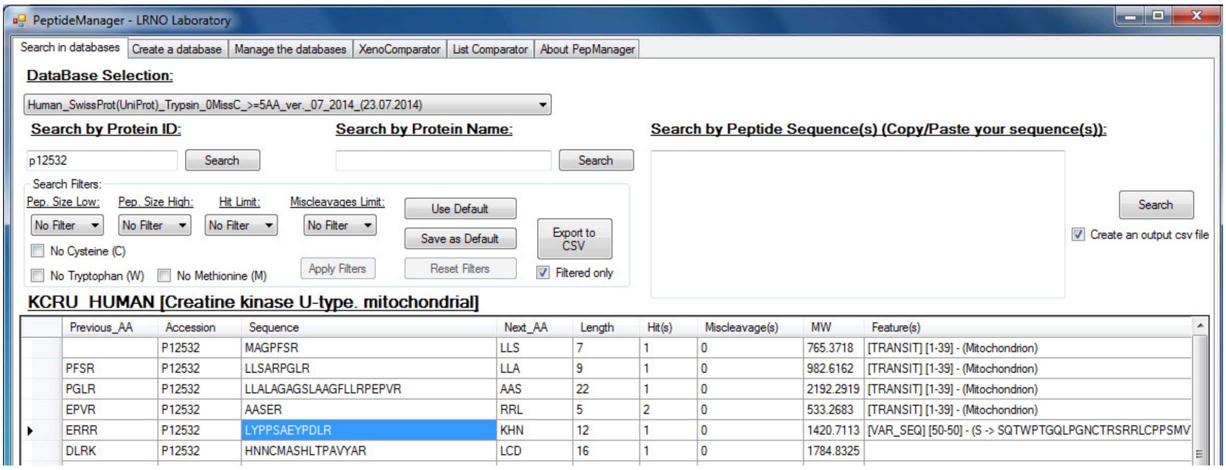

Figure 34.

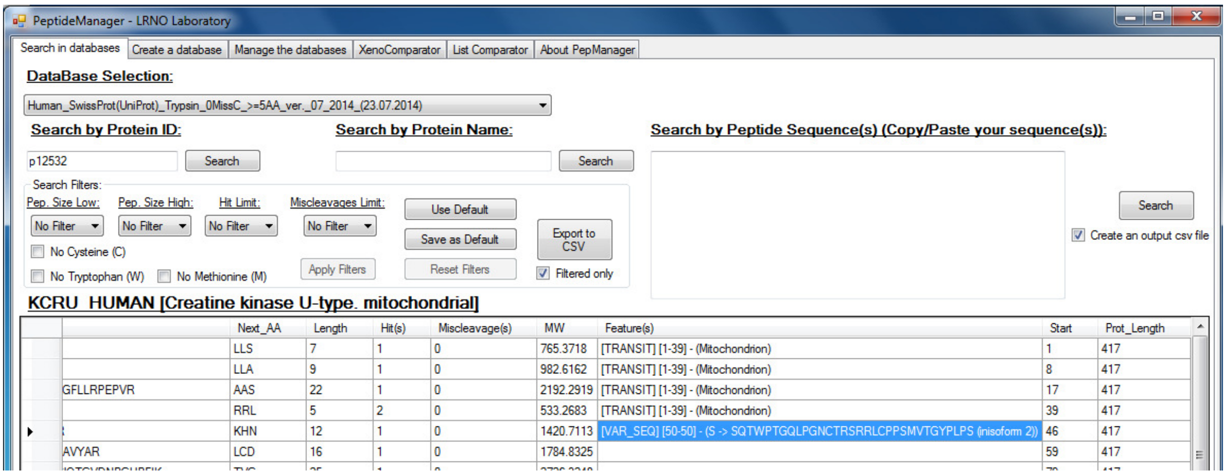

Figure 35.

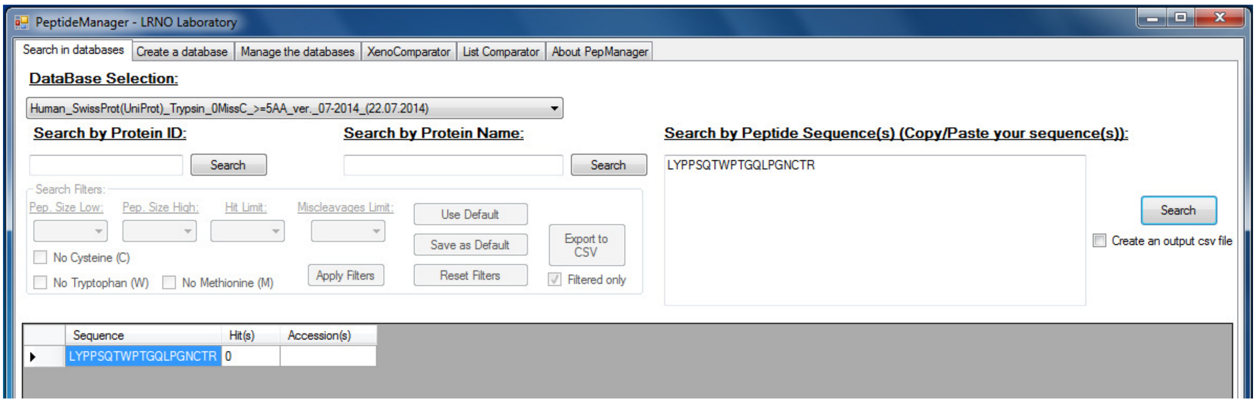

Figure 36.

Monitoring post-translational modifications of interest

Generally, peptides containing post-translational modifications are not wished since they introduce a bias for the quantification of the protein compared with peptides that do not contain any post-translational modification. However, in some cases, you may want to evaluate the relative proportion of different post-translational modifications of a same protein. For that purpose, you will have to select peptides containing the modifications of interest and monitor/quantify those peptides with and without the modifications of interest.

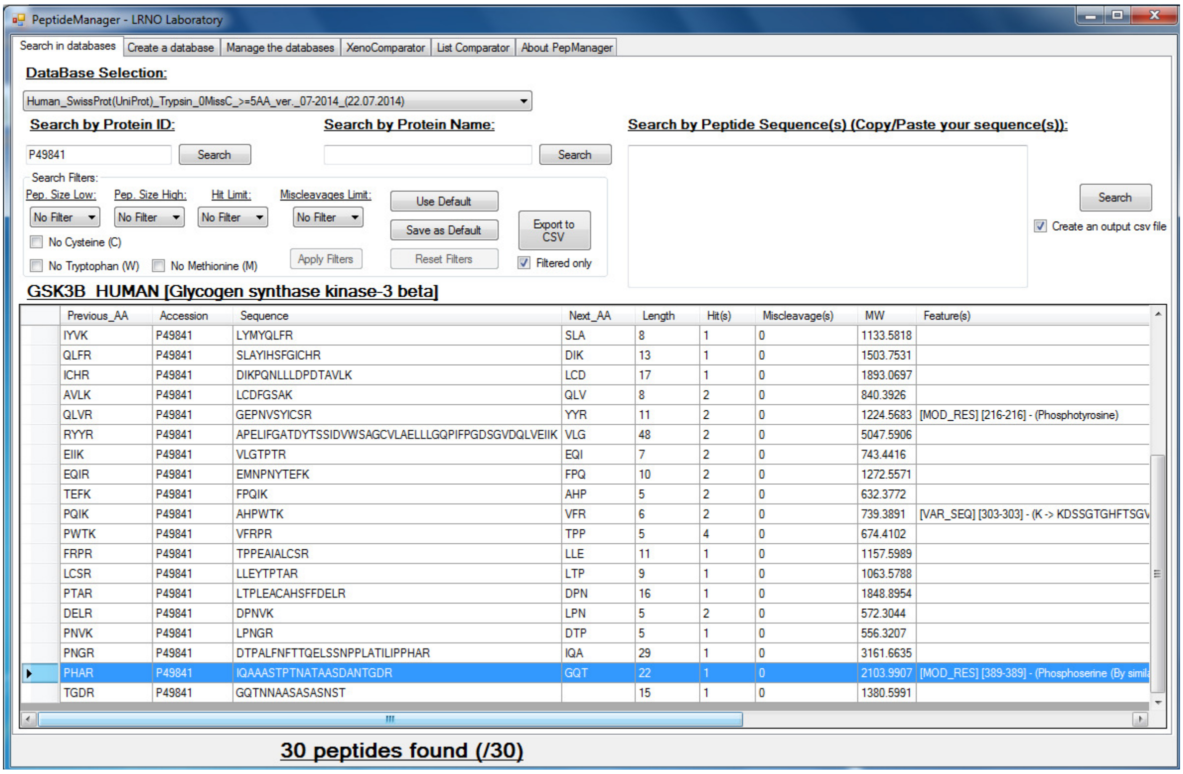

Figure 37.

With the P49841 protein (Glycogen synthase kinase-3 beta) as an example (purely fictional), we will target the phosphorylation status of this protein on the following amino acids: serine 389, serine 390 and threonine 402.

It is the peptide **IQAAASTPTNATAASDANTGDR** that contains all the modifications of interest as shown on Figures 37 and 38. In order to quantify the various modified forms, all the different combinations (2x2x2 = 8 possible combinations) of modified/unmodified phosphorylation sites have to be monitored/quantified.

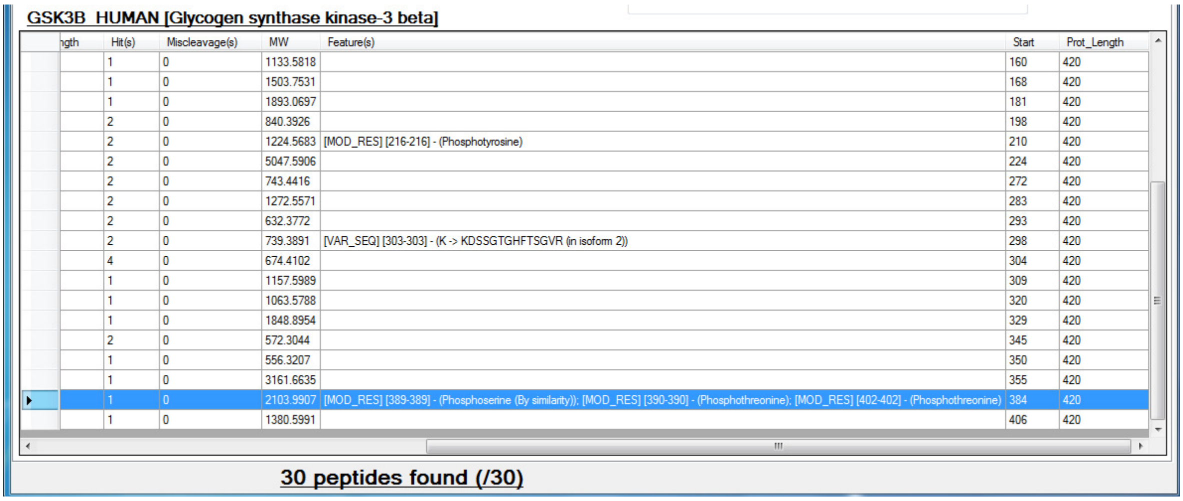

GSK3B_HUMAN [Glycogen synthase kinase-3 beta]

ngth	Hit(s)	Miscleavage(s)	MW	Feature(s)	Start	Prot_Length
	1	0	1133.5818		160	420
	1	0	1503.7531		168	420
	1	0	1893.0697		181	420
	2	0	840.3926		198	420
	2	0	1224.5683	[MOD_RES] [216-216] - (Phosphotyrosine)	210	420
	2	0	5047.5906		224	420
	2	0	743.4416		272	420
	2	0	1272.5571		283	420
	2	0	632.3772		293	420
	2	0	739.3891	[VAR_SEQ] [303-303] - (K -> KDSSGTGHFTSGVR (in isoform 2))	298	420
	4	0	674.4102		304	420
	1	0	1157.5989		309	420
	1	0	1063.5788		320	420
	1	0	1848.8954		329	420
	2	0	572.3044		345	420
	1	0	556.3207		350	420
	1	0	3161.6635		355	420
	1	0	2103.9907	[MOD_RES] [389-389] - (Phosphoserine (By similarity)); [MOD_RES] [390-390] - (Phosphothreonine); [MOD_RES] [402-402] - (Phosphothreonine)	384	420
	1	0	1380.5991		406	420

30 peptides found (/30)

Figure 38.

Select unique peptide sequences with the presence of a host/background proteome

With PeptideManager, it is possible to perform unique peptide selection with the presence of a background/host proteome. For a given protein, PeptideManager extracts the corresponding peptide sequences from the proteome of interest and assesses the occurrence of these peptide sequences within the background/host proteome. Indeed, to monitor specifically a protein from the proteome of interest without any bias, you have to monitor peptide that are unique representative of the targeted protein within the proteome of interest (hit(s) = 1) and that are not observed within the host/background proteome (hit(s) = 0).

It is possible to get these peptide sequences for one protein at a time via the “*filtering mode*” of the “*XenoComparator*” tab or for a list of proteins via the “*batch mode*”.

Filtering Mode

With the filtering mode of the "*XenoComparator*" tab, you can obtain all the information of the peptide sequences from a given protein within the proteome of interest and the information of the occurrence of those peptide sequences within the host/background proteome.

Select the database for the proteome of interest and the database for the host/background proteome. Type the protein ID in the textbox and click on the corresponding "**Search**" button.

A window prompting you to choose the use or not of default filters should appear (Figure 39). Default filters are as follows: peptide size low limit of 5 amino acids, peptide size high limit of 22 amino acids, hit limit of 1 within the proteome of interest, hit limit of 0 within the host/background proteome, no miscleavage, no cysteine residue, no methionine residue, no tryptophan residue. Use default filters if you want to select unique peptide for targeted proteomics experiments with the existence of a host/background proteome. Those peptide sequences are unique within the proteome of interest and are not present in the host/background proteome. Click on "**Yes**" if you want to use the default filters (Figure 40). Click on "**No**" if you want the unfiltered list of peptide sequences related to the protein (Figure 41). The results are displayed in the data grid. The numbers of peptide sequences displayed (depending on the filters used) as well as the total number of peptide sequences for the given protein are indicated at the bottom of the window.

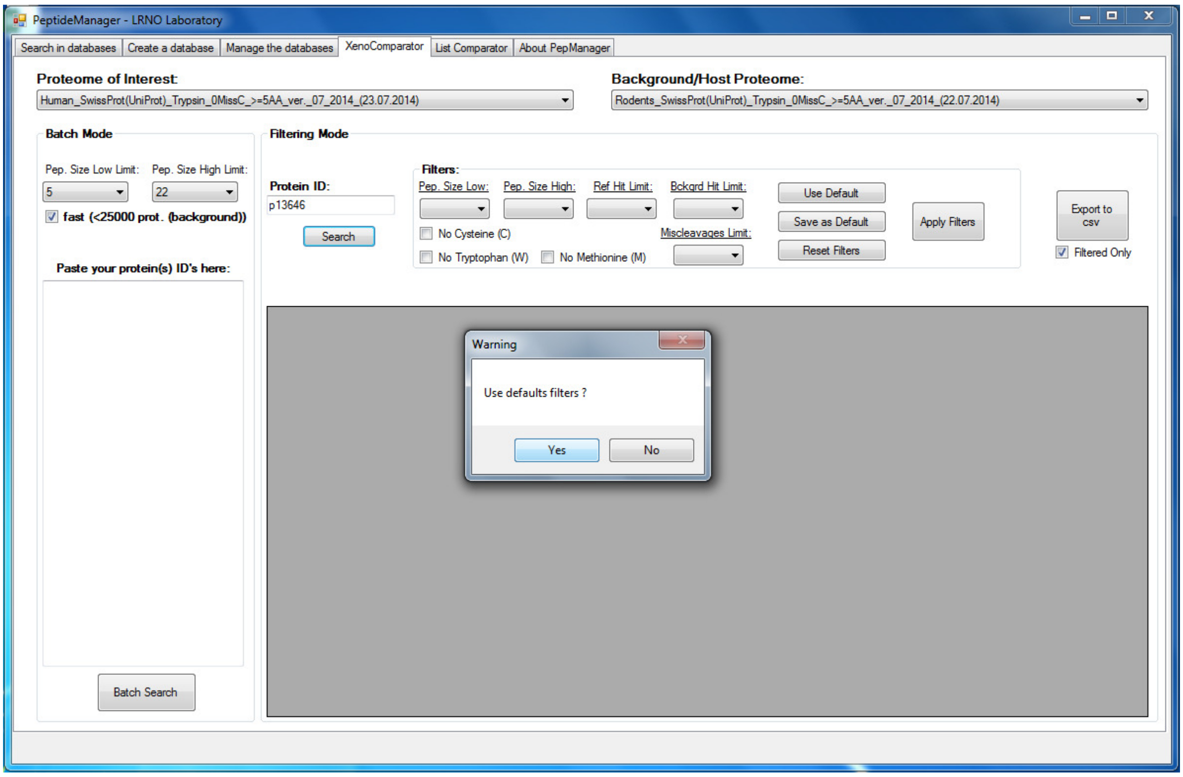

Figure 39.

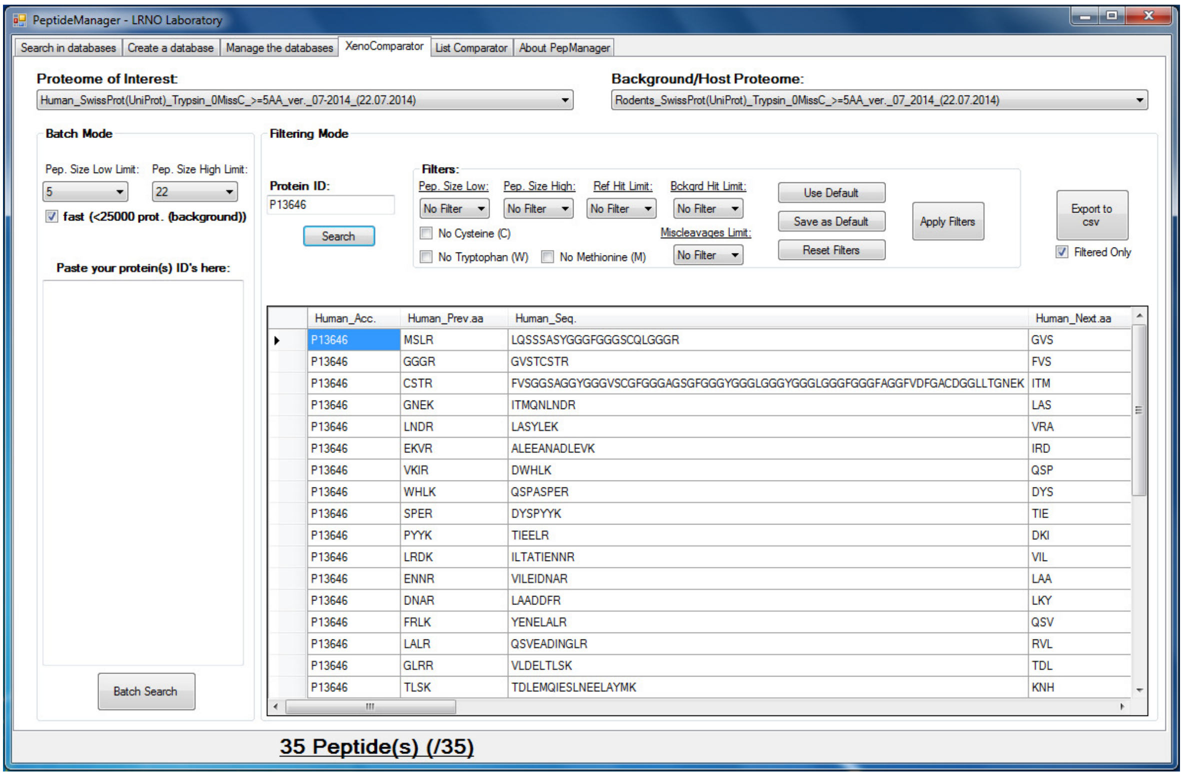

Figure 40.

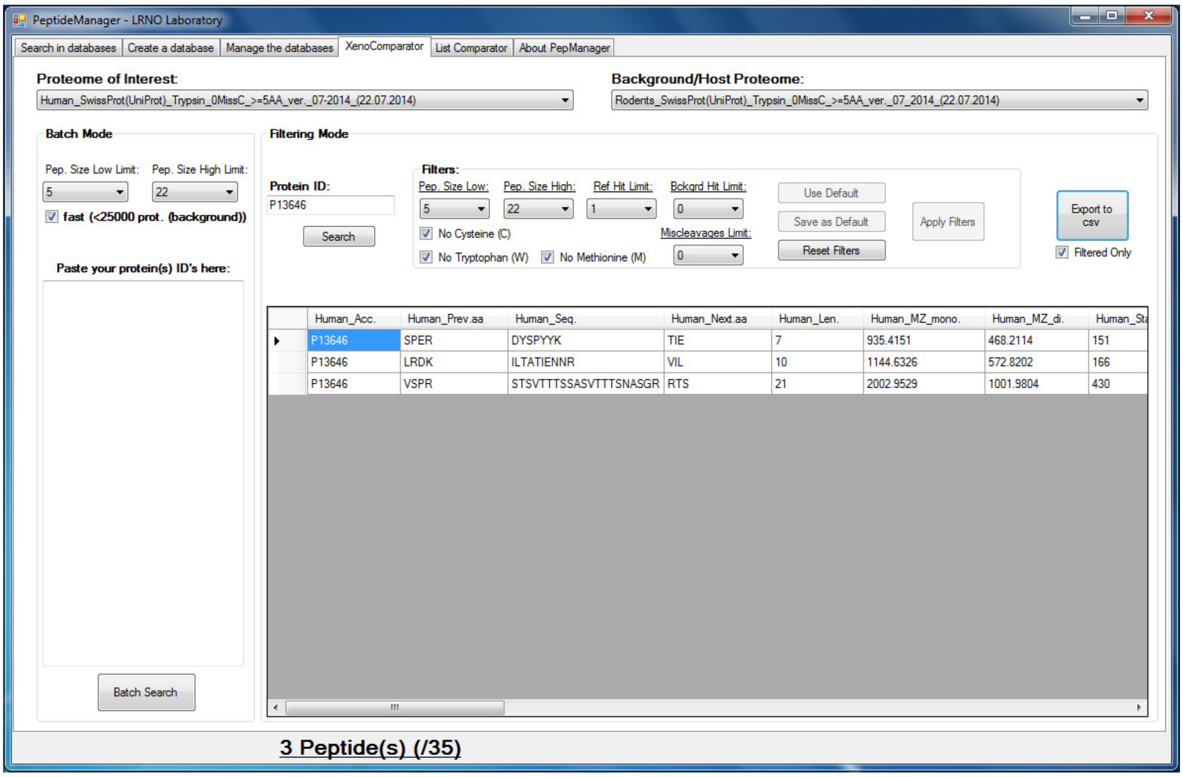

Figure 41.

You can modify any of the filters. Click on “**Apply Filters**” to apply any change.

You can modify the values of the default filters. Modify the filters as whished and click on “**Save as Default**”.

Click on “**Reset Filters**” to reset to zero the filters (not the default values). Click on “**Apply Filters**” to obtain the unfiltered list of peptide sequences related to the protein.

Click on “**Use Default**” to set the default values of the different filters. Click on “**Apply Filters**” to obtain the filtered list of peptide sequences related to the protein with the default filters.

You have the possibility to delete any row of the data grid. Just click on the left of the row (Figure 42) to highlight the row to delete and then press the delete touch of your keyboard (Figure 43).

To export the list of peptide sequences related to the given protein in CSV format, click on “**Export to CSV**” (Figure 44). If you want to export only the filtered results, let the checkbox “*Filtered only*” in the checked status (Figure 45). By unchecking this checkbox, you will export the unfiltered results; *i.e.*, all the peptide sequences related to the given protein (Figure 46).

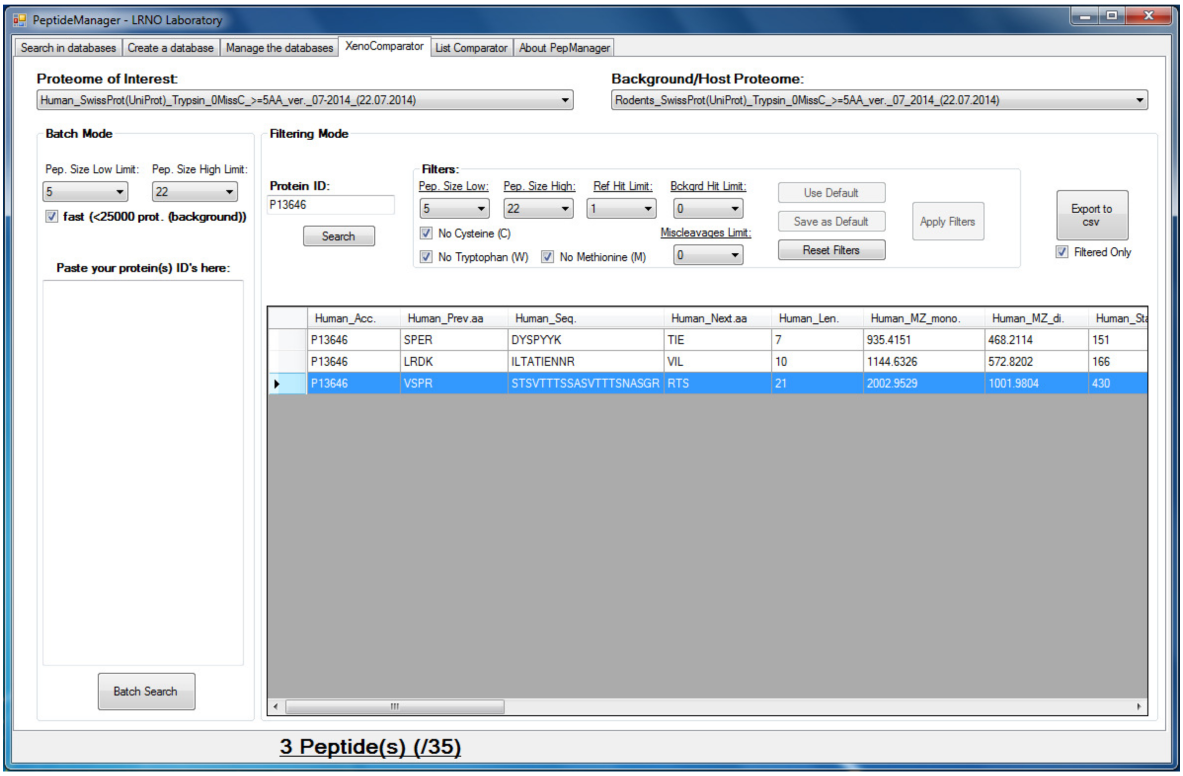

Figure 42.

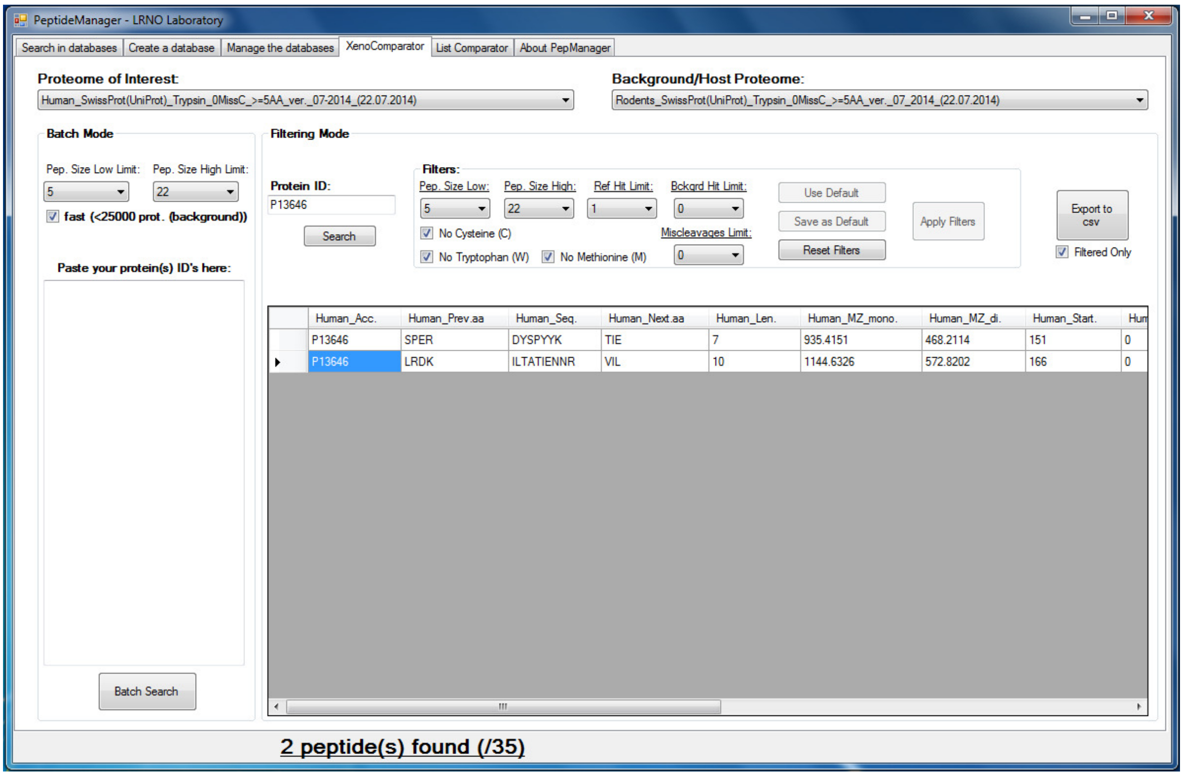

Figure 43.

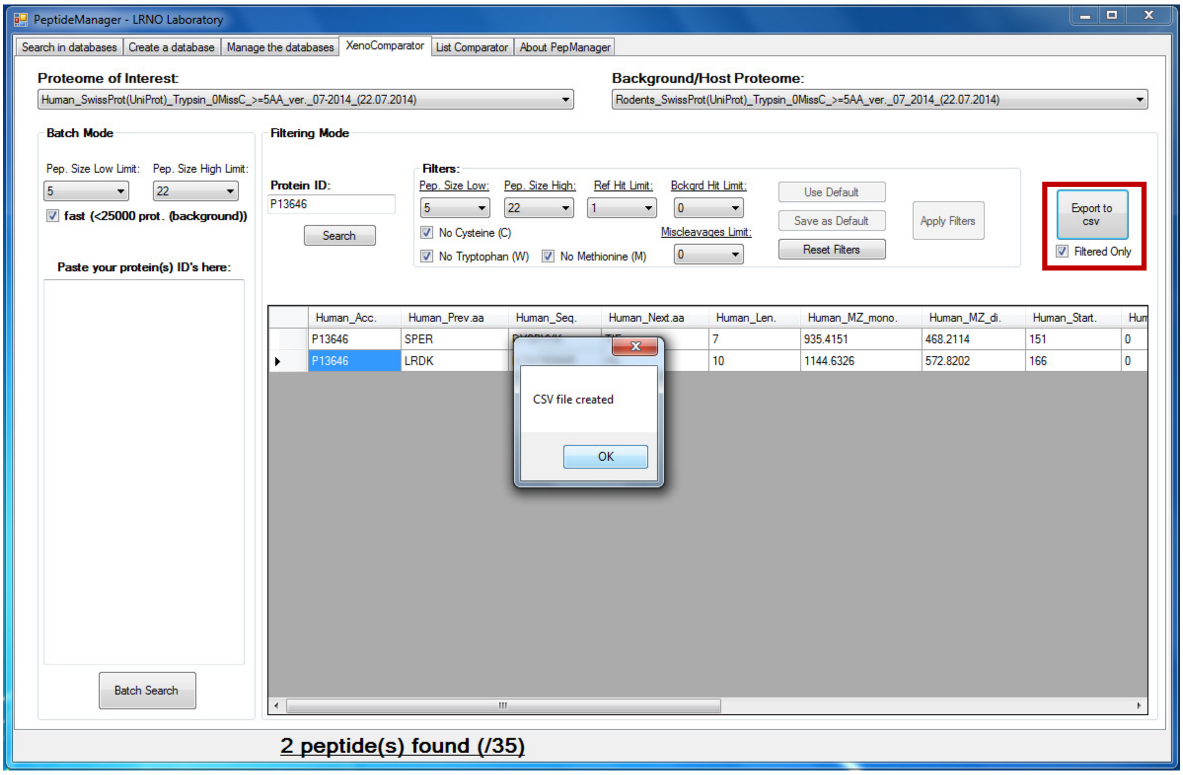

Figure 44.

Human_Acc.	Human_Pr	Human_Seq.	Human_Ne	Human_Len.	Human_MZ_mo	Human_MZ_d	Human_Sta	Human_Miscle	Human_Hit(s)	Human_Feat.	Rodents_Hit(s)	Rodents_Acc.	Pep_Len.	Pep_C	Pep_M	Pep_W
P13646	SPER	DYSPYYK	TIE	7	935.4151	468.2114	151	0	1		0		FALSE	FALSE	FALSE	FALSE
P13646	LRDK	ILTATIENNR	VIL	10	1144.6326	572.8202	166	0	1		0		FALSE	FALSE	FALSE	FALSE

P13646(Human)_vs_Rodents_FILTER

Figure 45.

Human_Acc.	Human_Pr	Human_Seq.	Human_Ne	Human_Len.	Human_MZ_mo	Human_MZ_d	Human_St	Human_Mis	Human_Hit(s)	Human_Feat	Rodents_Hit(s)	Rodents_Acc.	Pep_Len.	Pep_C	Pep_M	Pep_W
P13646	MSLR	LQSSSASYGGGFGGGS	GVS	23	2089.9361	1045.472	5	0	1		0		FALSE	TRUE	FALSE	FALSE
P13646	GGGR	GVSTCSTR	FVS	8	810.378	405.6929	28	0	1		0		FALSE	TRUE	FALSE	FALSE
P13646	CSTR	FVSGGSAGGYGGGVS	ITM	70	5937.5999	2969.3039	36	0	1	[VAR_SEQ] [6	0		FALSE	TRUE	FALSE	FALSE
P13646	GNEK	ITMQNLNDR	LAS	9	1104.5472	552.7775	106	0	2	[VARIANT] [1(	5	P08730;Q6IFV	FALSE	FALSE	TRUE	FALSE
P13646	LNDR	LASYLEK	VRA	7	823.4565	412.2322	115	0	8	[VARIANT] [1	4	Q6IFV4;P2503(	FALSE	FALSE	FALSE	FALSE
P13646	EKVR	ALEEANADLEVK	IRD	12	1301.6589	651.3334	124	0	4		1	Q6IFX2	FALSE	FALSE	FALSE	FALSE
P13646	VKIR	DWHLK	QSP	5	698.3626	349.6852	138	0	1		2	P08730;Q6IFV	FALSE	FALSE	FALSE	TRUE
P13646	WHLK	QSPASPER	DYS	8	871.4274	436.2176	143	0	1	[VARIANT] [1	1	P08730	FALSE	FALSE	FALSE	FALSE
P13646	SPER	DYSPYYK	TIE	7	935.4151	468.2114	151	0	1		0		FALSE	FALSE	FALSE	FALSE
P13646	PYYK	TIEELR	DKI	6	760.4205	380.7142	158	0	3		3	B1H234;P0873	FALSE	FALSE	FALSE	FALSE
P13646	LRDK	ILTATIENNR	VIL	10	1144.6326	572.8202	166	0	1		0		FALSE	FALSE	FALSE	FALSE
P13646	ENNR	VILEIDNAR	LAA	9	1042.5897	521.7988	176	0	2		1	Q6IFV3	FALSE	FALSE	FALSE	FALSE
P13646	DNAR	LAADDFR	LKY	7	807.4001	404.204	185	0	17	[VARIANT] [1	27		FALSE	FALSE	FALSE	FALSE
P13646	FRLK	YENELALR	QSV	8	1007.5162	504.262	194	0	3		4	Q64291;Q6IFW	FALSE	FALSE	FALSE	FALSE
P13646	LALR	QSVEADINGLR	RVL	11	1201.6177	601.3128	202	0	3		4	P02535;Q6IFW	FALSE	FALSE	FALSE	FALSE
P13646	GLRR	VLDELTLSK	TDL	9	1017.5832	509.2955	214	0	1		2	P02535;Q6IFW	FALSE	FALSE	FALSE	FALSE
P13646	TLSK	TDLEMQIESLNEELAYN	KNH	18	2157.0095	1079.0087	223	0	2		0		FALSE	FALSE	TRUE	FALSE
P13646	YMKK	NHEEEMK	EFS	7	916.3835	458.6956	242	0	6		8	Q6IFW6;P0873	FALSE	FALSE	TRUE	FALSE
P13646	EEMK	EFSNQVVGQVNVEMD	VLA	24	2619.2725	1310.1401	249	0	1		1	P08730	FALSE	FALSE	TRUE	FALSE
P13646	DLTR	VLAEMR	EQY	6	718.3922	359.7	273	0	2		2	P08730;Q6IFV	FALSE	FALSE	TRUE	FALSE
P13646	AEMR	EQYEAMAER	NRR	9	1126.4839	563.7459	279	0	1		0		FALSE	FALSE	TRUE	FALSE
P13646	RNRR	DAEEWFHTK	SAE	9	1162.5169	581.7624	291	0	1	[VARIANT] [2	0		FALSE	FALSE	FALSE	TRUE
P13646	FHTK	SAELNK	EVS	6	661.3521	331.18	300	0	1		2	P08730;Q6IFV	FALSE	FALSE	FALSE	FALSE
P13646	ELNK	EVSTNTAMIQTSK	TEI	13	1409.6946	705.3512	306	0	1		0		FALSE	FALSE	TRUE	FALSE
P13646	QTSK	TEITELR	RTL	7	861.4682	431.238	319	0	1		4	P08730;Q6IFV	FALSE	FALSE	FALSE	FALSE
P13646	ELRR	TLQGLEIELQSQLSMK	AGL	16	1817.9683	909.4881	327	0	2		5	P08730;Q6IFV	FALSE	FALSE	TRUE	FALSE
P13646	LSMK	AGLENTVAETECR	YAL	13	1392.6429	696.8254	343	0	1	[VAR_SEQ] [3	0		FALSE	TRUE	FALSE	FALSE
P13646	TECR	YALQLQQIQGLISSIEA(	SEM	23	2601.4252	1301.2165	356	0	1	[VAR_SEQ] [3	1	P08730	FALSE	FALSE	FALSE	FALSE
P13646	SELR	SEMECQNQEYK	MLL	11	1388.5462	694.777	379	0	1	[VAR_SEQ] [3	2	P08730;Q6IFV	FALSE	TRUE	TRUE	FALSE
P13646	QEYK	MLLDIK	TRL	6	732.433	366.7204	390	0	2	[VAR_SEQ] [3	2	P08730;Q6IFV	FALSE	FALSE	TRUE	FALSE
P13646	IKTR	LEQEIATYR	SLL	9	1122.5795	561.7937	398	0	8	[VAR_SEQ] [3	16		FALSE	FALSE	FALSE	FALSE

P13646(Human)_vs_Rodents

Figure 46.

It is worth noting that the presence of a host/background proteome will decrease the number of unique peptide sequences and, in the worst cases, there will be no possible unique peptide sequence. Without a host/background proteome, 5 unique peptides are found for the P13646 protein (Figures 47 and 48) whereas only 2 unique peptides are found with the presence of the rodents host/background proteome.

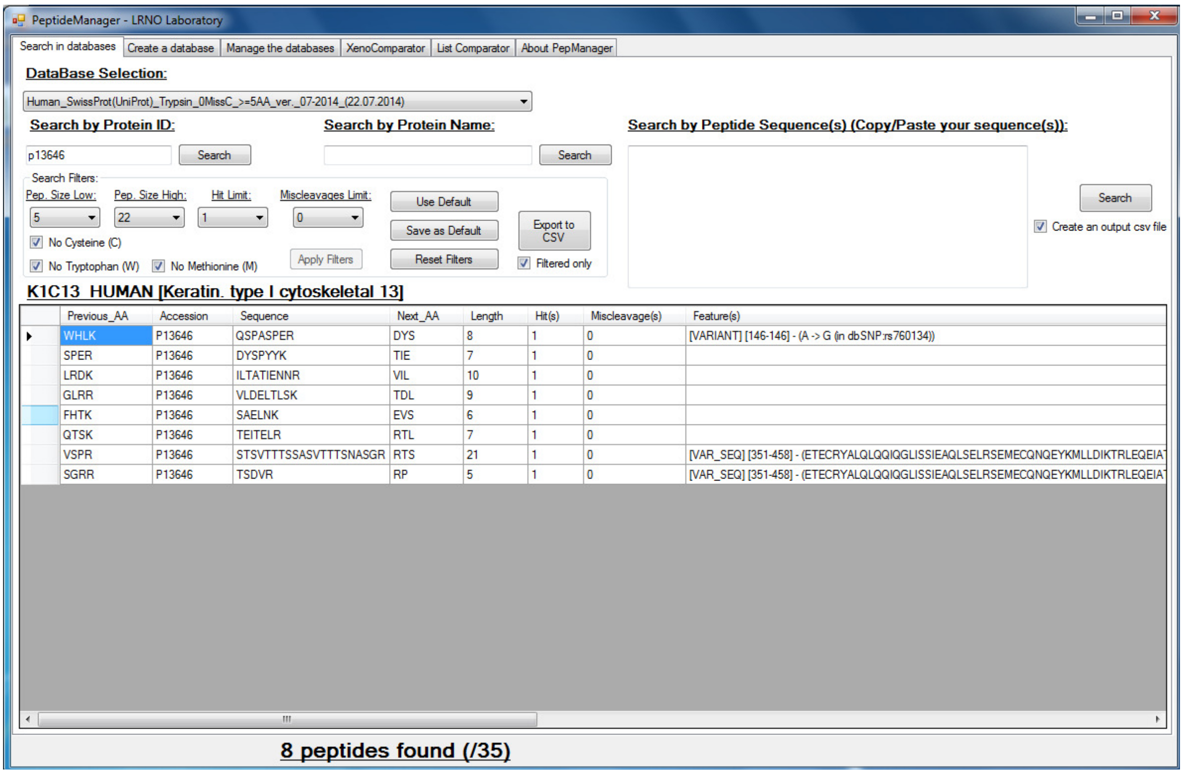

Figure 47.

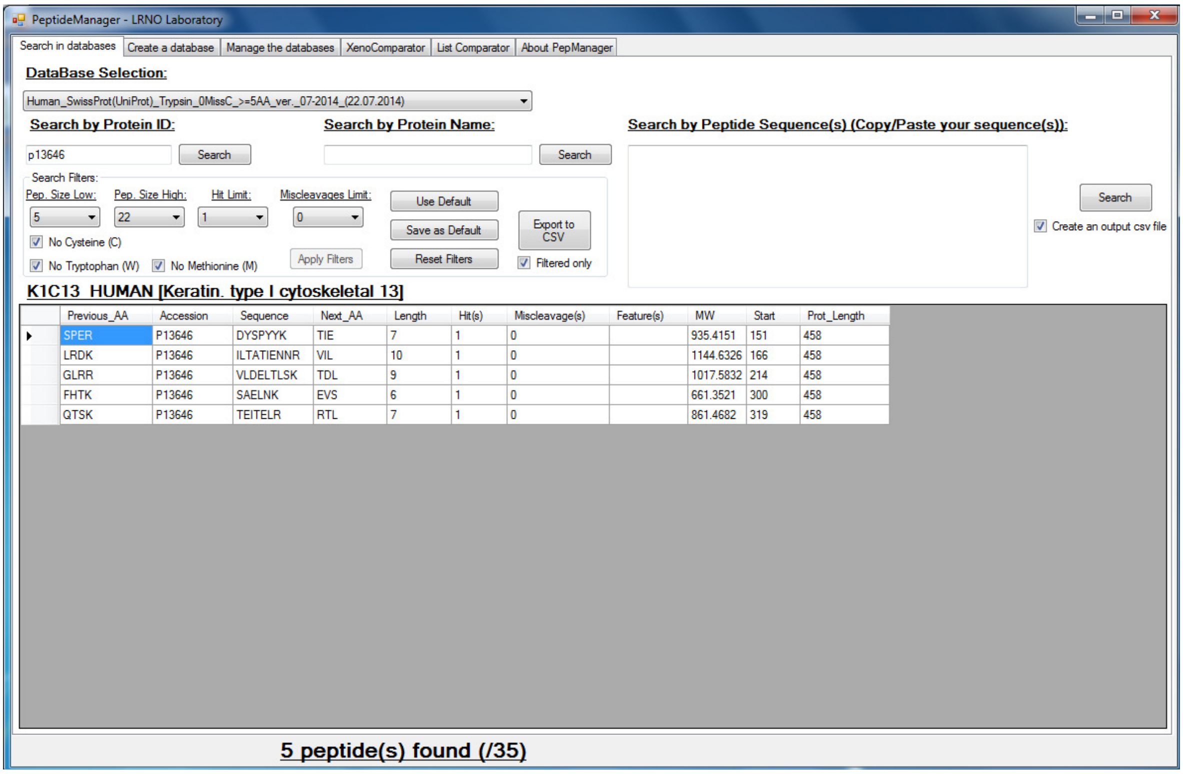

Figure 48

Batch Mode

If you have to select unique peptide sequences for a large number of proteins, you can obtain the csv files of the peptide sequences for all the proteins in one step thanks to the "*batch mode*" of the "*XenoComparator*" tab of PeptideManager (Figure 49). To allow you to modify *a posteriori* the filters, the obtained csv files contain all the peptide sequences. However, as shown on Figure 50, an additional column (the first one) indicates for each peptide sequences whether it would have been filtered out or not by the default filters.

To use the batch mode, select the database for the proteome of interest and the database for the host/background proteome. Type the protein IDs or copy/paste them in the textbox and click on the "**Batch Search**" button (Figure 49). You will be asked to choose the output folder where the csv files will be created (Figure 51).

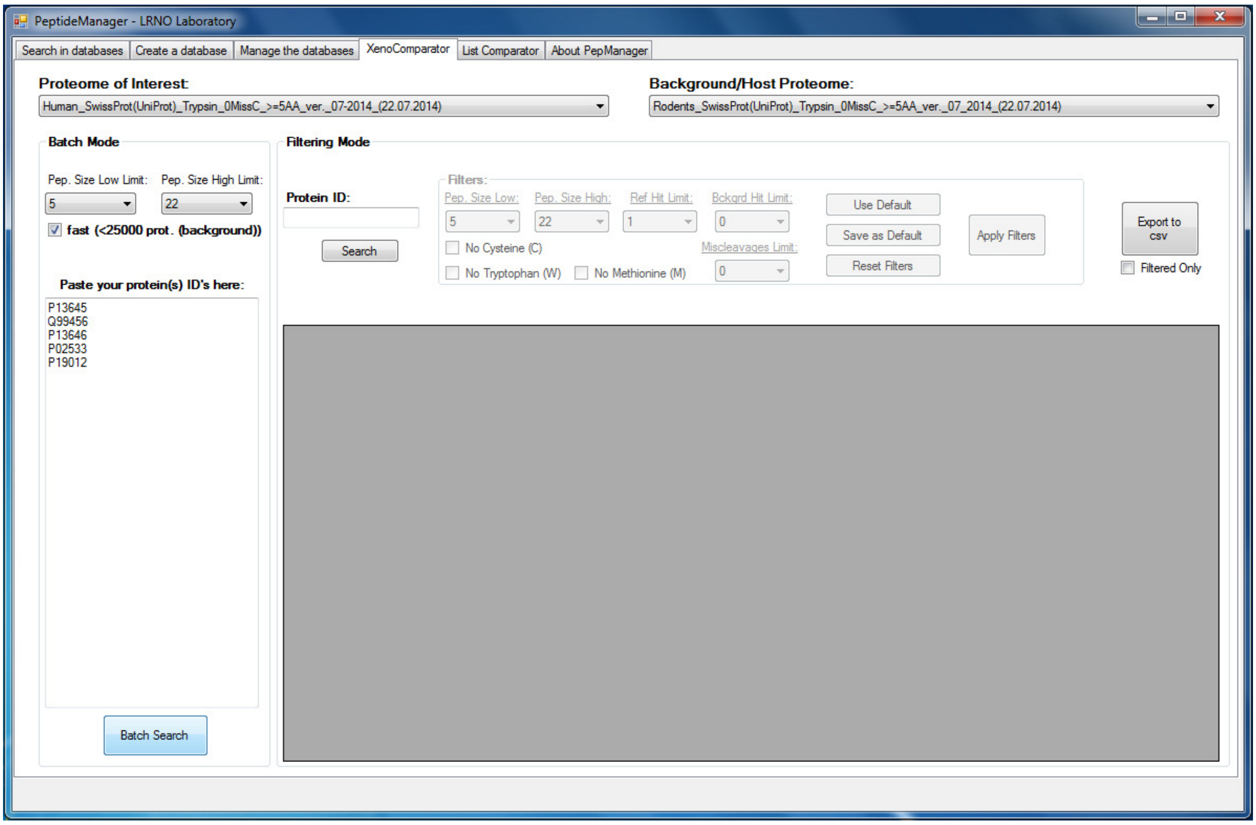

Figure 49.

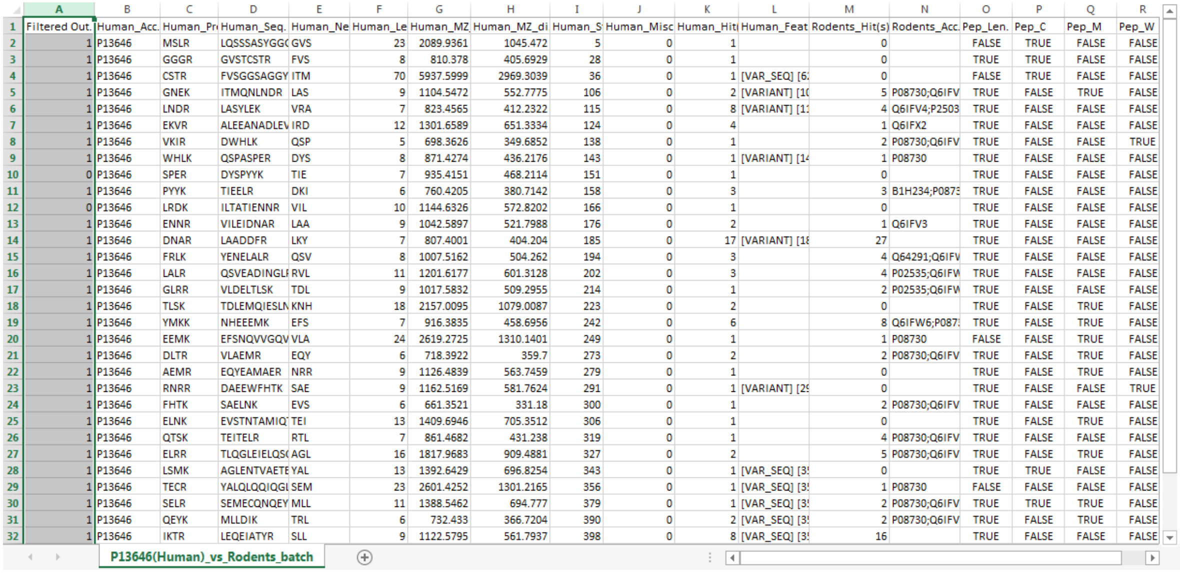

	A	B	C	D	E	F	G	H	I	J	K	L	M	N	O	P	Q	R
1	Filtered Out.	Human_Acc.	Human_Pr	Human_Seq.	Human_Ne	Human_Le	Human_MZ	Human_MZ_di	Human_S	Human_Misc	Human_Hit	Human_Feat	Rodents_Hit(s)	Rodents_Acc.	Pep_Len.	Pep_C	Pep_M	Pep_W
2	1	P13646	MSLR	LQSSSASYGG	GVS	23	2089.9361	1045.472	5	0	1		0		FALSE	TRUE	FALSE	FALSE
3	1	P13646	GGGR	GVSTCSTR	FVS	8	810.378	405.6929	28	0	1		0		TRUE	TRUE	FALSE	FALSE
4	1	P13646	CSTR	FVSGGSAGGY	ITM	70	5937.5999	2969.3039	36	0	1	[VAR_SEQ] [6	0		FALSE	TRUE	FALSE	FALSE
5	1	P13646	GNEK	ITMQNLNDR	LAS	9	1104.5472	552.7775	106	0	2	[VARIANT] [10	5	P08730;Q6IFV	TRUE	FALSE	TRUE	FALSE
6	1	P13646	LNDR	LASYLEK	VRA	7	823.4565	412.2322	115	0	8	[VARIANT] [1	4	Q6IFV4;P2503	TRUE	FALSE	FALSE	FALSE
7	1	P13646	EKVR	ALEEANADLEV	IRD	12	1301.6589	651.3334	124	0	4		1	Q6IFX2	TRUE	FALSE	FALSE	FALSE
8	1	P13646	VKIR	DWHLK	QSP	5	698.3626	349.6852	138	0	1		2	P08730;Q6IFV	TRUE	FALSE	FALSE	TRUE
9	1	P13646	WHLK	QSPASPER	DYS	8	871.4274	436.2176	143	0	1	[VARIANT] [14	1	P08730	TRUE	FALSE	FALSE	FALSE
10	0	P13646	SPER	DYSPYYK	TIE	7	935.4151	468.2114	151	0	1		0		TRUE	FALSE	FALSE	FALSE
11	1	P13646	PYYK	TIEELR	DKI	6	760.4205	380.7142	158	0	3		3	B1H234;P087	TRUE	FALSE	FALSE	FALSE
12	0	P13646	LRDK	ILTATIENNR	VIL	10	1144.6326	572.8202	166	0	1		0		TRUE	FALSE	FALSE	FALSE
13	1	P13646	ENNR	VILEIDNAR	LAA	9	1042.5897	521.7988	176	0	2		1	Q6IFV3	TRUE	FALSE	FALSE	FALSE
14	1	P13646	DNAR	LAADDFR	LKY	7	807.4001	404.204	185	0	17	[VARIANT] [18	27		TRUE	FALSE	FALSE	FALSE
15	1	P13646	FRLK	YENELALR	QSV	8	1007.5162	504.262	194	0	3		4	Q64291;Q6IFV	TRUE	FALSE	FALSE	FALSE
16	1	P13646	LALR	QSVEADINGL	RVL	11	1201.6177	601.3128	202	0	3		4	P02535;Q6IFW	TRUE	FALSE	FALSE	FALSE
17	1	P13646	GLRR	VLDELTLSK	TDL	9	1017.5832	509.2955	214	0	1		2	P02535;Q6IFW	TRUE	FALSE	FALSE	FALSE
18	1	P13646	TLSK	TDLEMQIESLN	KNH	18	2157.0095	1079.0087	223	0	2		0		TRUE	FALSE	TRUE	FALSE
19	1	P13646	YMKK	NHEEEMK	EFS	7	916.3835	458.6956	242	0	6		8	Q6IFW6;P087	TRUE	FALSE	TRUE	FALSE
20	1	P13646	EEMK	EFSNQVVGQV	VLA	24	2619.2725	1310.1401	249	0	1		1	P08730	FALSE	FALSE	TRUE	FALSE
21	1	P13646	DLTR	VLAEMR	EQY	6	718.3922	359.7	273	0	2		2	P08730;Q6IFV	TRUE	FALSE	TRUE	FALSE
22	1	P13646	AEMR	EQYEAMAER	NRR	9	1126.4839	563.7459	279	0	1		0		TRUE	FALSE	TRUE	FALSE
23	1	P13646	RNRR	DAEEWFHTK	SAE	9	1162.5169	581.7624	291	0	1	[VARIANT] [2	0		TRUE	FALSE	FALSE	TRUE
24	1	P13646	FHTK	SAELNK	EVS	6	661.3521	331.18	300	0	1		2	P08730;Q6IFV	TRUE	FALSE	FALSE	FALSE
25	1	P13646	ELNK	EVSTNTAMIQ	TEI	13	1409.6946	705.3512	306	0	1		0		TRUE	FALSE	TRUE	FALSE
26	1	P13646	QTSK	TEITELR	RTL	7	861.4682	431.238	319	0	1		4	P08730;Q6IFV	TRUE	FALSE	FALSE	FALSE
27	1	P13646	ELRR	TLQGLEIELQS	AGL	16	1817.9683	909.4881	327	0	2		5	P08730;Q6IFV	TRUE	FALSE	TRUE	FALSE
28	1	P13646	LSMK	AGLENTVAETE	YAL	13	1392.6429	696.8254	343	0	1	[VAR_SEQ] [3	0		TRUE	TRUE	FALSE	FALSE
29	1	P13646	TECR	YALQLQQIQGL	SEM	23	2601.4252	1301.2165	356	0	1	[VAR_SEQ] [3	1	P08730	FALSE	FALSE	FALSE	FALSE
30	1	P13646	SELR	SEMECQNQEY	MLL	11	1388.5462	694.777	379	0	1	[VAR_SEQ] [3	2	P08730;Q6IFV	TRUE	TRUE	TRUE	FALSE
31	1	P13646	QEYK	MLLDIK	TRL	6	732.433	366.7204	390	0	2	[VAR_SEQ] [3	2	P08730;Q6IFV	TRUE	FALSE	TRUE	FALSE
32	1	P13646	IKTR	LEQEIATYR	SLL	9	1122.5795	561.7937	398	0	8	[VAR_SEQ] [3	16		TRUE	FALSE	FALSE	FALSE

Figure 50.

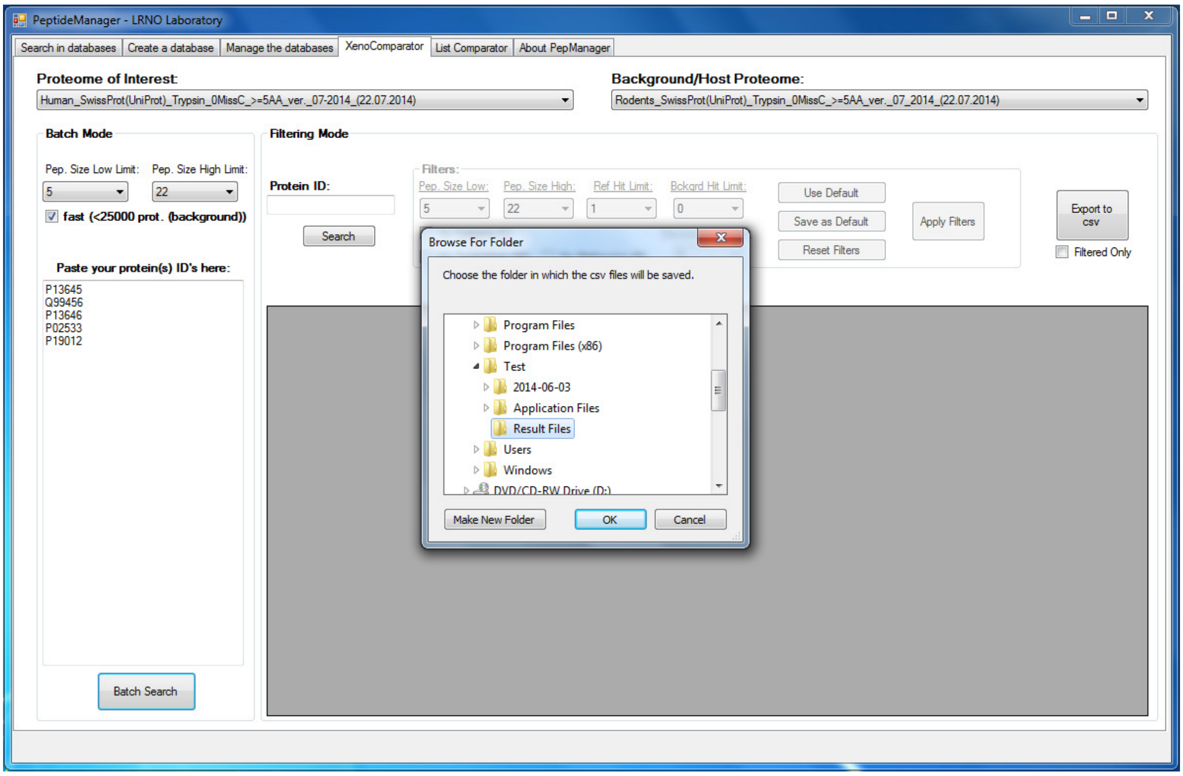

Figure 51.

As shown on the Figure 52, a progress bar indicates you the progress of the process. Once the process is finished (Figure 53), you can access the csv files whose filenames contain the protein ID, the taxonomy of the proteome of interest and the taxonomy of the host/background proteome (Figure 54).

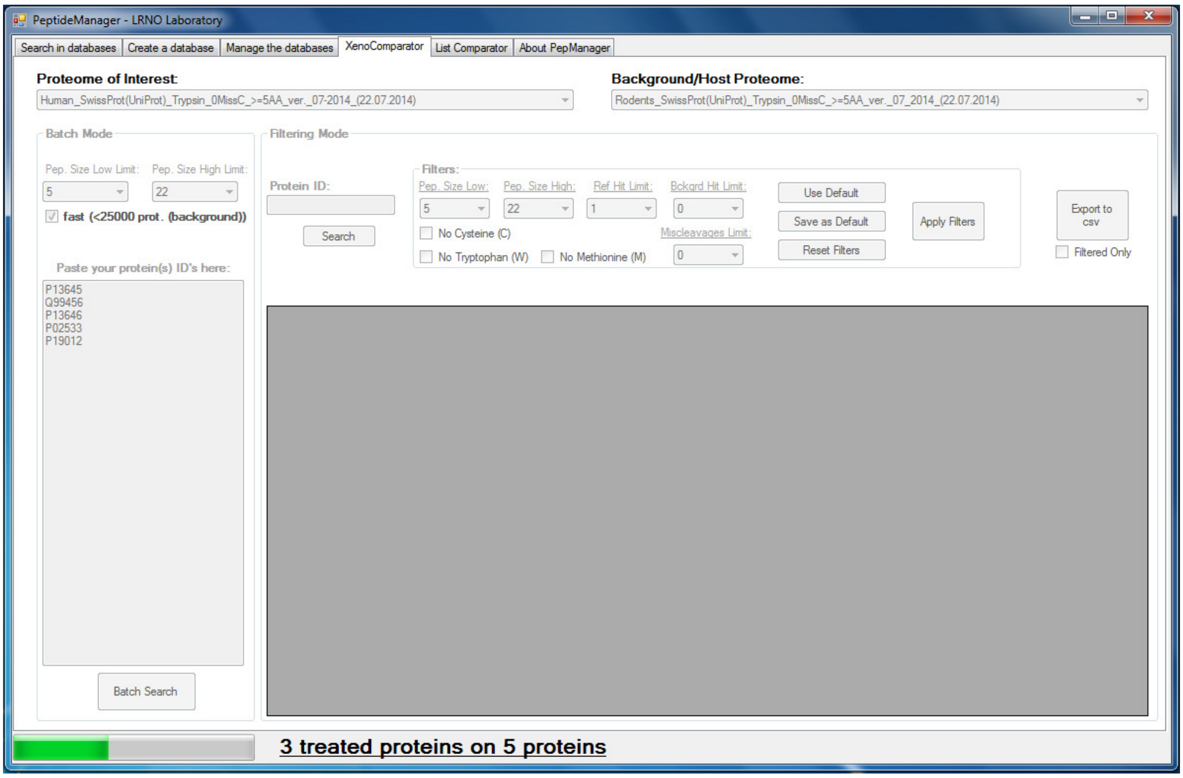

Figure 52.

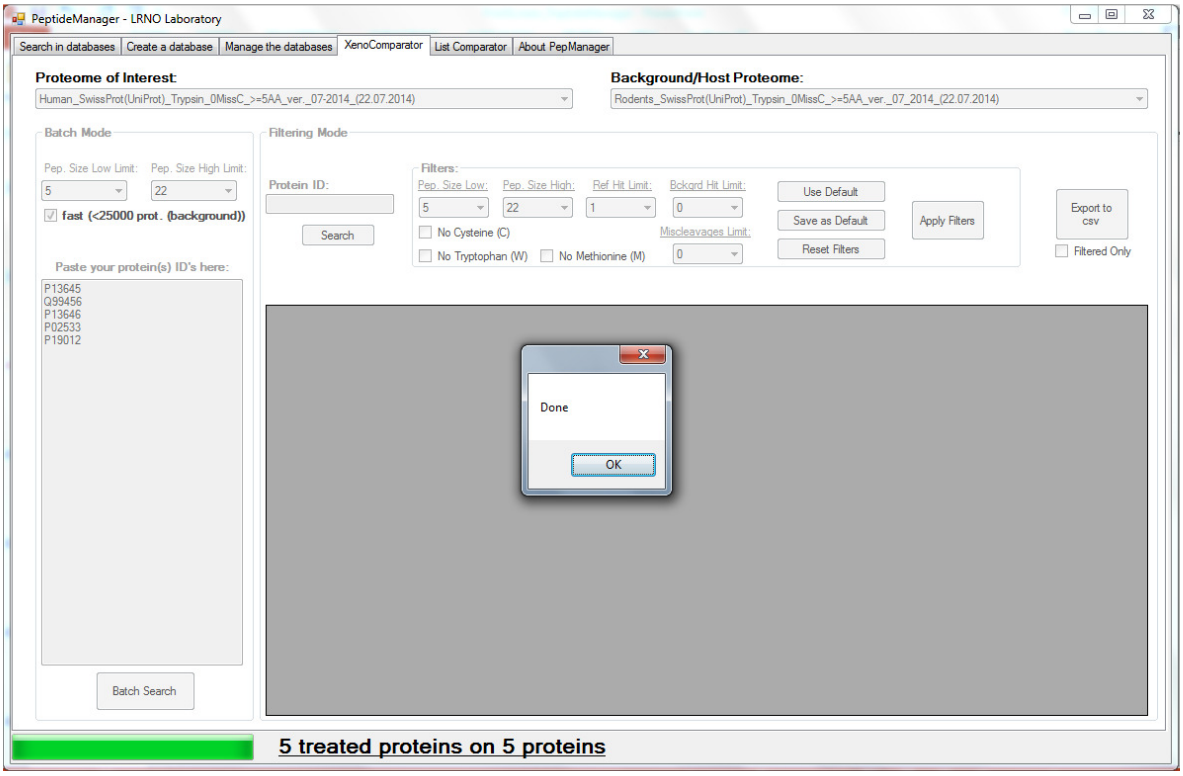

Figure 53.

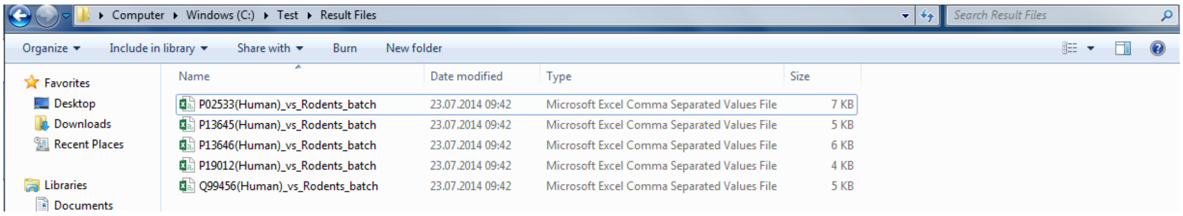

Figure 54.

Case study: Selection of unique peptide sequences for a targeted proteomics experiment with the presence of a background/host proteome

In order to compare with the case study in the absence of host/background proteome, this case study will focus to target the P12532 protein (human) with the presence of the rodents proteome as background. After having selected both databases, typed "P12532" in the "Protein ID" textbox of the "*Filtering Mode*" part of the "*XenoComparator*" tab of PeptideManager and clicked on the corresponding "**Search**" button, you should obtain the results shown on Figure 55.

The first peptide sequence in the list of results can be discarded because it is a part of the "Transit peptide" part of the protein and transit peptides are generally cleaved from the mature protein. In the end, only two peptide sequences are obtained (Figure 56) in presence of the host/background proteome whereas 7 unique peptides were found without the presence of a host/background proteome (Figure 30 on Page 22).

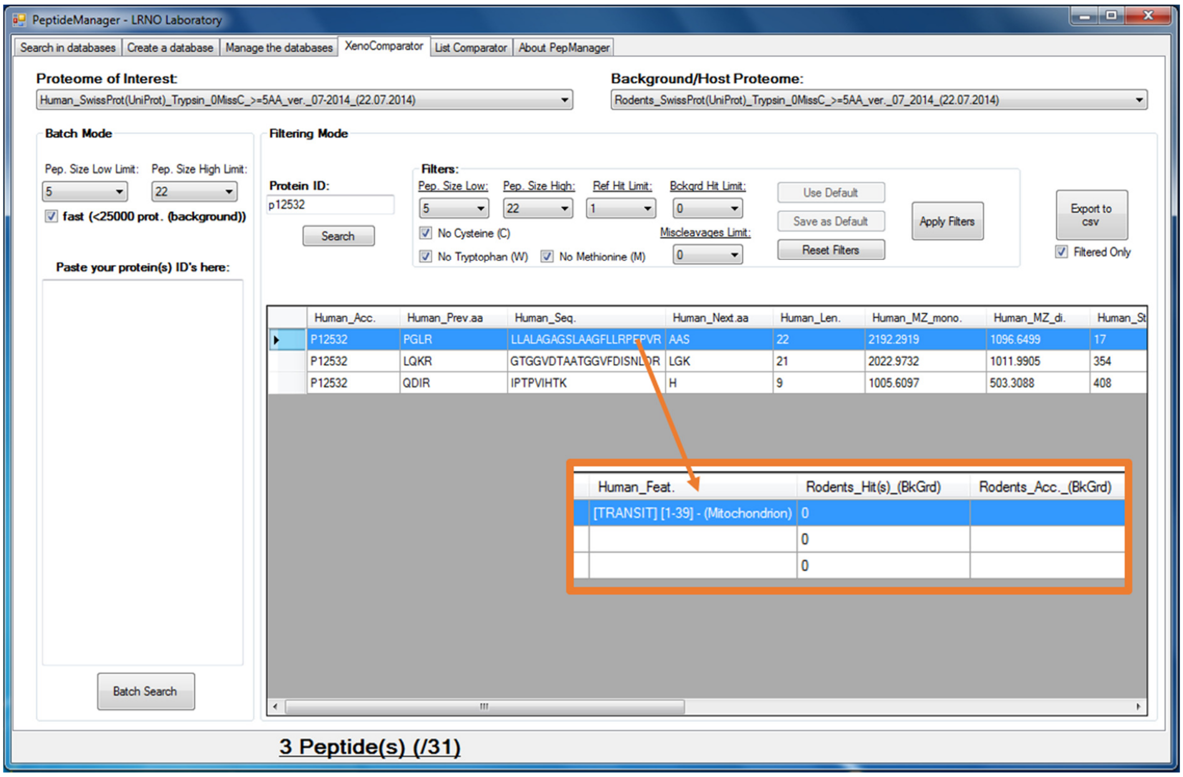

Figure 55.

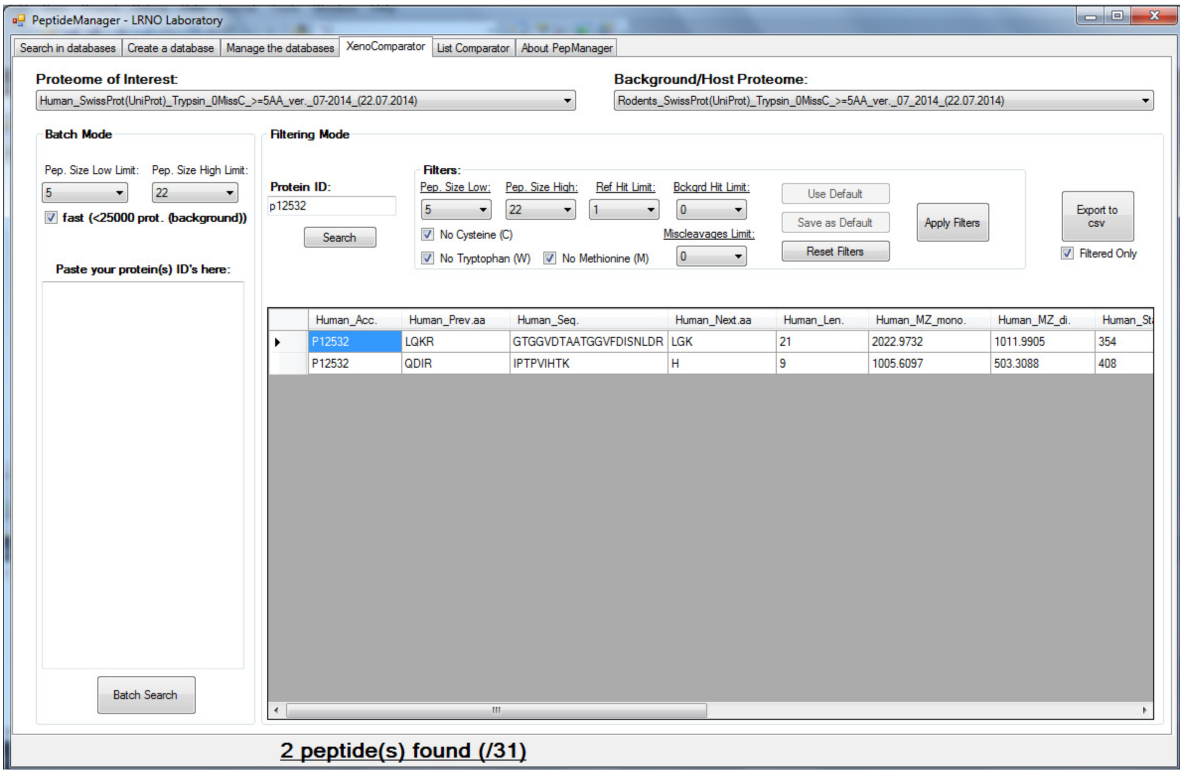
PeptideManager - LRNO Laboratory
Search in databases
Create a database
Manage the databases
XenoComparator
List Comparator
About PepManager
Proteome of Interest:
Human_SwissProt(UniProt)_Trypsin_0MissC_>=5AA_ver._07-2014_(22.07.2014)
Background/Host Proteome:
Rodents_SwissProt(UniProt)_Trypsin_0MissC_>=5AA_ver._07_2014_(22.07.2014)
Batch Mode
Pep. Size Low Limit:
Pep. Size High Limit:
fast (<25000 prot. (background))
Paste your protein(s) ID's here:
Batch Search
Filtering Mode
Protein ID:
p12532
Search
Filters:
Pep. Size Low:
Pep. Size High:
Ref Hit Limit:
Bckgrd Hit Limit:
Miscleavages Limit:
No Cysteine (C)
No Tryptophan (W)
No Methionine (M)
Use Default
Save as Default
Reset Filters
Apply Filters
Export to csv
Filtered Only
Human_Acc. | Human_Prev.aa | Human_Seq. | Human_Next.aa | Human_Len. | Human_MZ_mono. | Human_MZ_di.
P12532 | LQKR | GTGGVDTAATGGVFDISNLDR | LGK | 21 | 2022.9732 | 1011.9905 | 354
P12532 | QDIR | IPTPVIHTK | H | 9 | 1005.6097 | 503.3088 | 408
2 peptide(s) found (/31)

Figure 56.

Download the public protein databases

SwissProt/TrEMBL/UniProt

SwissProt/TrEMBL/UniProt public protein data repository can be accessed on the following website (Figure 57): http://www.uniprot.org/.

Predefined taxonomic divisions protein databases

To access the databases, click on the Download link (Figure 57). If it fits your needs, protein databases for some taxonomic divisions are available. To access those databases, click on the text link of the taxonomic divisions (Figure 58). The compressed database files (in the .dat format) for the different taxonomic divisions can be downloaded from the public ftp server of UniProt (Figure 59).

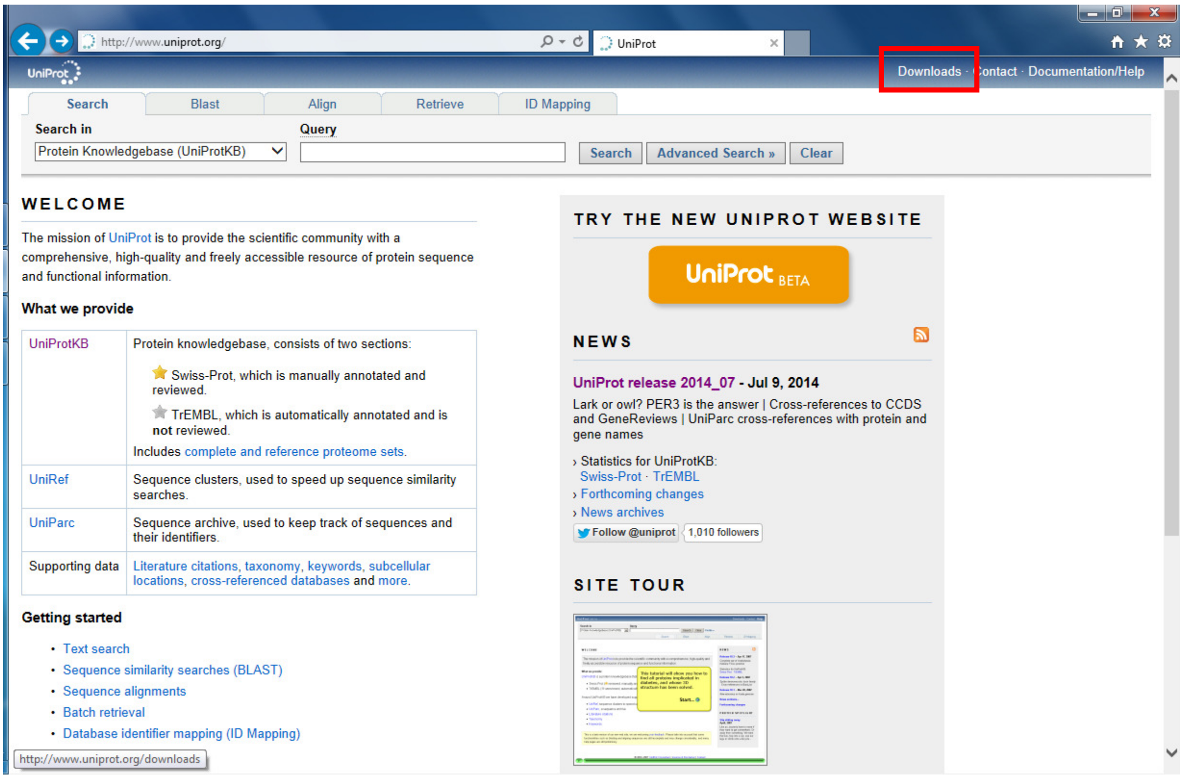

Figure 57.

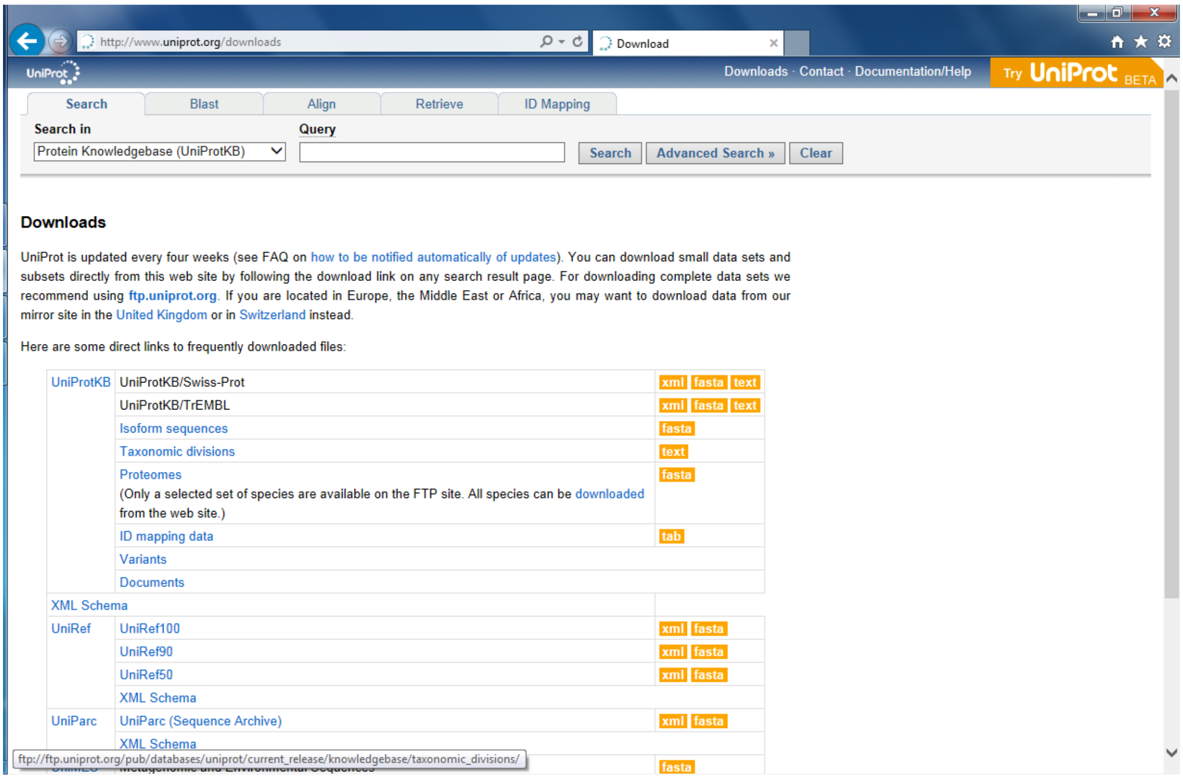

Figure 58.

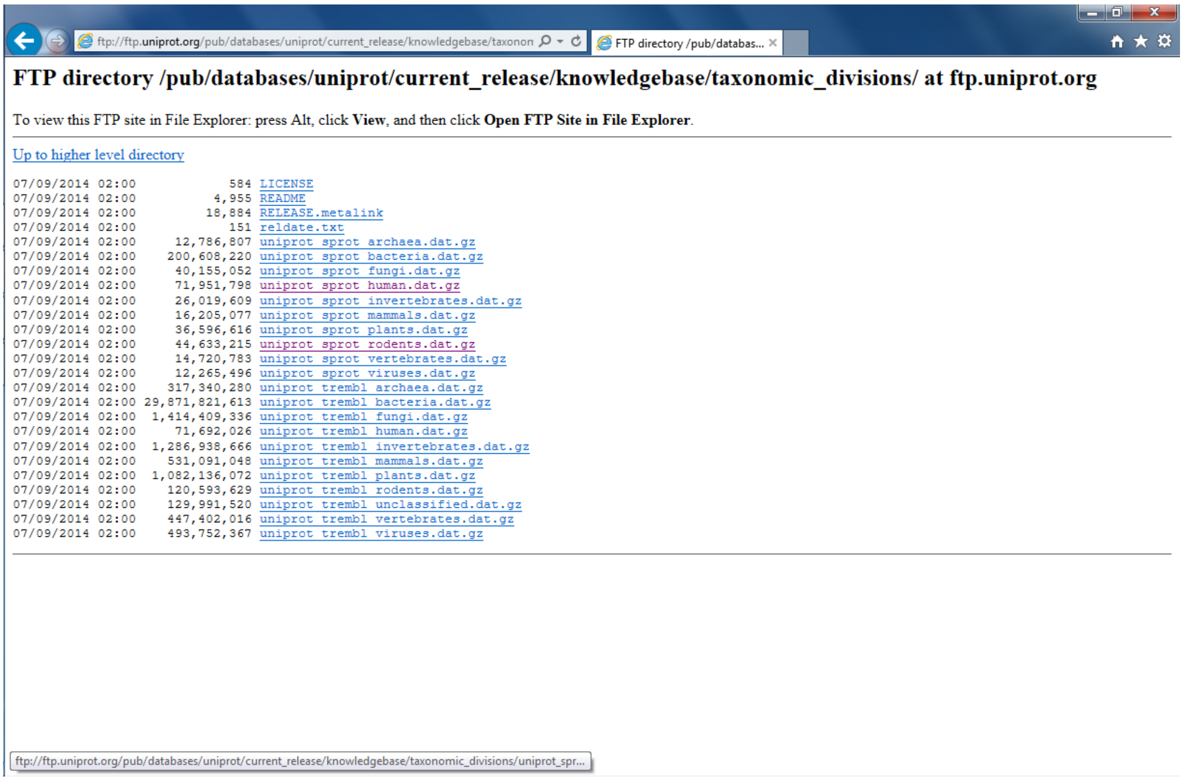

Figure 59.

Other taxonomic divisions

If your proteome of interest is not present in the standard taxonomic divisions, you can customized your protein selection in order to fit your needs.

From the "*Search*" tab of the main page of the UniProt website, you can access a given taxonomy by selecting "*Taxonomy*" in the "*Search In*" combo box (Figure 60) and typing the taxonomy number of interest (see http://www.ncbi.nlm.nih.gov/Taxonomy/Browser/wwwtax.cgi). You can reach higher and lower taxonomy levels by using the "Taxonomy Navigation" part (Figure 60). When you have selected you taxonomy of interest, click on the "**UniProtKB**" link. You can choose to select only (Figure 61) the reviewed proteins (SwissProt), the unreviewed proteins (TrEMBL) or both (UniProt). Once you have selected the set of proteins, click on the "**Download**" link (Figure 61) and click on the "**Download**" link of the "Flat Text" format (Figure 62) in order to download the database in the TXT format compatible with PeptideManager.

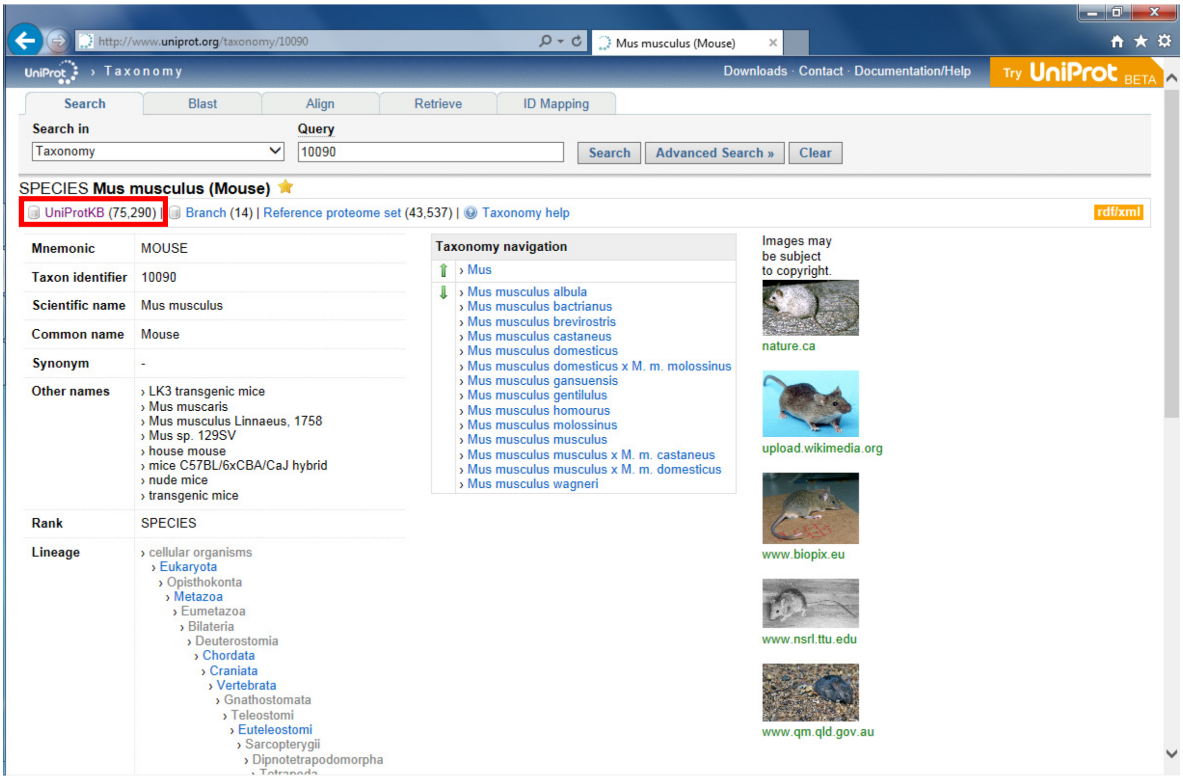

Figure 60.

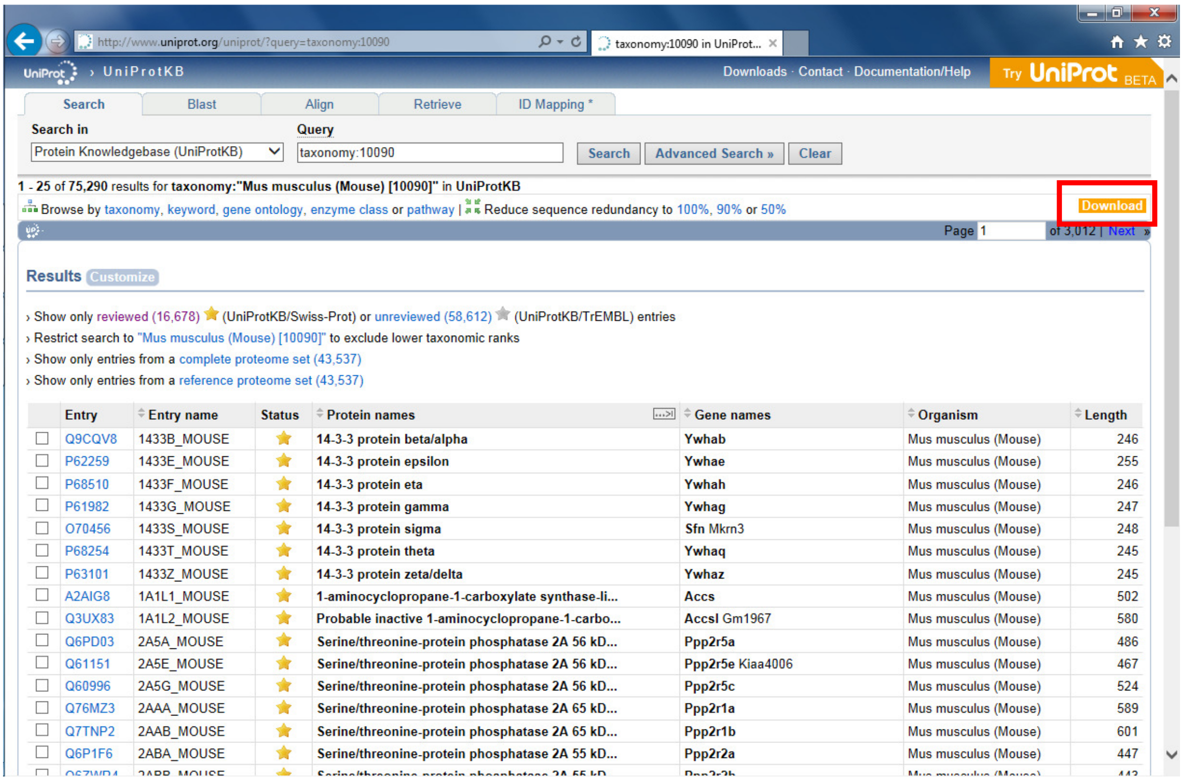

Figure 61.

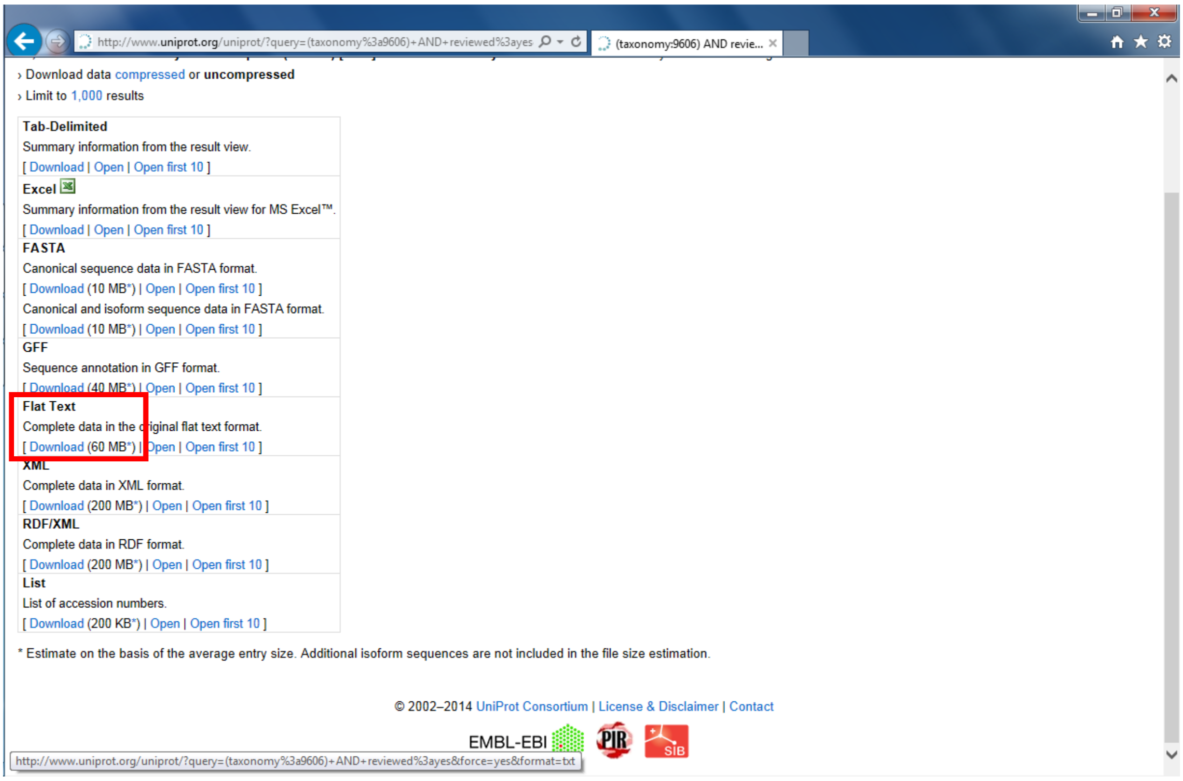

Figure 62.

RefSeq

The public protein databases from RefSeq can be accessed via the following website: http://www.ncbi.nlm.nih.gov/refseq/ (Figure 63). Click on "**FTP**" in the "*RefSeq Access*" section to reach the ftp server of RefSeq (Figure 64).

Select the directory of your taxonomy of interest (Figure 65) and then select the "*mRNA Prot*" folder. Two type of database files are available (Figure 66): the faa format which is a fasta-like format and contains less information (no protein features) and the gpff format which contains all the information available. Both formats are compatible with PeptideManager.

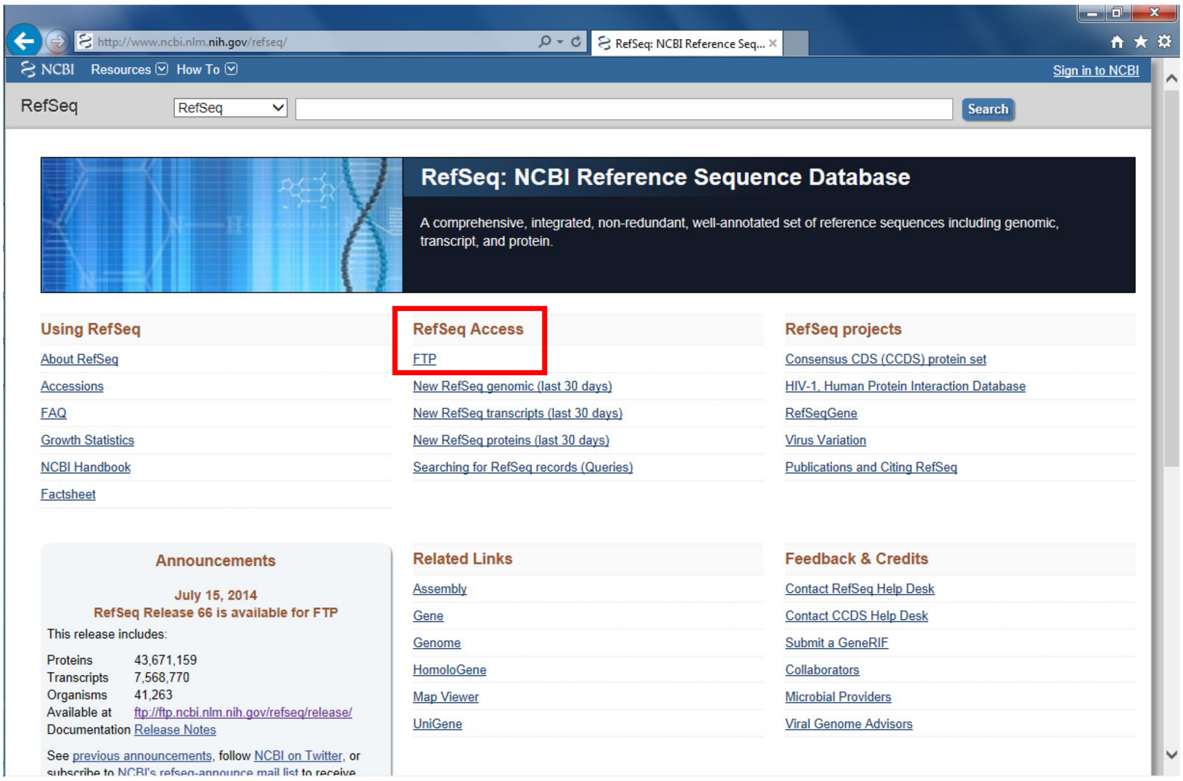

Figure 63.

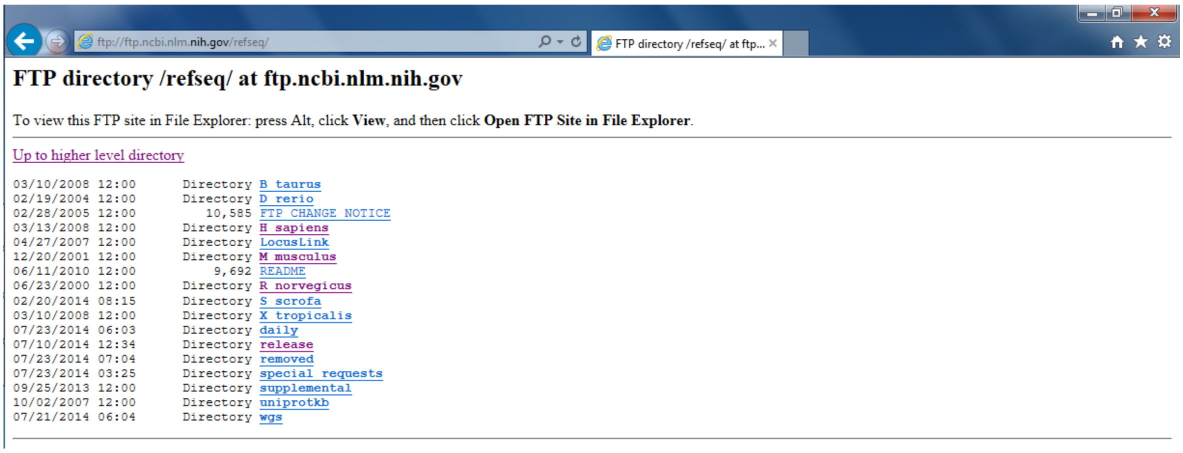
ftp://ftp.ncbi.nlm.nih.gov/refseq/

FTP directory /refseq/ at ftp.ncbi.nlm.nih.gov

To view this FTP site in File Explorer: press Alt, click **View**, and then click **Open FTP Site in File Explorer**.

Up to higher level directory

```
03/10/2008 12:00      Directory B taurus
02/19/2004 12:00      Directory D rerio
02/28/2005 12:00         10,585 FTP CHANGE NOTICE
03/13/2008 12:00      Directory H sapiens
04/27/2007 12:00      Directory LocusLink
12/20/2001 12:00      Directory M musculus
06/11/2010 12:00          9,692 README
06/23/2000 12:00      Directory R norvegicus
02/20/2014 08:15      Directory S scrofa
03/10/2008 12:00      Directory X tropicalis
07/23/2014 06:03      Directory daily
07/10/2014 12:34      Directory release
07/23/2014 07:04      Directory removed
07/23/2014 03:25      Directory special requests
09/25/2013 12:00      Directory supplemental
10/02/2007 12:00      Directory uniprotkb
07/21/2014 06:04      Directory wgs
```

Figure 64.

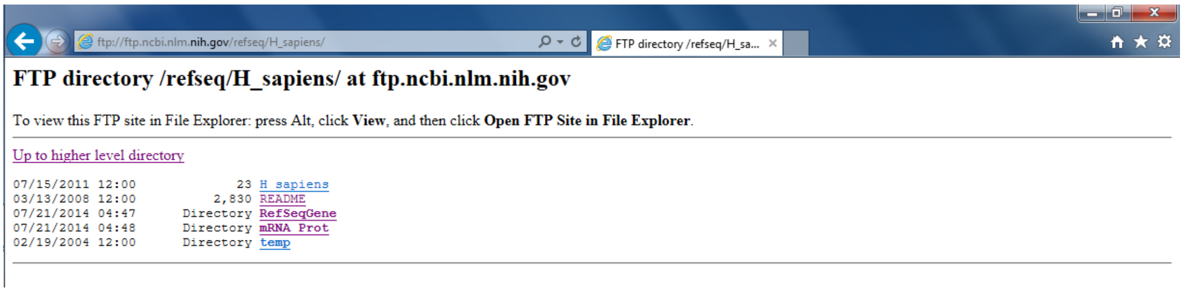
ftp://ftp.ncbi.nlm.nih.gov/refseq/H_sapiens/

FTP directory /refseq/H_sapiens/ at ftp.ncbi.nlm.nih.gov

To view this FTP site in File Explorer: press Alt, click **View**, and then click **Open FTP Site in File Explorer**.

Up to higher level directory

```
07/15/2011 12:00             23 H sapiens
03/13/2008 12:00          2,830 README
07/21/2014 04:47      Directory RefSeqGene
07/21/2014 04:48      Directory mRNA Prot
02/19/2004 12:00      Directory temp
```

Figure 65.

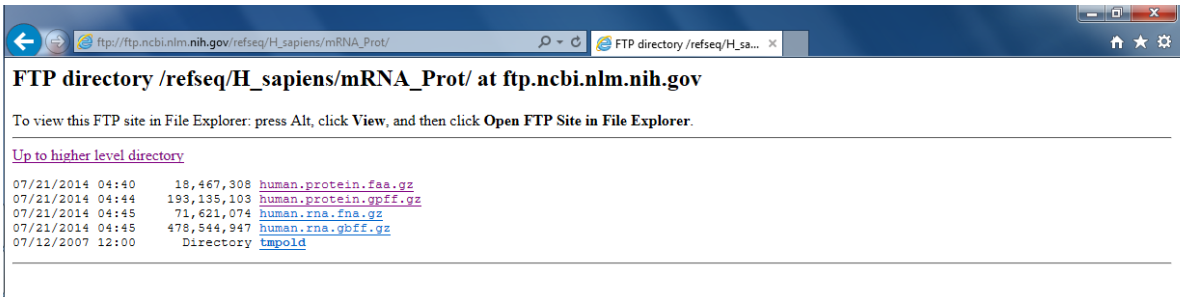
ftp://ftp.ncbi.nlm.nih.gov/refseq/H_sapiens/mRNA_Prot/

FTP directory /refseq/H_sapiens/mRNA_Prot/ at ftp.ncbi.nlm.nih.gov

To view this FTP site in File Explorer: press Alt, click **View**, and then click **Open FTP Site in File Explorer**.

Up to higher level directory

```
07/21/2014 04:40     18,467,308 human.protein.faa.gz
07/21/2014 04:44    193,135,103 human.protein.gpff.gz
07/21/2014 04:45     71,621,074 human.rna.fna.gz
07/21/2014 04:45    478,544,947 human.rna.gbff.gz
07/12/2007 12:00      Directory tmpold
```

Figure 66.

IPI

IPI protein databases are not updated anymore but are still used in the proteomics community. The IPI database repository can be accessed via the following website: http://www.ebi.ac.uk/IPI (Figure 67).

Select the "*last release*" folder and then the "*current*" folder (Figures 68 and 69). Two type of database files are compatible with PeptideManager: the dat and the fasta formats (Figure 70).

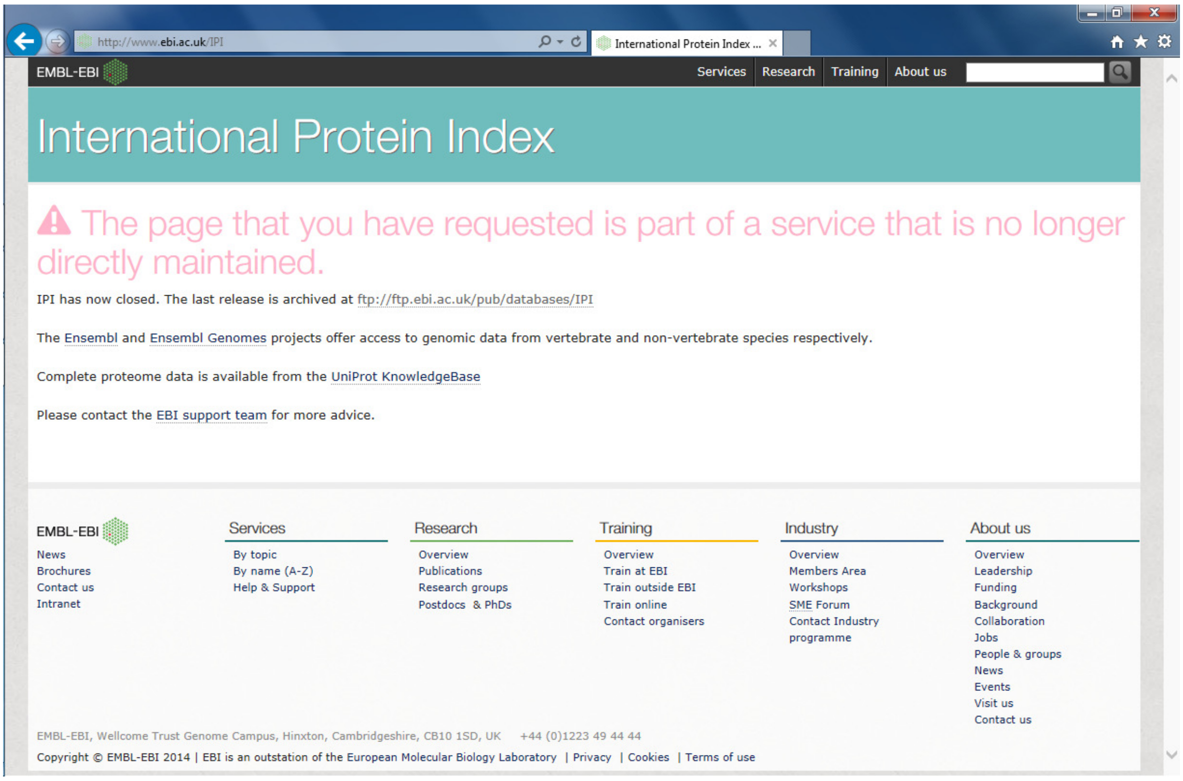

Figure 67.

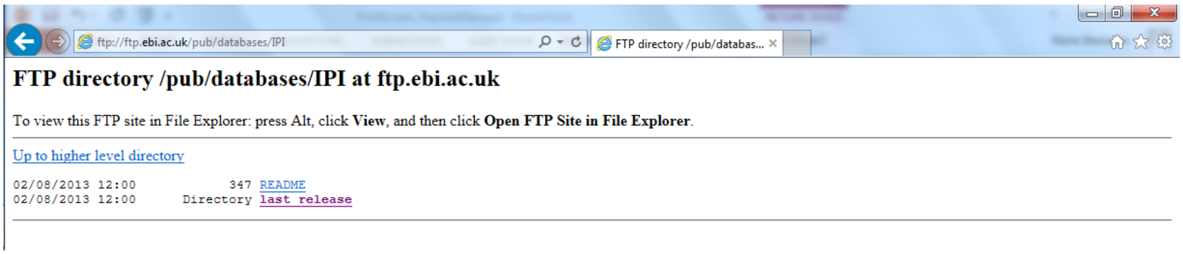

Figure 68.

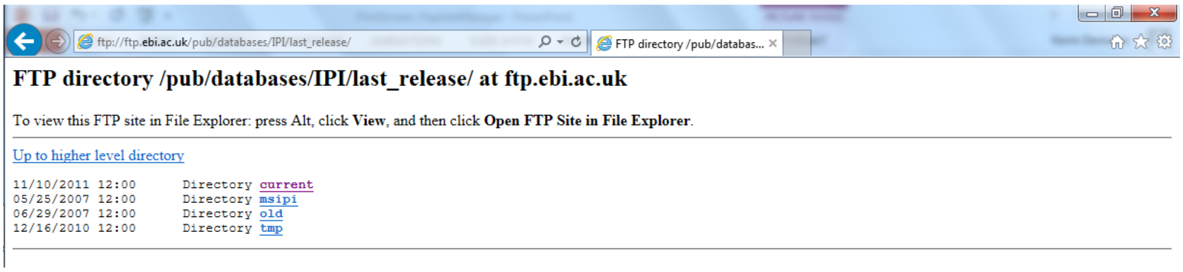

Figure 69.

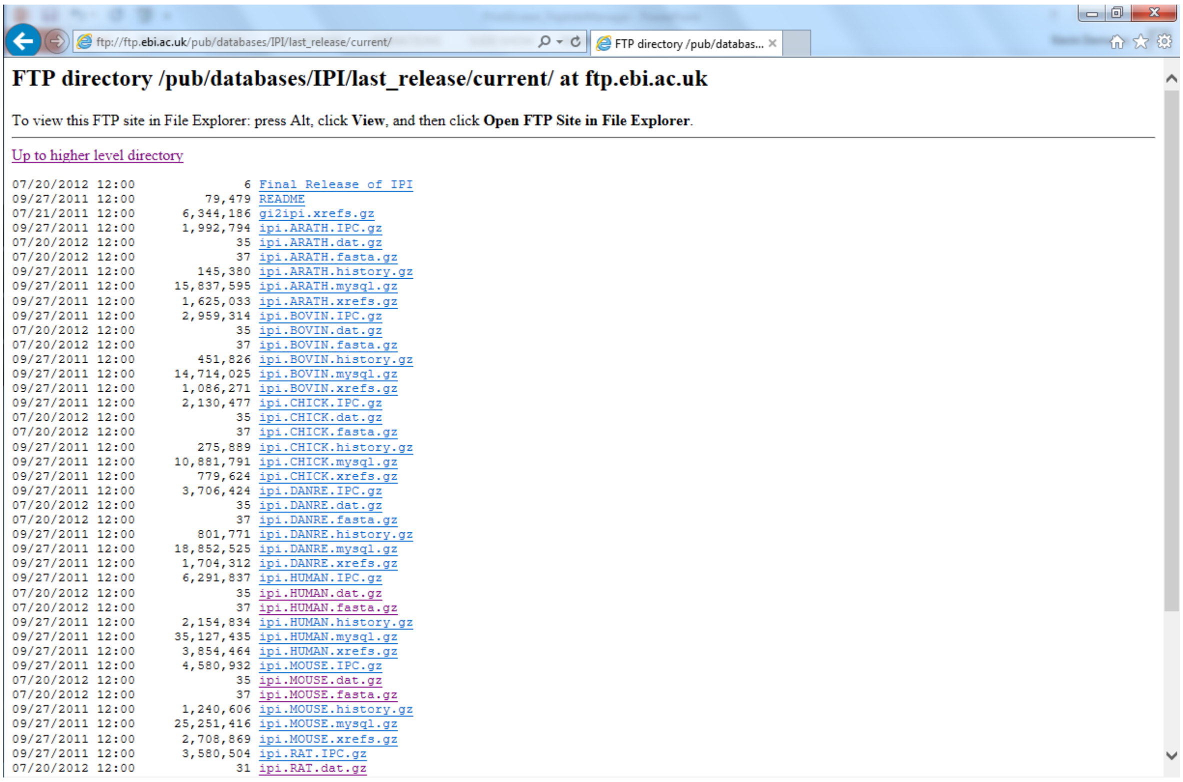

Figure 70.

Compare peptide/protein lists

The "*List Comparator*" tab of PeptideManger (Figure 71) allows you to compare any list of string (*e.g.*, peptide sequences, protein IDs). Just copy/paste the list of reference in the textbox "*List of Reference*" and the list to compare to the list of reference in the textbox "*List to Compare*". Check the "*Produce an output csv file*" checkbox if you want to save the results in a csv file and then click on the "**Compare**" button. The results are displayed in the data grid as shown in Figure 72.

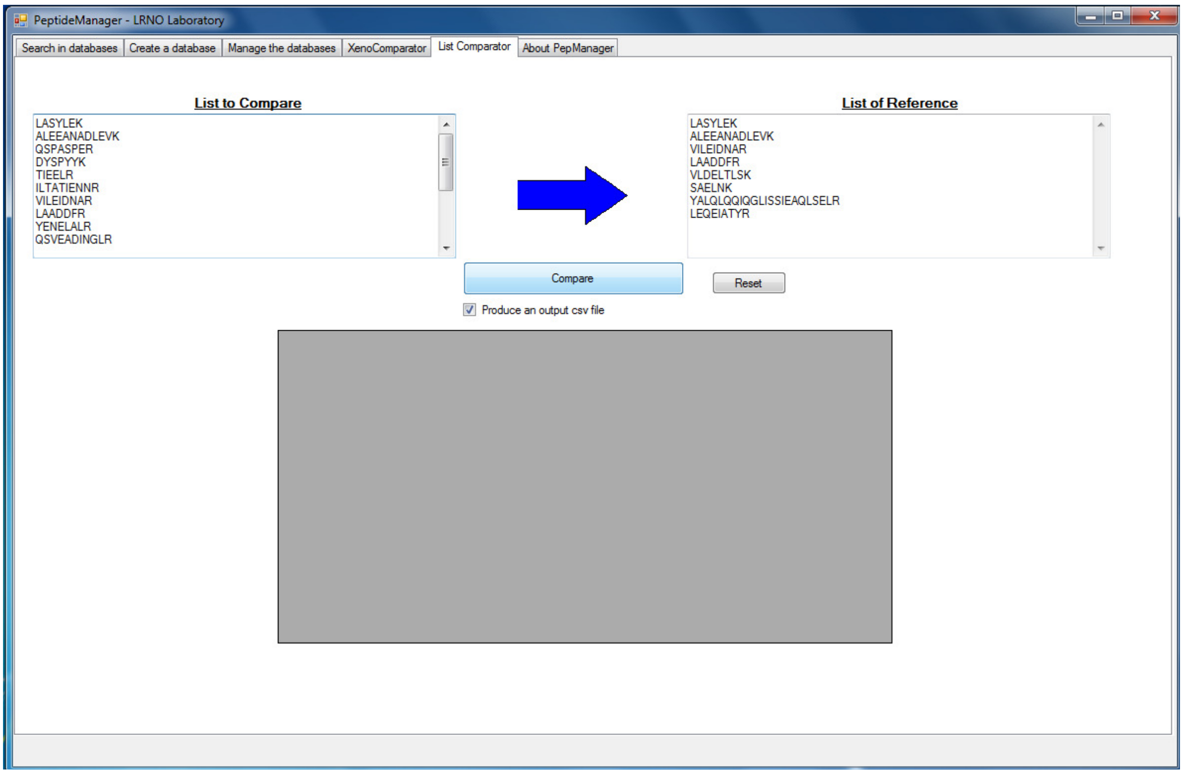

Figure 71.

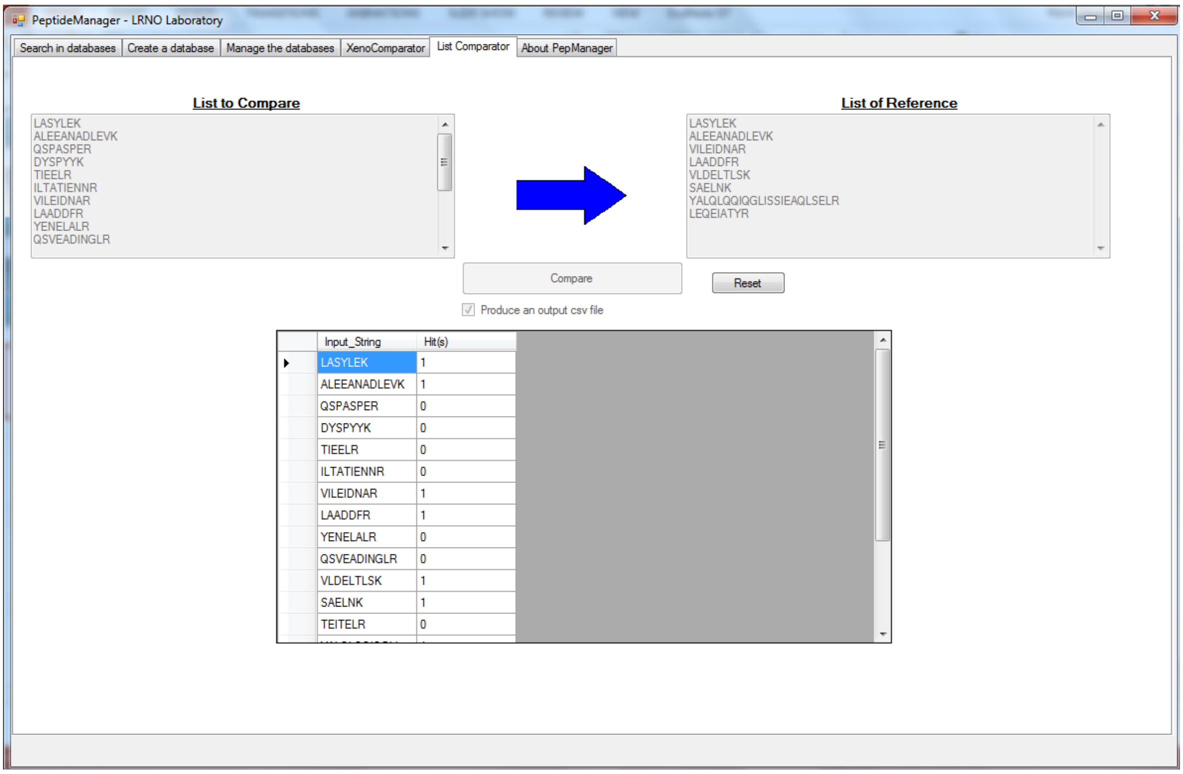

Figure 72.

Import a user-customized protein database

It is possible to build a PeptideManager database from scratch in order to produce a user-customized database.

You can directly create the database files which are SQLite database files whose structure is indicated in Figures 73, 74 and 75. The SQLite Manager add-on for Mozilla Firefox is an easy and light interface to edit SQLite files (https://addons.mozilla.org/en-US/firefox/addon/sqlite-manager/) (Figure 76). However, you will have to evaluate the occurrence of the peptide sequences within the database by an external mean.

Another way to build your user-customized database is to create a fasta-like format file of your protein sequences either in the faa format of RefSeq (Figure 77) or in the fasta format of IPI (Figure 78). This method has the advantage that the calculation of the occurrence of the peptide sequences within the database will be done.

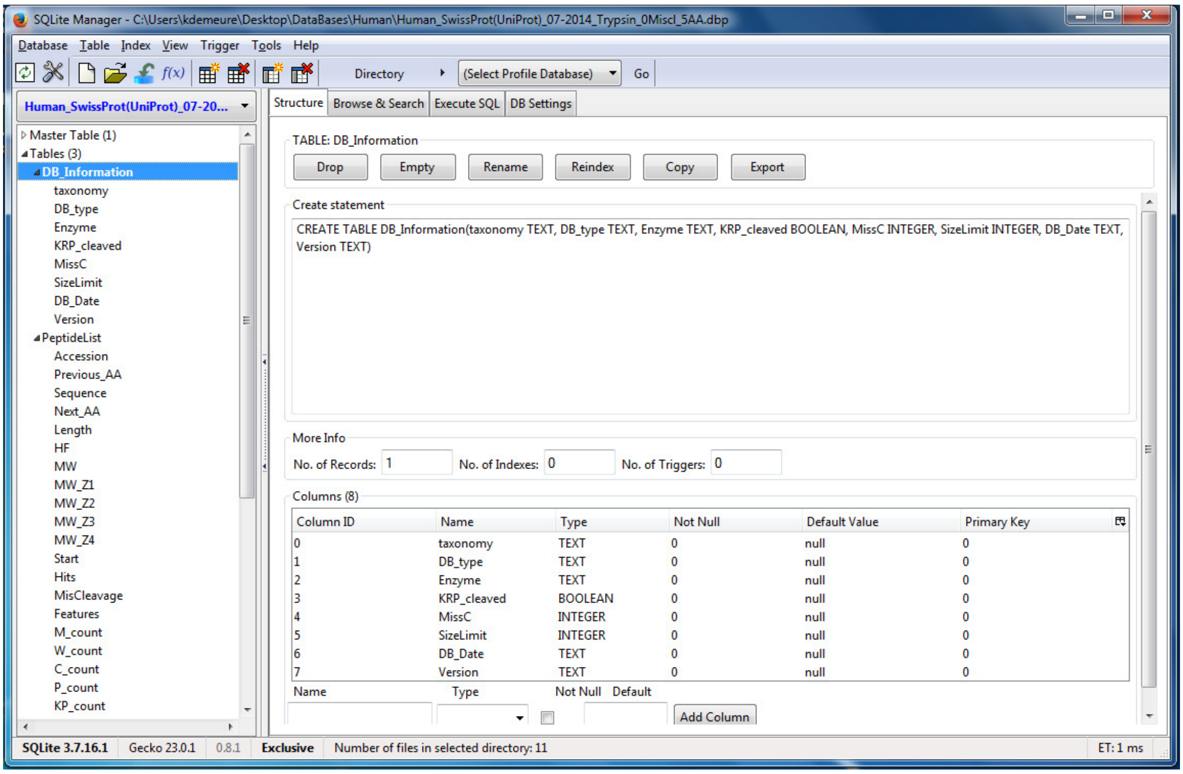

Figure 73.

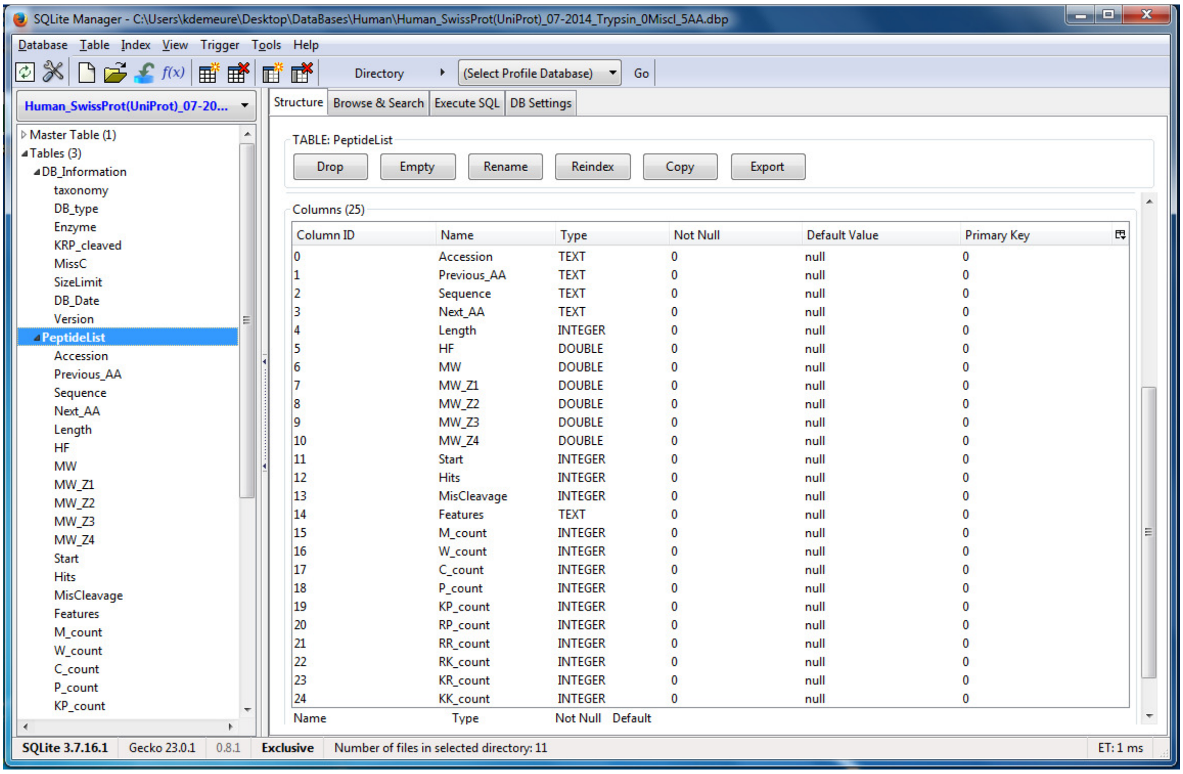

Figure 74.

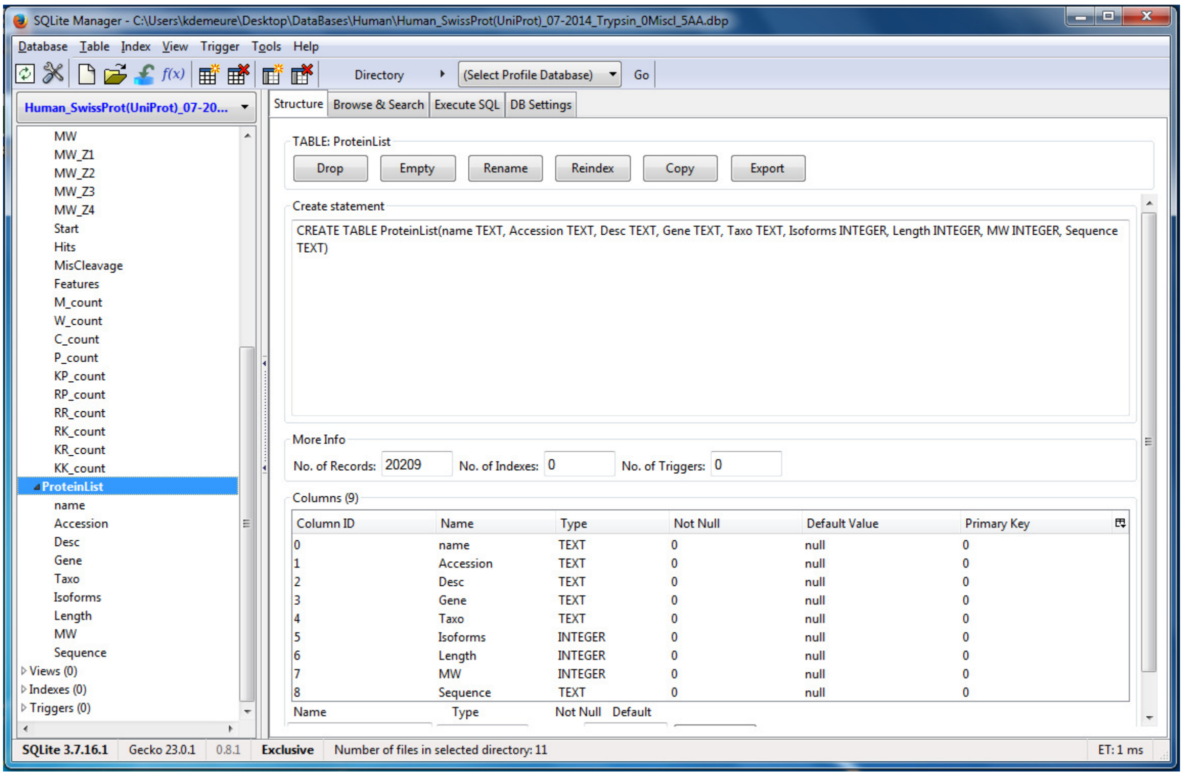

Figure 75.

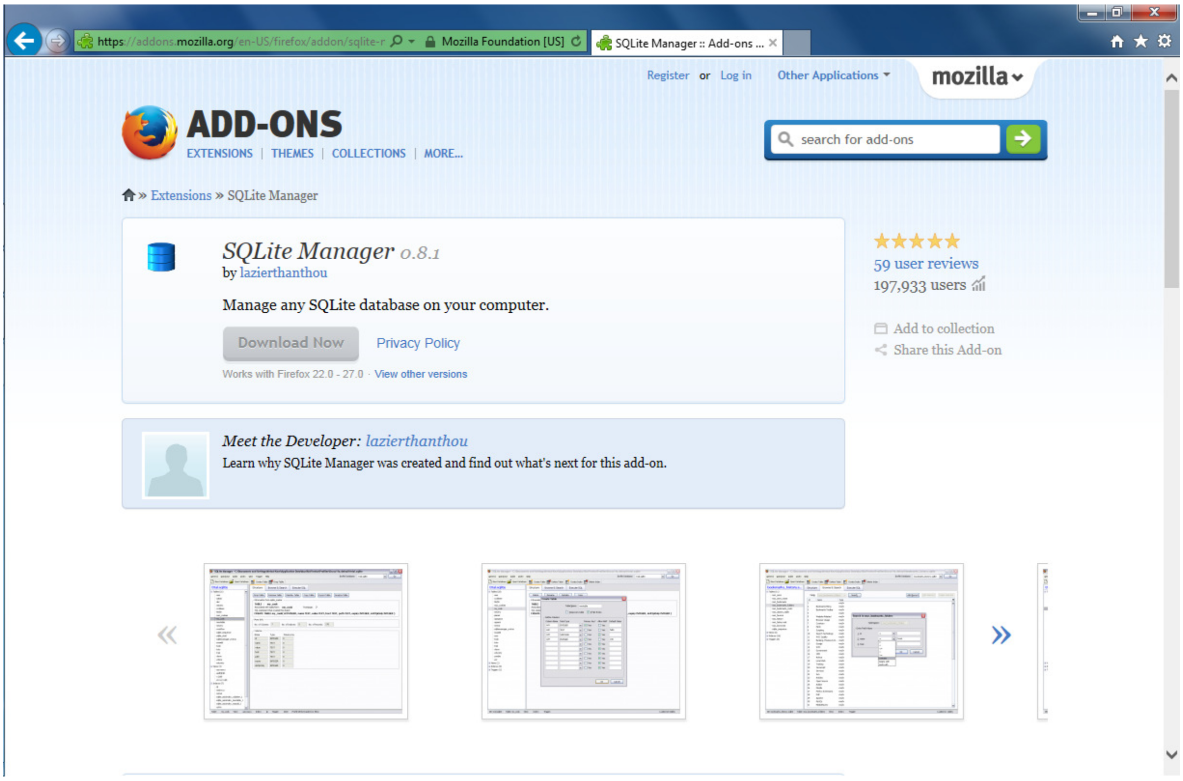

Figure 76.

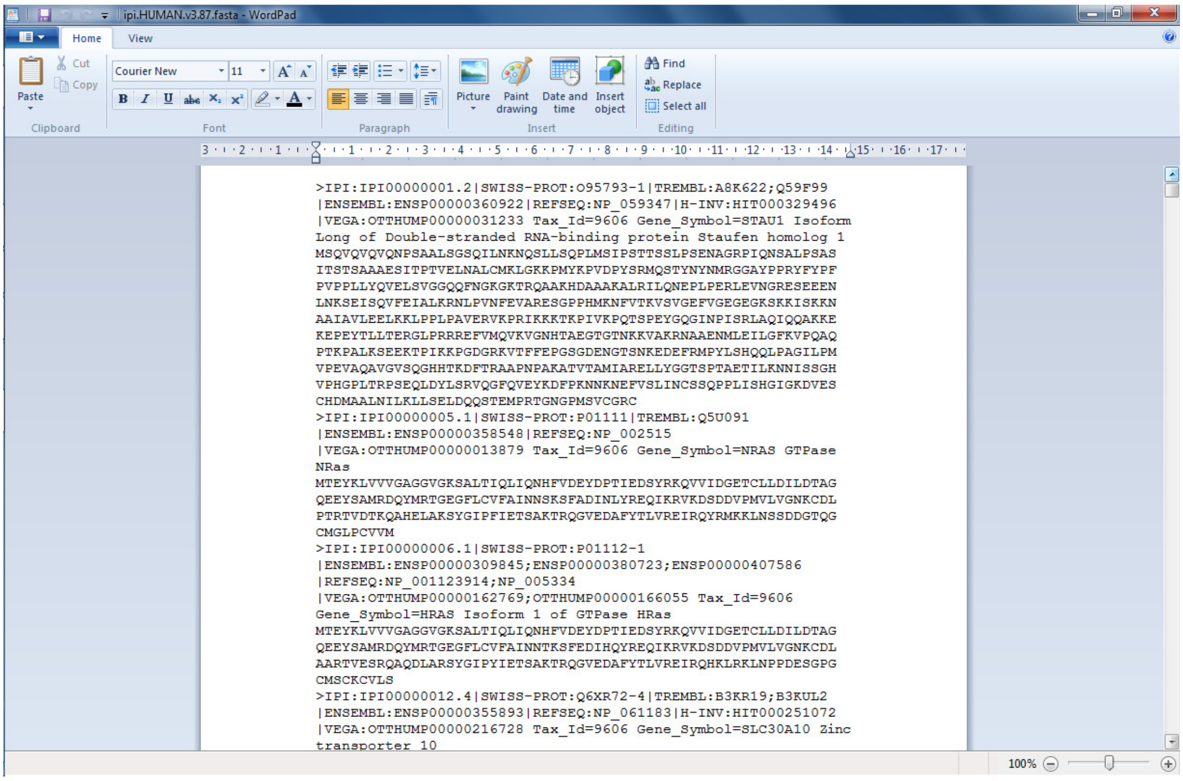


```
>IPI:IPI00000001.2|SWISS-PROT:O95793-1|TREMBL:A8K622;Q59F99
|ENSEMBL:ENSP00000360922|REFSEQ:NP_059347|H-INV:HIT000329496
|VEGA:OTTHUMP00000031233 Tax_Id=9606 Gene_Symbol=STAU1 Isoform
Long of Double-stranded RNA-binding protein Staufen homolog 1
MSQVQVQVQNPSAALSGSQILNKNQSLLSQPLMSIPSTTSSLPSENAGRPIQNSALPSAS
ITSTSAAAESITPTVELNALCMKLGKKPMYKPVDPYSRMQSTYNYNMRGGAYPPRYFYPF
PVPPLLYQVELSVGGQQFNGKGKTRQAAKHDAAAKALRILQNEPLPERLEVNGRESEEEN
LNKSEISQVFEIALKRNLPVNFEVARESGPPHMKNFVTKVSVGEFVGEGEGKSKKISKKN
AAIAVLEELKKLPPLPAVERVKPRIKKKTKPIVKPQTSPEYGQGINPISRLAQIQQAKKE
KEPEYTLLTERGLPRRREFVMQVKVGNHTAEGTGTNKKVAKRNAAENMLEILGFKVPQAQ
PTKPALKSEEKTPIKKPGDGRKVTFFEPGSGDENGTSNKEDEFRMPYLSHQQLPAGILPM
VPEVAQAVGVSQGHHTKDFTRAAPNPAKATVTAMIARELLYGGTSPTAETILKNNISSGH
VPHGPLTRPSEQLDYLSRVQGFQVEYKDFPKNNKNEFVSLINCSSQPPLISHGIGKDVES
CHDMAALNILKLLSELDQQSTEMPRTGNGPMSVCGRC
>IPI:IPI00000005.1|SWISS-PROT:P01111|TREMBL:Q5U091
|ENSEMBL:ENSP00000358548|REFSEQ:NP_002515
|VEGA:OTTHUMP00000013879 Tax_Id=9606 Gene_Symbol=NRAS GTPase
NRas
MTEYKLVVVGAGGVGKSALTIQLIQNHFVDEYDPTIEDSYRKQVVIDGETCLLDILDTAG
QEEYSAMRDQYMRTGEGFLCVFAINNSKSFADINLYREQIKRVKDSDDVPMVLVGNKCDL
PTRTVDTKQAHELAKSYGIPFIETSAKTRQGVEDAFYTLVREIRQYRMKKLNSSDDGTQG
CMGLPCVVM
>IPI:IPI00000006.1|SWISS-PROT:P01112-1
|ENSEMBL:ENSP00000309845;ENSP00000380723;ENSP00000407586
|REFSEQ:NP_001123914;NP_005334
|VEGA:OTTHUMP00000162769;OTTHUMP00000166055 Tax_Id=9606
Gene_Symbol=HRAS Isoform 1 of GTPase HRas
MTEYKLVVVGAGGVGKSALTIQLIQNHFVDEYDPTIEDSYRKQVVIDGETCLLDILDTAG
QEEYSAMRDQYMRTGEGFLCVFAINNTKSFEDIHQYREQIKRVKDSDDVPMVLVGNKCDL
AARTVESRQAQDLARSYGIPYIETSAKTRQGVEDAFYTLVREIRQHKLRKLNPPDESGPG
CMSCKCVLS
>IPI:IPI00000012.4|SWISS-PROT:Q6XR72-4|TREMBL:B3KR19;B3KUL2
|ENSEMBL:ENSP00000355893|REFSEQ:NP_061183|H-INV:HIT000251072
|VEGA:OTTHUMP00000216728 Tax_Id=9606 Gene_Symbol=SLC30A10 Zinc
transporter 10
```

Figure 77.

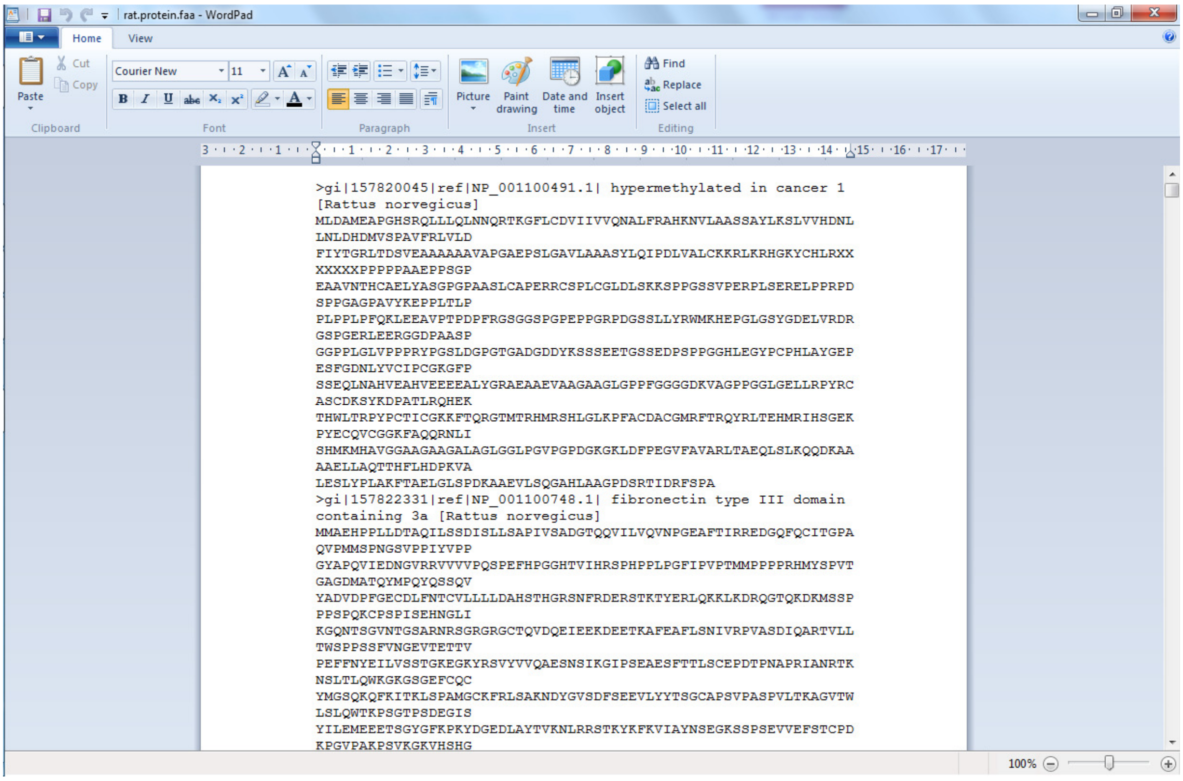


```
>gi|157820045|ref|NP_001100491.1| hypermethylated in cancer 1
[Rattus norvegicus]
MLDAMEAPGHSRQLLLQLNNQRTKGFLCDVIIVVQNALFRAHKNVLAASSAYLKSLVVHDNL
LNLDHDMVSPAVFRLVLD
FIYTGRLTDSVEAAAAAAVAPGAEPSLGAVLAAASYLQIPDLVALCKKRLKRHGKYCHLRXX
XXXXXPPPPPAAEPPSGP
EAAVNTHCAELYASGPGPAASLCAPERRCSPLCGLDLSKKSPPGSSVPERPLSERELPPRPD
SPPGAGPAVYKEPPLTLP
PLPPLPFQKLEEAVPTPDPFRGSGGSPGPEPPGRPDGSSLLYRWMKHEPGLGSYGDELVRDR
GSPGERLEERGGDPAASP
GGPPLGLVPPPRYPGSLDGPGTGADGDDYKSSSEETGSSEDPSPPGGHLEGYPCPHLAYGEP
ESFGDNLYVCIPCGKGFP
SSEQLNAHVEAHVEEEEALYGRAEAAEVAAGAAGLGPPFGGGGDKVAGPPGGLGELLRPYRC
ASCDKSYKDPATLRQHEK
THWLTRPYPCTICGKKFTQRGTMTRHMRSHLGLKPFACDACGMRFTRQYRLTEHMRIHSGEK
PYECQVCGGKFAQQRNLI
SHMKMHAVGGAAGAAGALAGLGGLPGVPGPDGKGKLDFPEGVFAVARLTAEQLSLKQQDKAA
AAELLAQTTHFLHDPKVA
LESLYPLAKFTAELGLSPDKAAEVLSQGAHLAAGPDSRTIDRFSPA
>gi|157822331|ref|NP_001100748.1| fibronectin type III domain
containing 3a [Rattus norvegicus]
MMAEHPPLLDTAQILSSDISLLSAPIVSADGTQQVILVQVNPGEAFTIRREDGQFQCITGPA
QVPMMSPNGSVPPIYVPP
GYAPQVIEDNGVRRVVVVPQSPEFHPGGHTVIHRSPHPPLPGFIPVPTMMPPPPRHMYSPVT
GAGDMATQYMPQYQSSQV
YADVDPFGECDLFNTCVLLLLDAHSTHGRSNFRDERSTKTYERLQKKLKDRQGTQKDKMSSP
PPSPQKCPSPISEHNGLI
KGQNTSGVNTGSARNRSGRGRGCTQVDQEIEEKDEETKAFEAFLSNIVRPVASDIQARTVLL
TWSPPSSFVNGEVTETTV
PEFFNYEILVSSTGKEGKYRSVYVVQAESNSIKGIPSEAESFTTLSCEPDTPNAPRIANRTK
NSLTLQWKGKGSGEFCQC
YMGSQKQFKITKLSPAMGCKFRLSAKNDYGVSDFSEEVLYYTSGCAPSVPASPVLTKAGVTW
LSLQWTKPSGTPSDEGIS
YILEMEEETSGYGFKPKYDGEDLAYTVKNLRRSTKYKFKVIAYNSEGKSSPSEVVEFSTCPD
KPGVPAKPSVKGKVHSHG
```

Figure 78.

Table of Contents

Install PeptideManager 3

First Launch 3

Build a peptide database 5

Manage the PeptideManager databases 9

Launch a search request 13

Search by Protein ID 13

Search by Protein Name 17

Search by Peptide Sequence(s) 19

Case Studies: Select unique peptide sequences for a targeted proteomics experiment 20

Unique peptide sequences selection – P12352 20

Peptide selection to differentiate protein isoforms 24

Monitoring post-translational modifications of interest 25

Select unique peptide sequences with the presence of a host/background proteome 26

Filtering Mode 27

Batch Mode 33

Case study: Selection of unique peptide sequences for a targeted proteomics experiment with the presence of a background/host proteome 36

Download the public protein databases 38

SwissProt/TrEMBL/UniProt 38

Predefined taxonomic divisions protein databases 38

Other taxonomic divisions 40

RefSeq 42

IPI 44

Compare peptide/protein lists 46

Import a user-customized protein database 47
